# Discovery of Ircinianin Lactones B and C—Two New Cyclic Sesterterpenes from the Marine Sponge *Ircinia wistarii*

**DOI:** 10.3390/md20080532

**Published:** 2022-08-19

**Authors:** Thomas Majer, Keshab Bhattarai, Jan Straetener, Justus Pohlmann, Patrick Cahill, Markus O. Zimmermann, Marc P. Hübner, Marcel Kaiser, Johan Svenson, Michael Schindler, Heike Brötz-Oesterhelt, Frank M. Boeckler, Harald Gross

**Affiliations:** 1 Department of Pharmaceutical Biology, Pharmaceutical Institute, University of Tübingen, Auf der Morgenstelle 8, 72076 Tübingen, Germany; 2 Department of Microbial Bioactive Compounds, Interfaculty Institute of Microbiology and Infection Medicine (IMIT), University of Tübingen, Auf der Morgenstelle 28, 72076 Tübingen, Germany; 3 Institute for Medical Virology and Epidemiology, Section Molecular Virology, University Hospital Tübingen, 72076 Tübingen, Germany; 4 Cawthron Institute, 98 Halifax Street East, Nelson 7010, New Zealand; 5 Lab for Molecular Design and Pharmaceutical Biophysics, Department of Pharmacy and Biochemistry, Institute of Pharmaceutical Sciences, University of Tübingen, Auf der Morgenstelle 8, 72076 Tübingen, Germany; 6 Institute for Medical Microbiology, Immunology and Parasitology, University Hospital Bonn, 53127 Bonn, Germany; 7 German Center for Infection Research (DZIF), Partner Site Bonn-Cologne, Bonn, Germany; 8 Swiss Tropical and Public Health Institute, Kreuzstrasse 2, 4123 Allschwil, Switzerland; 9 Faculty of Science, University of Basel, Petersplatz 1, 4002 Basel, Switzerland; 10 Cluster of Excellence ‘Controlling Microbes to Fight Infections’, University of Tübingen, 72076 Tübingen, Germany; 11 German Center for Infection Research (DZIF), Partner Site Tübingen, Tübingen, Germany; 12 Interfaculty Institute for Biomedical Informatics (IBMI), University of Tübingen, Sand 14, 72076 Tübingen, Germany

**Keywords:** sponge, *Ircinia wistarii*, sesterterpene, ircinianin-derivates, bioactivity screening, antiprotozoal activity, *Plasmodium falciparum*, *Leishmania donovani*, NCI-60

## Abstract

Two new ircinianin-type sesterterpenoids, ircinianin lactone B and ircinianin lactone C (**7** and **8**), together with five known entities from the ircinianin compound family (**1**, **3**–**6**) were isolated from the marine sponge *Ircinia wistarii*. Ircinianin lactones B and C (**7** and **8**) represent new ircinianin terpenoids with a modified oxidation pattern. Despite their labile nature, the structures could be established using a combination of spectroscopic data, including HRESIMS and 1D/2D NMR techniques, as well as computational chemistry and quantum-mechanical calculations. In a broad screening approach for biological activity, the class-defining compound ircinianin (**1**) showed moderate antiprotozoal activity against *Plasmodium falciparum* (IC_50_ 25.4 μM) and *Leishmania donovani* (IC_50_ 16.6 μM).

## 1. Introduction

Marine sponges are a unique source for natural products, and even after decades of research, they still represent a rich reservoir of novel chemistry [1]. The phylum Porifera produces a huge variety of different natural product classes, and the genus *Ircinia* is well known for producing sesterterpenes (C_25_-terpenes) [2,3,4,5,6,7,8,9]. The chemical scaffolds of these terpenoids show a huge diversity with significant biological activity in almost all fields of biomedical research [10,11,12,13,14,15]. Furthermore, as antifouling bioactives and fish predation deterrents, they also play an important role in the chemical defense architecture of *Ircinia* within the marine ecosystem [2,16,17].

**Figure 1:**
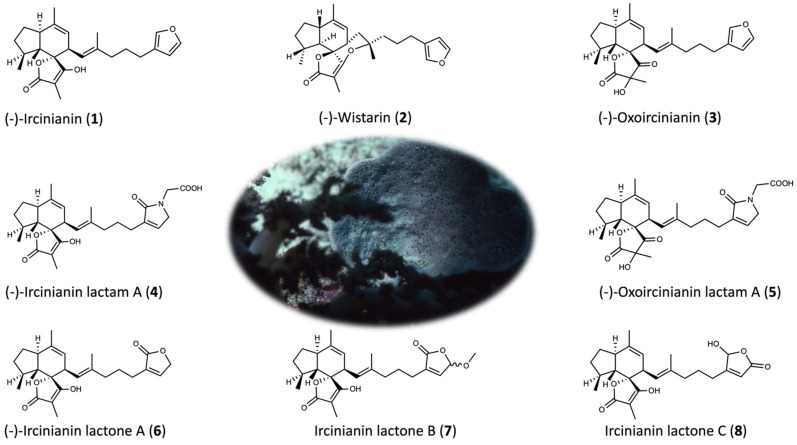
Structures of the ircinianin-sesterterpene family. The center image depicts the *Ircinia wistarii* sample of this study.

C_25_-terpenes of *Ircinia* species can be classified into linear and (poly)carbocyclic forms [18]. Members of the linear class include the variabilin family, ircinialactams, irciformonins and the ircinins. They show a characteristic linear alkyl-diene motif with a furan, lactone, lactam and/or a terminal tetronic acid subunit, whereby in most cases, two of these heterocycles are part of the molecular architecture [6,7,13,19]. In contrast, the cyclic group is formed out of a carbocyclic core skeleton, commonly attached to a linear, sometimes diene-containing, alkyl moiety with a furan, lactam, lactone or tetronic acid moiety at its terminus. Based on the number of cyclic groups incorporated in the core motive, it is possible to subdivide the compounds into several additional subclasses [2,4,15,20,21,22]. Ircinianins belong to the bicarbocyclic furano sesterterpene subclass, for which ircinianin (**1**) is the class-defining molecule (Figure 1). This subclass bares a rare indene-spirotetronic acid core system.

Ircinianin (**1**) was firstly described in 1977 by Hofheinz and Schönholzer as a secondary metabolite from an *Ircinia* sp. collected near Heron Island, Wistari reef, Australia [23].

The structure was solved by X-ray analysis, but due to methodological limitations at that time, only the relative stereochemistry could be assigned [23]. A subsequent biogenetic synthesis of (±)-ircinianin in 1986 provided evidence that an intramolecular Diels–Alder reaction is involved in the biosynthetic pathway of **1** [24]. In the 1990’s, the structure was confirmed by total synthesis and the full stereostructure was assigned [25]. In 2020, natural-derived ircinianin (**1**) was effectively crystalized to enable confirmation of the absolute structure [26].

Besides ircinianin, further derivatives of **1** were isolated, such as ircinianin sulfate and wistarin (**2**), the tetracyclic isomer of **1** [27,28,29,30]. In 2013, within the frame of a screening program for glycin receptor (GlyR) modulators, the Capon Group rediscovered these molecules from the marine sponge *Psammocinia* sp. [30]. They expanded the known chemical space of the class by the characterization of new congeners and demonstrated that derivatives selectively act either as α1 or α3 GlyR potentiators. The new derivatives had lactam- (**4** and **5**) and lactone-based (**6**) five-membered ring systems instead of terminal furan rings of other known C_25_ terpenes. They also had oxo-tetronic acid forms (**3** and **5**), which can be delineated from hydroxy-keto-enol tautomerism.

During our early work to isolate and determine a refinded X-ray structure of ircinianin (**1**) from the exisiting *Ircinia wistarii* sample [26], it became apparent that several ircinianin derivatives were present in adjacent fractions. The current study describes the work-up of these fractions, which led to the isolation and structural elucidation of two new ircinianin lactones (**7** and **8**), along with a suite of ircinianins previously reported that include oxo-ircinianin (**3**), ircinianin lactam A (**4**), oxoircinianin lactam A (**5**) and ircinianin lactone A (**6**). Since from our first study [26], high amounts of crystalline ircinianin (**1**) were still available, an in-depth screen for biological activity was conducted in parallel, which led to the discovery of a moderate antiprotozoal activity for **1**.

## 2. Results and Discussion

### 2.1. Isolation and Structure Elucidation

Existing RP-LC fractions from a former study [26] were chromatographed repeatedly over RP-HPLC to yield pure compounds **3**–**8**, respectively. The known compounds were readily identified via HR-MS and MS^n^ spectroscopic measurements or by comparing their NMR data with those reported in the literature (see Supporting Information) [30].

**Figure 2:**
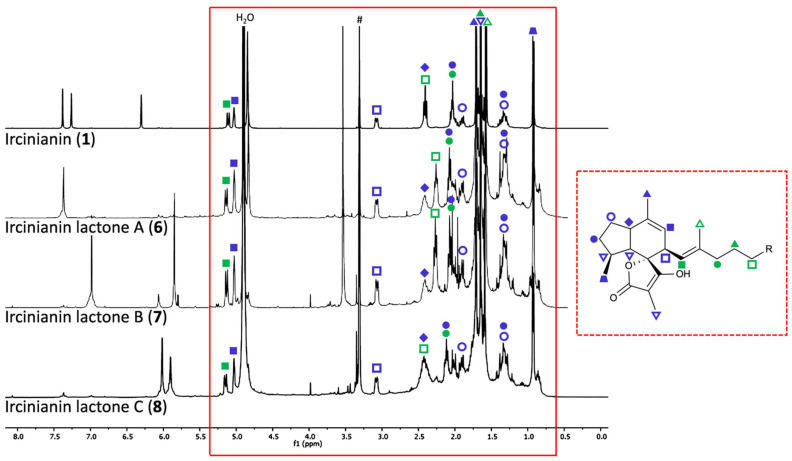
Stacked ^1^H-NMR spectra of ircinianin (**1**) and ircinianin lactones A-C (**6**–**8**), recorded in *d*_4_-MeOH with the characteristic “signal-fingerprint” of the ircinianin-like scaffold in the 0.6 to 5.4 ppm region (red box with solid lines). The signals of the sidechain heterocycles appear between 5.5 to 7.5 ppm. Symbols denote the peak assignments in all ^1^H NMR spectra, which are visualized in the chemical structure on the right (red box with dashed lines). Key: blue symbols denote hydrogens of the tricyclic spirotetronic acid backbone; green symbols denote hydrogens of the alkyl side chain, R = terminal sidechain heterocycle. Hash symbol denotes residual methanol.

**Figure 3:**
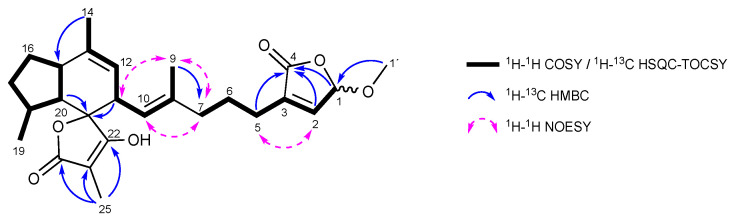
Key NMR correlations for **7**.

The molecular formula of **7** was established as C_26_H_35_O_6_ by (+)-HRESIMS ([M + H]^+^ 443.2431, calcd. 443.2428, see Figure S33), requiring ten double bound equivalents. The ^1^H NMR spectroscopic data exhibited resonances typical of an ircinianin-type metabolite (Figure 2). For example, four methyl resonances (δ_H_ 0.92, 1.57, 1.63, 1.70), a sp^3^ methine doublet of multiplet signal (δ_H_ 3.06), two olefinic resonances (δ_H_ 5.01 and 5.12) and a methylene envelope (δ_H_ 1.25–2.50) are indicative of the tricyclic spirotetronic acid backbone including an alkyl chain in **7**. A detailed comparison of the ^1^H and ^13^C spectra of **7** with known ircinianin compounds revealed that they were almost identical with those of ircinianin lactone A (**6**) (Table 1 and Table S4, Figure 2, Figure S34 and Figure S35). However, the ^1^H and ^13^C spectra showed characteristic signals of an additional methoxy (δ_H/C_ 3.52/57.1) and a hemiacetal methine group (δ_H/C_ 5.84/104.4), while H-2 experienced a high-field shift in the ^1^H NMR spectrum and the absence of CH_2_-1 was noted in the ^13^C NMR and DEPT135 spectrum, respectively. This led to the conclusion that C-1 had been oxidized and subsequently methoxylated. These findings were further corroborated by 2D NMR experiments, particularly by ^1^H-^13^C HMBC cross correlations between the methoxy methyl protons H_3_-1′ and the hemiacetal methine group C-1 (Figure 3), and between H-1 and C-4, as well as by coupling between H-1/H-2 and H-2/H-3, detected in the ^1^H-^1^H-COSY spectrum. The observed ^1^H-^1^H-NOESY correlations H-2/H_2_-5 provided further evidence that **7** bears an α-substituted γ-methoxy-γ-butenolide ring system. The comparison of the NMR shift values was consistent with related natural metabolites known to contain this moiety [31,32,33].

**Figure 4:**
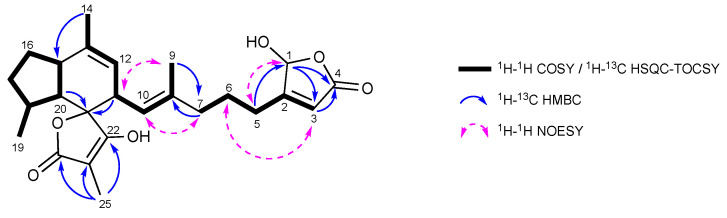
Key NMR correlations for **8**.

With the planar structure of **7** determined, the geometry of the double bond of the alkene chain and the chiral centers was inferred. Based on the upfield ^13^C NMR chemical shift for the olefinic methyl group C-9 (δ_c_ 16.1) [34] and ^1^H-^1^H-NOESY correlations between H-7a/H-10, H_3_-9/H-7b and H_3_-9/H-11 (Figure 3 and Figure S43), the geometry of the Δ^8,10^ double bond was defined as *E*. Further diagnostic NOE correlations between the resonances H-10/H-20, H-19/H-20, H-20/H-16b, H-16b/H_3_-14, H-16a/H-15 and H-15/H-18 (Figure S43B) were indicative of the relative configuration of the indene portion of **7** as 11R*, 15R*, 18S* and 20R*. Since the tetronic acid ring is located perpendicular to the indene system and is hydrogen deficient, no NOE contacts were produced and no conclusions could be drawn regarding the absolute configuration at C-21. However, **7** shares the same carbon skeleton with **1**, except for the terminal heterocycle. The absolute configuration of the carbon skeleton of **1**, isolated from the same existing sponge sample (HER6), has previously been determined by X-ray crystallography, and in this study, assured by CD-spectroscopy (see Figure S8). Therefore, the nearly identical NMR values for C-21 strongly imply that both molecules are in the *S*-configuration at this position, and this is also the most plausible solution for biogenetic reasons. Concerning the configuration at position C-1, it must be considered that **7** is an approximately 1:1 mixture of C-1 epimers, and these epimers should be interchangeable via an acyclic from. Notably, the epimers differed in chemical shifts not only of carbon atoms close to the epimeric center but stretching from C-2 to C-10 (Table 1). This suggests a close proximity of the butenolide ring and the alkene chain. This phenomenon has been also observed in other terpenoids bearing a γ-methoxy-α-substituted-α,β-unsaturated-γ-lactone group [32]. Based on these findings, **7** is the 1-methoxy-derivate of **6** and the trivial name ircinianin lactone B is proposed.

(+)-HRESIMS of ircinianin lactone C (**8**) showed a molecular ion consistent with the molecular formula of C_25_H_33_O_6_ ([M + H]^+^ 429.2281; calcd. 429.2272, see Figure S44) with ten degrees of unsaturation. Comparison of its ^1^H NMR spectral data with those for compounds **1**, **6** and **7** clearly shows it belongs to the ircinianin group (Figure 2). Detailed inspection of the ^1^H and ^13^C NMR data unveiled that the shift resonances for position 5 to position 25 in **8** were virtually identical to those for **1** (Table 1). Thus, **8** shares the same carbon framework within this portion of the molecule. The presence and connectivity of this typical structural fragment was corroborated by interpretation of the corresponding ^1^H-^13^C HSQC-TOCSY, ^1^H-^1^H COSY ^1^H-^13^C HMBC 2D NMR spectra (Figure 4).

The most notable difference in the NMR data between **1** and **8** was centered around the terminal heterocycle (Table S5). The furan ring system of **1** appears to be replaced by a C_4_H_3_O_3_ moiety in **8**. The latter consisted of one ester carbonyl (δ_C_ 173.8), one sp^2^ quaternary carbon (δ_C_ 172.7), one sp^2^ methine (δ_H/C_ 5.90/117.9) and one hemiacetal sp*^3^* methine (δ_H/C_ 6.02/101.1) [15,35] atom. Considering the remaining three degrees of unsaturation, the C_4_H_3_O_3_ fragment had to be cyclic, most likely via an ester-carbonyl-hemiacetal-linkage, thereby forming a five-membered lactone ring system. ^1^H-^13^C long range coupling between H-1 and C-4 confirmed the presence of the assumed lactone system. Furthermore, the ^1^H-^13^C HSQC-TOCSY-based analysis of the spin systems in the heteroaromatic formation indicated that the two protons at δ_H_ 6.02 (C-1) and 5.90 (C-3) were not part of the same spin system, but rather exhibited W-coupling (^4^*J*_H,H_ = 2.3 Hz). This observation, together with the ^1^H-^13^C HMBC correlation from H-5 to C-1, reveals that the arrangement of these three carbons consists of two separated spin systems. The spin systems are separated by C-2, which is in turn bound to C-5. The observed ^1^H–^13^C coupling between H-1/C-3 and H-3/C-4 in the HMBC spectrum of **8** led to completion of the terminal heterocycle and its identification as ß-substituted γ-hydroxy-γ-butenolide moiety.

The shift values of the terminal heterocycle were in very good agreement with literature data from similar natural products, bearing a γ-hydroxy-γ-butenolide moiety [15,31,36]. Analogous to compound **7**, the Δ^8,10^ double bond of **8** was defined as *E* (C-9: δ_C_ 16.2 and NOESY cross correlations depicted in Figure 4). Likewise, based on the same observed diagnostic NOE crosspeaks for the indene system (Figure S43B) and for biogenetic reasons, we propose that **8** is 11R*, 15R*, 18S*, 20R*, 21S* configured. Regrettably, like compounds **1** and **3**–**7**, compound **8** also proved to be unstable and underwent rapid decomposition after the NMR measurements, which precluded the experimental determination of the configuration at C-1. Therefore, we integrated computational methods and aimed for quantum-mechanical predictions of ^1^H and ^13^C NMR chemical shifts of a 1*S*- and a 1*R*-configured version of **8**.

**Figure 5:**
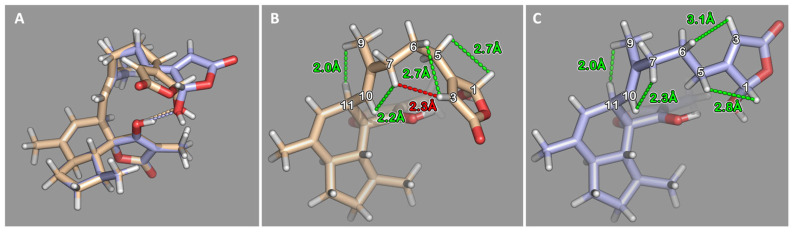
(**A**) Superimposition of the lowest energy conformer obtained for the 1*S*- (purple) and 1*R*-(brown) epimer of ircinianin lactone C (**8**). Dashed lines indicate the hydrogen bonds formed by OH-1 and OH-22. (**B**) 1*R*-epimer of **8**. Dashed lines indicate distance measurements; green through space interactions are in agreement with observed NOE correlations, while the red contact was expected to be detectable, but was absent in the corresponding NOESY NMR spectrum. (**C**) 1*S*-epimer of **8**. Green dashed lines indicate through space interactions, which agree with the observed NOE correlations.

Based on their moderate flexibility, both epimers were subjected to a conformational search using a stochastic algorithm in MOE 2018.0101. The potential energy surface was sampled by randomly perturbating rotatable bonds before minimizing the energy with the MMFF94x force field. The inversion of stereo centers and modification of double bond configurations were prohibited for obvious reasons. The termination criteria for both conformational searches were significantly increased from standard values to ensure that all relevant conformations within an energy cutoff of 20 kcal/mol above the detected global minimum were retrieved. Conformations with RMSD < 0.25 for all heavy atoms (carbon and oxygen) were considered duplicates. For each epimer, 30 distinct conformations were found and submitted to a free geometry optimization using B3-LYP/def2-SV(P), employing Grimme’s dispersion correction D3 in TURBOMOLE 7.4.1. Using the COSMO solvation model to approximate the experimental conditions, the dielectric constant was set to an epsilon of 32.7 for methanol at 25 °C. The conformations with lowest energies for both epimers are shown in Figure 5. They consistently form a hydrogen bond donated from the hydroxyl function of the spirotetronic acid motif toward the hydroxyl oxygen in position 1 of the side chain lactone ring with a distance between both oxygen atoms of 2.7 Å and 2.6 Å for the 1*S*-epimer and 1*R*-epimer, respectively. To facilitate such excellent hydrogen bond geometries, the side chains orientate the lactone ring in opposing directions for both epimers.

Nuclear magnetic shielding constants were calculated for all conformers using mpshift within TURBOMOLE, which employs the GIAO (Gauge Including Atomic Orbital) method. NMR shifts were obtained by comparing the nuclear shieldings of all conformers with the reference molecule TMS. The results were analyzed for each conformer, as well as for the Boltzmann-weighted conformational ensemble. For the best conformation of the 1*R*-epimer, a coefficient of determination (R^2^) of 0.998 and 0.993 was found for a linear fit of the calculated versus experimentally determined ^13^C chemical shifts and ^1^H chemical shifts, respectively, with a standard error s_e_ (δ) of 2.40 ppm and 0.12 ppm for ^13^C and ^1^H, respectively. The RMSD value for ^13^C was 4.73 ppm, and for ^1^H, it was 0.24 ppm. Being the dominant conformer in the Boltzmann-weighted ensemble of this epimer, the values do not change significantly for the ensemble: R^2^(^13^C) = 0.998; s_e_(^13^C) = 2.36 ppm; RMSD(^13^C) = 4.58 ppm; R^2^(^1^H) = 0.994; s_e_(^1^H) = 0.11 ppm; RMSD(^1^H) = 0.24 ppm. The best three conformations of the 1*S*-epimer are virtually identical in structure and energy. The average RMSD of their coordinates is only 0.069 and their energies differ by less than 0.2 kJ/mol. They absolutely dominate the Boltzmann-weighted conformational ensemble with a cumulative probability of 99.5% for these three microspecies. The resulting statistics for the Boltzmann-weighted ensemble of the 1*S*-epimer are: R^2^(^13^C) = 0.998; s_e_(^13^C) = 2.45 ppm; RMSD(^13^C) = 4.60 ppm; R^2^(^1^H) = 0.994; s_e_(^1^H) = 0.11 ppm; RMSD(^1^H) = 0.26 ppm. While the calculated chemical shifts are reasonably in line with the experimental values, we unfortunately did not observe clear evidence from this approach showing that only one of the epimers represents the experimentally observed chemical shifts correctly. This is likewise demonstrated by the strong correlation between the calculated chemical shifts for both Boltzmann-weighted ensembles of the epimers: A coefficient of determination (R^2^) of 0.9997 and 0.9962 was found for a linear fit of the calculated ^13^C chemical shifts and ^1^H chemical shifts, respectively.

However, the dominant species of both epimers could be differentiated by characteristic proton–proton distances in the side chain and their matching to experimentally obtained NOESY-NMR data (Figure 4 and Table 1). In the dominant species of the 1*S*-epimer (Figure 5C), short distances between protons attached to positions 1 and 5 (2.8 Å), positions 3 and 6 (3.1 Å), positions 7 and 10 (2.3 Å), and positions 9 and 11 (2.0 Å) are in line with observed NOE correlations. Likewise, the short distances between protons attached to positions 1 and 5 (2.7 Å), positions 3 and 6 (2.7 Å), positions 7 and 10 (2.2 Å), and positions 9 and 11 (2.0 Å) are present in the dominant microspecies of the 1*R*-epimer. Still, another short distance between protons attached to positions 3 and 7 (2.3 Å) cannot be found in NOESY spectrum of ircinianin lactone C. In conclusion, the conformational analysis followed by quantum mechanical calculations in comparison to observed NOE data strongly suggest that C-1 of **8** is *S*-configured. Compound **8** is the third member of the ircinianin lactone family and the trivial name ircinianin lactone C is proposed.

**Figure 6:**
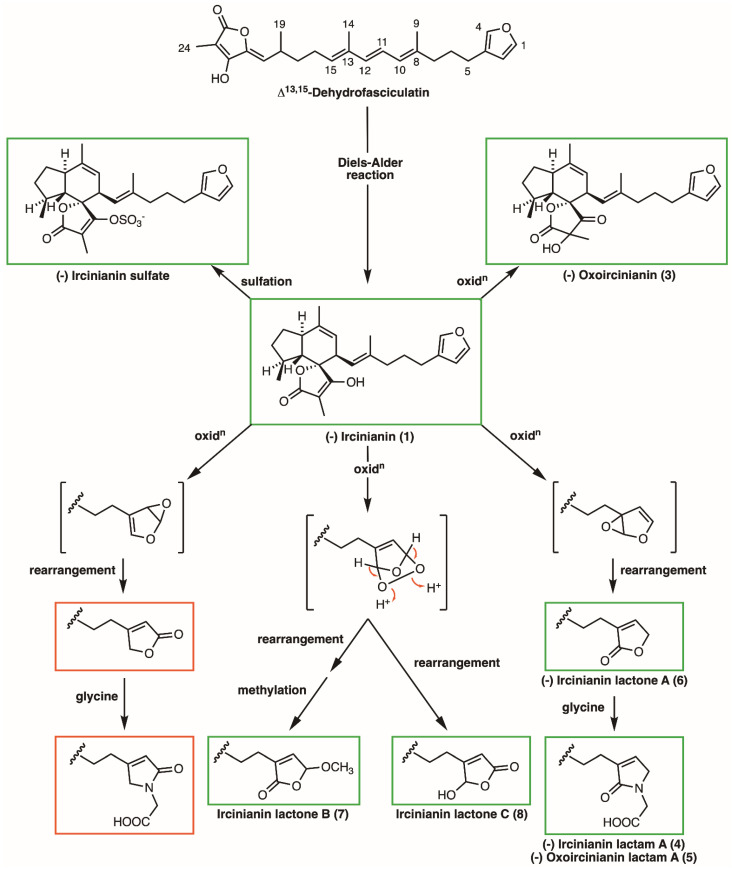
Proposed biosynthetic pathway. Substructures in green boxes are associated with isolated molecules, as mentioned above. Red-framed structures indicate what are so far only hypothetical structure analogues following the predicted biosynthetic scheme.

The structural complexity of the ircinianin architecture includes a polycyclic spirotetronic acid motif together with a plethora of variations in the sidechain. This architecture is unique within the C_25_-terpenoid class, inferring associated unique biosynthetic pathways (see Figure 6).

Based on a biomimetic total synthesis, it is assumed that the common tricyclic core motive can be formed out of a linear triene precursor via an intramolecular Diels–Alder reaction through enzymatic or thermal catalysis [23,24,37]. A further key reaction in the biosynthesis of ircinianins is partial oxidation, which often leads to intramolecular cyclization and rearrangements. These modifications give rise to the formation of five-membered heterocycles, such as furans and lactones. This process is the basis for the proposed biosynthetic relationship of the α,β-substituted sidechain furans, lactams and lactones, which often have been described for C_25_-terpenoids isolated from *Ircinia* species [37]. Capon and coworkers also hypothesized a biosynthetic pathway for converting similar small cyclic heteroaromatic motives into each other, where ircinianin is the progenitor of the molecule family [30]. In a follow-up study, they isolated ircinialactone A, a linear furano tetronic acid sesterterpene, and assumed that mono-epoxidation of the furan moiety through alternate oxidation potentially followed by nucleophilic addition reactions can form α,β unsaturated lactones and lactams [38]. However, these deliberations alone were not able to explain how a β-substituted γ-hydroxy-γ-butenolide motif, which was assigned in **8**, could have been generated from **1**. In order to close this gap of knowledge, a detailed literature review focused on biosynthetic pathways of structurally related moieties in linear sesterterpenes of marine origin was conducted next.

Given this conjecture, a detailed literature review focused on biosynthetic pathways of structurally related moieties in linear sesterterpenes of marine origin was conducted. A highly relevant hypothetical pathway was described by Khushi and coworkers during the chemical evaluation of a new linear hydroxy-butenolide sesterterpene family, called cacolides, isolated from a member of the genus *Cacospongia* [31]. Again, starting with a β-substituted furanyl moiety as a precursor for the structural related entities, they hypothesized a biosynthetic route based on several oxidation and rearrangement phenomena. This included the previously reported suggestions described above, to form out the different characters of lactones, substituted butenolides and lactams.

In the light of these hypotheses, it is logical to assume a similar potential biogenic route for the ircinianin members as the initial position in the considered sub-motif between these two terpenoid families is (bio)chemically equivalent. Furthermore, the proposed biosynthesis could plausibly yield ircinianin lactones B and C from **1**. Finally, the proposed biosynthetic pathway can also offer predictions about so far undetected class members (with identical molecular formulas to already published derivates).

### 2.2. Biological Evaluation

Marine natural products are well known as bioactive molecules of broad and unique chemical diversity [39]. The scaffold of ircinianin (**1**) and its analogues has been known for more than 30 years; however, a broader biological evaluation in the context of anti-infective agents was missing. The potential for bioactivity is illustrated by results of bioactivity screenings performed with crude extracts together with proven effects of the linear C_25_-terpenoides [5,7,11,40,41,42]. Inspired by evidence of cytotoxic effects of metabolites isolated from *Ircinia* sp., we screened the antitumor activity of **1** [10,20,22,43,44]. Finally, marine antifouling activity of **1** was also investigated to probe the ecological basis for production of these compounds by *Ircinia* sp. The prevention of surface colonization by sessile marine organisms is known as antifouling [45] and many sessile marine organisms produce potent antifouling compounds [46,47]. For example, the antifouling efficacy of natural and synthetic butenolides is well established and they have been used to generate protective coatings [48,49].

All tests were performed with **1** as a pre-screening for the scaffold’s activity within their respective assays. Based on the results from the pre-screening, the biological evaluation of the structural congeners was planned. Unfortunately, an instability phenomenon affected the purity of the isolated compounds. Due to the limited amounts of the isolated compounds, repurification would not have yielded the required sample quantity for the assays. This reality precluded biological evaluation of the structural congeners **3**–**8**.

#### 2.2.1. Antimicrobial Activity

The antibiotic effect of **1** was assessed against a set of clinically relevant bacteria (see Table S6) including representative strains from the “ESKAPE” panel and one mycobacterial strain. No activity was detected, up to the highest concentrations tested (MIC > 32 μg/mL).

#### 2.2.2. Antiviral Activity

**1** was tested against the Human Cytomegalovirus (HCMV) and the Severe Acute Respiratory Syndrome Corona Virus type 2 (SARS-CoV-2). Cells were pretreated with 1 or 10 µM ircinianin and then infected with HCMV or SARS-CoV-2 reporter viruses expressing fluorescent proteins as infection markers. At the time points indicated post infection, cells were monitored by automated fluorescence microscopy for cell counts and the number of infected cells (Figure S52). Under the conditions of the test, no inhibition was observed up to the highest concentrations tested (10 µM).

#### 2.2.3. Anthelmintic Activity

To investigate the activity against filarial nematodes, adult female worms of the rodent filarial nematode *Litomosoides sigmodontis* were cultured in vitro with a 0.1, 1 and 10 µM concentration of **1**. Motility of adult filariae as well as reduction of *Wolbachia* endosymbionts were assessed. No statistically significant reductions in *Wolbachia* levels (<32% *Wolbachia* reduction in comparison to media controls) or inhibition of worm motility (motility score of 1.7–1.8 vs. 2.0 in media controls) were observed.

#### 2.2.4. Antiprotozoal Activity

To assess antiprotozoal activity, **1** was tested in vitro against a panel composed of *P. falciparum*, *T. brucei rhodesiense*, *T. cruzi*, and *L. donovani* (see Table 2). **1** displayed a moderate activity against *P. falciparum* (IC_50_ 25.4 μM) and *L. donovani* (IC_50_ 16.6 μM). IC_50_ values were higher against *T. cruzi* and *T. brucei rhodesiense*, exceeding the 80 μM threshold considered of clinical relevance.

#### 2.2.5. Cytotoxicity

**1** displayed no activity in the National Cancer Institute’s (NCI) 60 cell-line cytotoxicity screen for anti-tumor agents and was also inactive against L6 (IC_50_ 150.1 μM) and HeLa cell line assays (IC_50_ > 64 μg/mL) (see Table S7).

#### 2.2.6. Marine Antifouling Activity

**1** was tested for inhibitory effects on marine larval settlement and metamorphosis against two model marine biofouling taxa, the Pacific transparent sea squirt *Ciona savignyi* and the blue mussel *Mytilus galloprovincialis*. In both cases, the compound was inactive up to the highest concentration tested (100 µg/mL).

#### 2.2.7. Conclusions of the Screening for Biological Activity of Ircinianin (**1**)

In summary, our screening efforts revealed a moderate activity against *P. falciparum* and *L. donovani* of ircinianin (**1**). This finding adds to the known glycine receptor modulatory activity of this compound class [13].

## 3. Materials and Methods

### 3.1. General Experimental Procedures

Optical rotation values were measured on a Jasco P-2000 polarimeter, using a 3.5 mm × 10 mm cylindrical quartz cell. Infrared spectra were obtained by employing a Jasco FTIR 4200 spectrometer, interfaced with a MIRacle ATR device (ZnSe crystal). CD spectra were recorded with a Jasco J-720 spectropolarimeter, using a quartz micro-cuvette with 2.0 mm path length. 1D and 2D NMR spectra were recorded on a 400 MHz Bruker AVANCE III NMR spectrometer operating at 400.17 and 100.63 MHz, respectively, which was equipped with a 5 mm broadband SmartProbe and an AVANCE III HD Nanobay console. All spectra were recorded in *d_4_*-MeOH (Deutero GmbH) at 298 K, the residual solvent signals (resonances at δ_H_ 3.31 and δ_C_ 49.15 ppm) were used as internal references. Trace impurities were assigned based on literature values [50]. ^1^H, ^13^C, DEPT 135, ^1^H-^13^C edited HSQC, ^1^H-^1^H-COSY, ^1^H-^1^H-TOCSY, ^1^H-^13^C HSQC-TOCSY, ^1^H-^13^C HMBC and ^1^H-^1^H-NOESY were recorded while utilizing Bruker standard pulse sequences. For processing, analyzing and data preparation, TopSpin 3.6.2 and MestReNova 12.0.4 were used. High-resolution mass spectra of the samples were run on an HRESI-TOF-MS Bruker maXis 4G mass spectrometer in positive mode to determine the accurate weight and molecular formula. The spectra were analyzed with Compass Data Analysis 4.4 (Bruker Daltonik, Bremen, Germany). For the isolation, a Waters HPLC system (operated by Millennium^32^ software), consisting of a Waters 1525 pump with an inline degasser, a Waters 996 photodiode array detector, and a Rheodyne 7725i injector was used. All solvents were used in HPLC or LC-MS grade (Sigma-Aldrich, Saint Louis, MO, USA).

### 3.2. Animal Material

The animal material (*Ircinia wistarii*) was collected by SCUBA diving from Wistari Reef, Great Barrier Reef, Australia in July 1998 from a depth of 20 m (see Figure S1). The sample was stored at −20 °C in EtOH until workup. A voucher specimen (voucher number HER 6, see Figure S2) is deposited in EtOH at the Pharmaceutical Institute, Department of Pharmaceutical Biology, University of Tübingen, Germany.

### 3.3. Extraction and Isolation

The sliced body of *Ircinia wistarii* (800 g wet weight) was extracted in 1:1 CHCl_3_:MeOH (1:1) and fractionated by preparative reversed phase open column chromatography with a MeOH-H_2_O gradient system (10% steps) and DCM. Eleven fractions were gained. For the detailed extraction and fractionation protocol, see Majer et al. [26]. Based on the analysis by HPLC-DAD and LC-MS the 50%, 70% and 90%-MeOH fraction were identified as containing ircinianin-like sesterterpenoids. These were selected for further purification using RP-HPLC: Phenomenex Luna Omega Polar C18, 5 µm, 100 Å column, 250 × 4.6 mm, at 1.2 mL/min and UV detection; 90% fraction was separated with a 3 min gradient elution, from 20:80 to 55:45 ACN/H_2_O + 0.1% TFA, followed by 27 min increasing the organic phase to 90:10 ratio; Phenomenex Kinetex EVO C18, 5 µm 100 Å column, 250 × 4.6 mm, at 1.2 mL/min and UV detection were used for the 50 and 100% fraction; for the 50% fraction: starting with 20:80 to 40:60 in 5 min ACN/H_2_O + 0.1% TFA, followed by 10% steps up to 60:40 in another 25 min; the 100% fraction was purified using a 10:90 mixture of ACN/H_2_O + 0.1% TFA for 3 min up to a gradient of 40:60, followed by 23 min reaching a ratio of 75:25. This afforded compounds **4** (2.0 mg) and **5** (1.8 mg) from the 50% fraction, compound **1** (140 mg) from the 90% and 100% fraction, and compounds **3** (2.3 mg), **6** (2.1 mg), **7** (2.5 mg) and **8** (2.4 mg) solely from the 100% fraction (see Figures S3, S5 and S22).

Ircinianin (**1**): White amorphous solid; HPLC-UV profile: see Figures S4 and S6; [α]$\begin{array}{c} 26\\ D \end{array}$ –97.6 (*c* = 0.328, MeOH), lit. [α]$\begin{array}{c} 20\\ D \end{array}$ –167 (*c* = 0.004, MeOH) [29]; IR (ATR) v_max_ 2928, 2870, 1714, 1654, 1456, 1308, 1200, 1143, 1023, 873, 801, 765, 723, 669, 599 cm^−1^, see Figure S7, lit. IR (KBr) v_max_ 3400–2600 br, 1709, 1659, 1620, 1261, 1119, 1022, 865, 751 cm^−1^ [23] and IR (KBr) v_max_ 3437, 1720, 1652, 1118 cm^−1^ [25], respectively; CD (MeOH): 241 nm (−84), 222 nm (+24), 204 nm (−110), see Figure S8, lit. CD: 241 nm (−22), 222 nm (+5.6), 203 (−30) [23]; HRESIMS at *m*/*z* 397.2373 [M + H]^+^ (calcd. for C_25_H_33_O_4_, 397.2373, see Figure S4); ^1^H and ^13^C NMR data, see Table 1.

Oxoircinianin (**3**): Yellow amorphous powder; HPLC-UV profile: see Figure S19; HRESIMS at *m*/*z* 413.2325 [M + H]^+^ (calcd. for C_25_H_33_O_5_, 413.2323); ^13^C NMR data, see Figure S21.

Ircinianin lactam A (**4**): White amorphous solid; HPLC-UV profile: see Figure S23; HRESIMS at *m*/*z* 470.2540 [M + H]^+^ (calcd. for C_27_H_36_NO_6_, 470.2537); ^1^H and ^13^C NMR data, see Table S1, Figures S24 and S25.

Oxoircinianin lactam A (**5**): Yellow oil; HPLC-UV profile: see Figure S26; HRESIMS at *m*/*z* 486.2486 [M + H]^+^ (calcd. for C_27_H_36_NO_7_, 486.2486); ^1^H and ^13^C NMR data, see Table S2, Figures S27 and S28.

Ircinianin lactone A (**6**): Yellow amorphous powder; HPLC-UV profile: see Figure S29 HRESIMS at *m*/*z* 413.2327 [M + H]^+^ (calcd. for C_25_H_33_O_5_, 413.2323); ^1^H and ^13^C NMR data, see Table S3, Figures S30 and S31.

Ircinianin lactone B (**7**): Yellow amorphous powder; HPLC-UV profile: see Figure S33; HRESIMS at *m*/*z* 443.2431 [M + H]^+^ (calcd. for C_26_H_35_O_6_, 443.2428, see Figure S33); ^1^H and ^13^C NMR data, see Table 1, Figures S34 and 35.

Ircinianin lactone C (**8**): Yellow amorphous powder; HPLC-UV profile: see Figure S44; HRESIMS at *m*/*z* 429.2281 [M + H]^+^ (calcd. for C_25_H_33_O_6_, 429.2272, see Figure S44); ^1^H and ^13^C NMR data, see Table 1, Figures S45 and S46.

### 3.4. Computational Methods

Both, the *R*- and the *S*-enantiomer of ircinianin lactone C (**8**), were adapted from the crystal structure of (2*S*,3′*S*,3a′*R*,5′*R*,7a′*R*)-5′-[(*E*)-5-(furan-3-yl)-2-methyl-pent-1-en-1-yl]-3-hydroxy-3′,4,7′-tri-methyl-1′,2′,3′,3a′,5′,7a′-hexa-hydro-5*H*-spiro-[furan-2,4′-inden]-5-one, as published in [26]. MOE (Molecular Operating Environment) 2018.0101 [51] was used for the generation of conformers using the Conformational Search tool. Employing a stochastic search algorithm, the potential energy surface was sampled by randomly perturbating the rotatable bonds followed by an energy minimization using the Merck Molecular Force Field (MMFF94) [52,53,54,55,56]. Newly found conformations were only kept within an energy limit of 20 kcal/mol above the detected global minimum and when the conformation was not too similar to any other found conformation (RMSD limit: 0.25). The search was terminated for both enantiomers by meeting the rejection limit of 200. This means that in 200 consecutive attempts, no new conformer was generated. Overall, a maximum iteration limit of 50,000 was used. Based on the moderate flexibility of the structure, these settings imply that a thorough conformational analysis was applied. For both the *R*- and the *S*-enantiomer of **8**, 30 distinct conformations were retrieved. Subsequently, these conformations were submitted to a free geometry optimization using B3-LYP/SV(P) [57,58,59] employing Grimme’s dispersion correction D3 [60] in TURBOMOLE 7.4.1 [61]. The dielectric constant was set to an epsilon of 32.7 for methanol at 25 °C. Extended Hückel theory (eht) was used for the initial guess of Mos. Scfconv was set to 8. All but one conformer converged eventually. This conformer was dismissed. Tetramethylsilane (TMS) was geometry optimized using the same method as described above. NMR shielding constants were calculated using mpshift [62] within TURBOMOLE. The resulting isotropic values for hydrogen and carbon in TMS were used to calculate the chemical shifts for all conformers of both enantiomers.

### 3.5. Biological Evaluation

#### 3.5.1. Antibacterial Assay

The minimal inhibitory concentration (MIC) was determined as described previously [63] in cation-adjusted Mueller–Hinton medium according to the standards and guidelines of the Clinical and Laboratory Standards Institute [64]. A 2-fold serial dilution of the test compound was prepared in microtiter plates and seeded with a final test bacterial inoculum 5 × 10^5^ colony-forming units (CFU)/mL. After an overnight incubation at 37 °C, the MIC was read as the lowest compound concentration preventing visible bacterial growth. The strain panel included representative species of nosocomial pathogens, known as “ESKAPE” bacteria. Specifically, the following strains were used: *Enterococcus faecium* BM 4147-1, *Staphylococcus aureus* ATCC 29213, *Klebsiella pneumoniae* ATCC 12657, *Acinetobacter baumannii* 09987, *Pseudomonas aeruginosa* ATCC 27853, and *Enterobacter aerogenes* ATCC 13048. *Bacillus subtilis* 168, *Escherichia coli* ATCC 25922, and *Mycobacterium smegmatis* mc^2^ 155 ATCC 700084 were used as further reference strains.

#### 3.5.2. Antiviral Assay

The antiviral activity of **1** was tested against the human cytomegalovirus (HCMV) and the severe acute respiratory syndrome coronavirus 2 (SARS CoV2), essentially as reported before [65,66].

*Activity against HCMV:* Human foreskin fibroblasts (HFF) (<25 passages) cultured in DMEM cell culture medium supplemented with 5% fetal bovine serum and 1% penicillin/streptomycin were seeded to a final cell concentration of 1 × 10^4^ cells per well. After 24 h of incubation at 37 °C, with 5% CO_2_ and 95% relative humidity and medium exchange, **1** was added at the concentrations of 1 and 10 µM. Infection was performed directly afterwards using HCMV TB40EdelUL16EGFP with MOIs of 0.3 and 1. 120 h post infection, the cells were fixed and permeabilized with 80% acetone in H_2_O at room temperature for 5 min. Intracellular staining for immediate early HCMV proteins was conducted by a 90 min incubation of 1:1000 IE1/2 HCMV antibody in PBS at 37 °C, a subsequent 45 min incubation of 1:2000 ALEXAFluor514 in PBS at 37 °C and a nuclear counterstaining with DAPI 1:20,000 in PBS for 8 min at room temperature. Each step was followed by a washing step, three times with PBS. Images were taken using the Cytation3 multiplate reader.

*Activity against SARS-CoV2:* CaCo2 cultured in DMEM cell culture medium supplemented with 10% fetal bovine serum, 1% penicillin/streptomycin and 1% non-essential amino acids were seeded to a final cell concentration of 1 × 10^4^ cells per well. After 24 h of incubation at 37 °C, with 5% CO_2_ and 95% relative humidity and medium exchange, **1** was added at the concentrations of 1 and 10 µM. Infection was performed directly afterwards using SARS-CoV2 mNeonGreen at 1:2000 dilution. 48 h post infection, the supernatant was taken to reinfect another plate of Caco2 for evaluation of viral supernatant as a sign for active replication of the virus. The initially infected cells were fixed with 2% paraformaldehyde in PBS and stained with Hoechst 33342 at 37 °C for 10 min. Afterwards, the cells were washed with PBS 3 times. 48 h post infection, the reinfected cells were fixed and stained as described above. Images were taken using the Cytation3 multiplate reader.

#### 3.5.3. Anthelmintic Assay

*L. sigmodontis* adult female worms were isolated from chronically infected cotton rats (UKB). Three *L. sigmodontis* female adult worms per condition were cultured in supplemented Minimal Essential Medium (MEM) on a LLCMK2 cell layer, as previously described [67]. The positive controls consisted of doxycycline at 40, 20, 10 and 5 µM. Candidate **1** was tested at concentrations of 0.1, 1 and 10 µM. Filarial viability was assessed by determining the mean worm motility scores on a scale of 0 (immotile) to 2 (maximum motility) before drug addition and at 1 h, 1 day, 2 day, 4 day, 6 day, 8 day, 10 day, 12 day and 14 day post drug addition. The drug-containing media was renewed every other day. Depletion of *Wolbachia* endosymbionts from female adult worms was analyzed after two weeks of in vitro culture and determined as previously described [68] using primers for the single copy gene *Wolbachia ftsZ*.

#### 3.5.4. Antiprotozoal Assays

*Activity against Trypanosoma brucei rhodesiense STIB900.* The stock was isolated in 1982 from a human patient in Tanzania, and after several mouse passages, cloned and adapted to axenic culture conditions [69]. Minimum Essential Medium (50 µL) supplemented with 25 mM HEPES, 1 g/L additional glucose, 1% MEM non-essential amino acids (100×), 0.2 mM 2-mercaptoethanol, 1 mM Na-pyruvate and 15% heat inactivated horse serum was added to each well of a 96-well microtiter plate. Serial drug dilutions ranging from 100 to 0.002 μg/mL were prepared. Then, 4 × 10^3^ bloodstream forms of *T. b. rhodesiense* STIB 900 in 50 µL were added to each well and the plate was incubated at 37 °C under a 5% CO_2_ atmosphere for 70 h. 10 µL of a resazurin solution (resazurin, 12.5 mg in 100 mL double-distilled water) was then added to each well and incubation continued for a further 2–4 h [70]. The plates were read with a Spectramax Gemini XS microplate fluorometer (Molecular Devices Cooperation, Sunnyvale, CA, USA) using an excitation wavelength of 536 nm and an emission wavelength of 588 nm. Data were analyzed using Softmax Pro (Molecular Devices Cooperation, Sunnyvale, CA, USA), which calculated IC_50_ values by linear regression [71] and 4-parameter logistic regression from the sigmoidal dose inhibition curves. Melarsoprol (Arsobal Sanofi-Aventis, received from WHO) was used as control (IC_50_ 0.013 ± 0.006 μM).

*Activity against T. cruzi.* Rat skeletal myoblasts (L-6 cells) were seeded in 96-well microtiter plates at 2000 cells/well in 100 μL RPMI 1640 medium with 10% FBS and 2 mM L-glutamine. After 24 h, the media in each well was removed and replaced with 100 μL of fresh media containing 5000 trypomastigote forms of *T. cruzi* Tulahuen strain C2C4 containing the β-galactosidase (*lacZ*) gene [72]. After 48 h, the media was removed from the wells again, and this time, replaced by 100 μL fresh medium with or without **1** from 100 to 0.002 μg/mL. After 96 h of incubation, the plates were inspected under an inverted microscope to assess growth and sterility of the controls. Then, the substrate CPRG/Nonidet (50 μL) was added to all wells. A color reaction developed within 2–6 h and could be read photometrically at 540 nm. Data were analyzed with the graphic program Softmax Pro (Molecular Devices), which calculated IC_50_ values by linear regression [71] and 4-parameter logistic regression from the sigmoidal dose inhibition curves. Benznidazole (received from DNDi, synthesized by Epichem) was used as control (IC_50_ 2.31 ± 1.15 μM).

*Activity against L. donovani axenic amastigotes.* Amastigotes of *L. donovani* strain MHOM/ET/67/L82 were grown in axenic culture at 37 °C in SM medium [73] at pH 5.4 supplemented with 10% heat-inactivated fetal bovine serum under an atmosphere of 5% CO_2_ in air. 100 µL of culture medium with 10^5^ amastigotes from axenic culture with or without a serial drug dilution were seeded in 96-well microtiter plates. Serial drug dilutions ranged from 100 to 0.002 μg/mL. After 70 h of incubation, the plates were inspected under an inverted microscope to assure growth of the controls and sterile conditions. 10 μL of resazurin (12.5 mg resazurin dissolved in 100 mL distilled water) were then added to each well and the plates were incubated for another 2 h. Then, the plates were read with a Spectramax Gemini XS microplate fluorometer (Molecular Devices Cooperation, Sunnyvale, CA, USA) using an excitation wavelength of 536 nm and an emission wavelength of 588 nm. From the sigmoidal inhibition curves the IC_50_ values were calculated by linear regression [71] and 4-parameter logistic regression using SoftmaxPro software (Molecular Devices Cooperation, Sunnyvale, CA, USA). Miltefosine (Sigma, Saint Louis, MO, USA) was used as control (IC_50_ 0.65 ± 0.27 μM)

*Activity against P. falciparum*. In vitro activity against erythrocytic stages of *P. falciparum* was determined using a ^3^H-hypoxanthine incorporation assay [74,75] against the drug-sensitive NF54 strain [76]. Compounds were dissolved in DMSO at 10 mg/mL and further diluted in medium before being added to parasite cultures incubated in RPMI 1640 medium without hypoxanthine, supplemented with HEPES (5.94 g/L), NaHCO_3_ (2.1 g/L), neomycin (100 U/mL), Albumax^R^ (5 g/L) and washed human red cells A^+^ at 2.5% haematocrit (0.3% parasitaemia). Serial drug dilutions ranged from 100 to 0.002 μg/mL. The 96-well plates were incubated in a humidified atmosphere at 37 °C; 4% CO_2_, 3% O_2_, 93% N_2_. After 48 h 50 μL of ^3^H-hypoxanthine (=0.5 μCi) was added to each well of the plate. The plates were incubated for a further 24 h under the same conditions. The plates were then harvested with a Betaplate™ cell harvester (Wallac, Zurich, Switzerland) and the red blood cells were transferred onto a glass fiber filter then washed with distilled water. The dried filters were inserted into a plastic foil with 10 mL of scintillation fluid and counted in a Betaplate™ liquid scintillation counter (Wallac, Zurich, Switzerland). IC_50_ values were calculated from sigmoidal inhibition curves by linear regression [71] using Microsoft Excel. Chloroquine diphosphate (Sigma C6628) was used as control (IC_50_ 0.006 ± 0.002 μM).

#### 3.5.5. Cytotoxicity Assays

*One dose NCI-60 panel:* **1** was selected for the anticancer drug screening service as a part of the Developmental Therapeutics Program at the National Cancer Institute (NCI). In vitro tumor growth inhibitory effects were explored using a standard protocol with a single high dose test against a panel comprising 60 human cancer cell lines [77,78]. It is noteworthy to mention that we had to remove the results of the NCI-H23 cell line, since NCI informed us that its cell line’s identity was not authenticated during the time frame in which the screening of **1** was conducted.

*In vitro cytotoxicity assay with HeLa cells*: The cytotoxicity test against the HeLa human cervical carcinoma cell line was performed as described previously [63] in RPMI cell culture medium supplemented with 10% fetal bovine serum using the 7-hydroxy-3H-phenoxazin-3-one-10-oxide (resazurin) assay. Briefly, a 2-fold serial dilution of the test compounds was prepared in duplicates in a microtiter plate and seeded with trypsinized HeLa cells to a final cell concentration of 1 × 10^4^ cells per well. After 24 h of incubation at 37 °C, with 5% CO_2_ and 95% relative humidity, resazurin was added at a final concentration of 200 μM, and cells were again incubated overnight. Cell viability was assessed by determining the reduction of resazurin to the fluorescent resorufin. Fluorescence was measured in a TECAN Infinite M200 reader at an excitation wavelength of 560 nm and an emission wavelength of 600 nm in relation to an untreated control.

*In vitro cytotoxicity assay with L-6 cells.* Assays were performed in 96-well microtiter plates, each well containing 100 μL of RPMI 1640 medium supplemented with 1% L-glutamine (200 mM) and 10% fetal bovine serum, and 4000 L-6 cells (a primary cell line derived from rat skeletal myoblasts) [79,80]. Serial drug dilutions ranged from 100 to 0.002 μg/mL. After 70 h of incubation, the plates were inspected under an inverted microscope to assure growth of the controls and sterile conditions. 10 μL of resazurin was then added to each well and the plates were incubated for another 2 h. Then, the plates were read with a Spectramax Gemini XS microplate fluorometer (Molecular Devices Cooperation, Sunnyvale, CA, USA) using an excitation wavelength of 536 nm and an emission wavelength of 588 nm. The IC_50_ values were calculated by linear regression [71] and 4-parameter logistic regression from the sigmoidal dose inhibition curves using SoftmaxPro software (Molecular Devices Cooperation, Sunnyvale, CA, USA). Podophyllotoxin (Sigma P4405) was used as control (IC_50_ 0.011 ± 0.005 μM).

#### 3.5.6. Marine Antifouling Assays

Antifouling activity was assessed via inhibition of settlement and metamorphosis of larvae of the Pacific transparent sea squirt (*Ciona savignyi*) and the blue mussel (*Mytilus galloprovincialis*). Methods followed those published by Grant et al. [81]. Adults of both species were collected from coastal populations in the Nelson region of New Zealand, held in a recirculating seawater system (18 ± 1 °C, 33 ± 1 PSU) and fed bulk-cultured *Isochorysis galbana* until ready to spawn. Larval spawning and rearing procedures followed previously described methods for *C. savignyi* [82] and *M. gallorprovincialis* [83]. Competent larvae were diluted in artificial seawater to yield 3 ± 1 larvae/mL. Aliquots of these larval suspensions were added to 12-well tissue culture plates (Corning Co-Star) containing serial dilutions of **1** ranging from 0.1–100 µg/mL. Controls were included, and three replicates were performed in all cases. After 5 days of incubation at 18 ± 1 °C, the number of successfully settled and metamorphosed individuals were counted in each well. Sigmoidal dose-response relationships were explored using R Statistical Software [84] to determine whether inhibition had occurred relative to the controls.

## Figures and Tables

**Table 1:** ^1^H (400 MHz) and ^13^C (100 MHz) data for ircinianin (**1**), ircinianin lactone B (**7**) and ircinianin lactone C (**8**), recorded in *d*_4_-MeOH at 298 K. Chemical shifts are given in ppm.

	*Irciniacin* (1)		*Ircinianin lactone B* (7)		*Ircinianin lactone C* (8)	
Position	*δ*_H_, Mult. (*J* in Hz)	*δ*_C_, Type ^A^	*δ*_H_, Mult. (*J* in Hz)	*δ*_C_, Type ^A^	*δ*_H_, Mult. (*J* in Hz)	*δ*_C_, Type ^A^
1	7.38, t (1.7)	144.0, CH	5.84, br q (1.2)	104.4, CH	6.02, d (2.3)	101.1, CH
2	6.30, d (0.9)	112.1, CH	6.98, br quin (1.2)	144.65, CH 144.72 ^E^	-	172.7, C
3	-	126.5, C	-	139.03, C 139.08 ^E^	5.90, d (2.3)	117.9, CH
4	7.26, m	140.3, CH	-	173.5, C	-	173.8, C
5	2.41, br t (7.5) ^F^	25.3, CH_2_	2.26, br t (7.8)	25.5, CH_2_	2.43, m ^F^	28.2, CH_2_
6	1.68, m *	29.5, CH_2_	1.67, m *	26.44, CH_2_ 26.47 ^E^	1.76, m *	25.9, CH_2_
7	2.04, m	40.5, CH_2_	2.07, dd (7.9, 7.2)	40.2, CH_2_	2.11, m	40.4, CH_2_
8	-	136.6, C	-	135.75, C 135.78 ^E^	-	135.9, C
9	1.57, d (1.3)	16.3, CH_3_	1.57, d (1.3)	16.06, CH_3_ 16.08 ^E^	1.59, d	16.2, CH_3_
10	5.11, dd (10.3, 1.1)	125.0, CH	5.12, m	125.41, CH 125.44 ^E^	5.15, d (10.3)	125.7, CH
11	3.08, dm (10.3)	48.7, CH ^C^	3.06, dm (10.3)	48.6, CH ^C^	3.08, dm (10.1)	48.7, CH ^C^
12	5.03, m	123.6, CH	5.01, m	123.3, CH	5.03, m	123.4, CH
13	-	137.1, C	-	137.0, C	-	137.2, C
14	1.71, m	20.8, CH_3_^D^	1.70, d (1.4)	20.56, CH_3_^B^	1.71, m	20.68, CH_3_^D^
15	2.42, m ^F^	46.2, CH	2.40, m	46.0, CH	2.42, m ^F^	46.2, CH
16	a. 1.89, m b. 1.34, m ^G^	27.3, CH_2_	a. 1.89, m b. 1.32, m ^F^	27.1, CH_2_	a. 1.89, m b. 1.34, m ^G^	27.3, CH_2_
17	a. 2.00, m b. 1.32, m ^G^	33.6, CH_2_	a. 2.00, m b. 1.29, m ^F^	33.4, CH_2_	a. 2.02, m b. 1.31, m ^G^	33.6, CH_2_
18	1.65, m	33.2, CH	1.63, m *	33.1, CH	1.64, m *	33.2, CH
19	0.92, d (6.3)	20.7, CH_3_^D^	0.92, d (6.2)	20.62, CH_3_^D^	0.93, d (6.2)	20.74, CH_3_^D^
20	1.61, m *	52.0, CH	1.61, m *	51.7, CH	1.62, m *	51.8, CH
21	-	86.9, C	-	86.7, C	-	86.8, C
22	-	179.2, C ^B^	-	179.3, C ^B^	-	179.1, C ^B^
23	-	97.5, C	-	97.3, C	-	97.5, C
24	-	177.7, C ^B^	-	177.6, C ^B^	-	177.7, C ^B^
25	1.64, br s	6.1, CH_3_	1.63, br s	6.0, CH_3_	1.65, br s	6.1, CH_3_
1′			3.52, br s	57.13, CH_3_ 57.16 ^E^		

^A^ Multiplicities were deduced from DEPT135 and multiplicity-edited ^1^H-^13^C HSQC NMR experiments. ^B/D^ Assignments within a column may be interchanged. ^C^ Overlapped by solvent peak. ^E^ A second signal was observed at a ratio of ~1:1 attributed to epimerization at C-1 of the γ-methoxy-γ-butenolide moiety. ^F/G^ Overlapping signals within a column. * Overlapped by other signals.

**Table 2:** In vitro antiprotozoal activity of **1**. All IC_50_ values are given in μM and are the means of two independent assays; the individual values vary by a factor of less than 2.

Substance	*P. falciparum*	*T. brucei rhodesiense*	*T. cruzi*	*L. donovani*
Ircinianin (**1**)	25.4	82.8	190.9	16.6
positive controls	0.006 ^a^	0.020 ^b^	3.36 ^c^	0.486 ^d^

^a^ chloroquine; ^b^ melarsoprol; ^c^ benznidazole; ^d^ miltefosine.

## Data Availability

Not applicable.

## Supplementary Materials

The following are available online at https://www.mdpi.com/article/10.3390/md20080532/s1, Figures S1 and S2: photographs of the investigated organism. Tables S1–S3 and Figures S3–S53: spectral data of compounds **1** and **3**–**8**. Tables S4 and S5 and Figure S54: bioassay data for compound **1**. Table S6. Antimicrobial assays for ircinianin (1). Table S7. Cytotoxocity Assays.
