# Supplementary material for: Discovery of Ircinianin Lactones B and C—Two New Cyclic Sesterterpenes from the Marine Sponge Ircinia wistarii
Source: Mar Drugs. 2022 Aug 19;20(8):532. doi: 10.3390/md20080532 (PMC9410537; doi:10.3390/md20080532)
Supplement: Supplementary file 1 [file marinedrugs-20-00532-s001.zip › marinedrugs-1793677-supplementary.pdf]

Supporting Information

Discovery of Ircinianin Lactones B and C – Two New Cyclic Sesterterpenes from the Marine Sponge *Ircinia wistarii*

Thomas Majer [1], Keshab Bhattarai [1], Jan Straetener [2], Justus Pohlmann [3], Patrick Cahill [4], Markus O. Zimmermann [5], Marc P. Hübner [6,7], Marcel Kaiser [8,9], Johan Svenson [4], Michael Schindler [3], Heike Brötz-Oesterhelt [2,10,11] Frank M. Boeckler [5] and Harald Gross [1,10,11]*

1 Department of Pharmaceutical Biology, Pharmaceutical Institute, University of Tübingen, Auf der Morgenstelle 8, 72076 Tübingen, Germany; thomas.majer@uni-tuebingen.de (T.M.); keshab.bhattarai@uni-tuebingen.de (K.B.); harald.gross@uni-tuebingen.de (H.G.)
2 Department of Microbial Bioactive Compounds, Interfaculty Institute of Microbiology and Infection Medicine (IMIT), University of Tübingen, Auf der Morgenstelle 28, 72076 Tübingen, Germany; jan.straetener@uni-tuebingen.de (J.St.); heike.broetz-oesterhelt@uni-tuebingen.de (H.B.-O.)
3 Institute for Medical Virology and Epidemiology, Section Molecular Virology, University Hospital Tübingen, 72076 Tübingen, Germany; justus.pohlmann@med.uni-tuebingen.de (J.P.); michael.Schindler@med.uni-tuebingen.de (M.S.)
4 Cawthron Institute, 98 Halifax Street East, Nelson 7010, New Zealand; johan.svenson@cawthron.org.nz (J.Sv.); patrick.cahill@cawthron.org.nz (P.C.)
5 Lab for Molecular Design and Pharmaceutical Biophysics, Department of Pharmacy and Biochemistry, Institute of Pharmaceutical Sciences, University of Tübingen, Auf der Morgenstelle 8, 72076 Tübingen, Germany; m.zimmermann@uni-tuebingen.de (M.O.Z.); frank.boeckler@uni-tuebingen.de (F.M.B.)
6 Institute for Medical Microbiology, Immunology and Parasitology, University Hospital Bonn, 53127 Bonn, Germany; huebner@uni-bonn.de (M.P.H.)
7 German Center for Infection Research (DZIF), partner site Bonn-Cologne, Bonn, Germany
8 Swiss Tropical and Public Health Institute, Kreuzstrasse 2, 4123 Allschwil, Switzerland; marcel.kaiser@swisstph.ch (M.K.)
9 Faculty of Science, University of Basel, Petersplatz 1, 4002 Basel, Switzerland
10 Cluster of Excellence ´Controlling Microbes to Fight Infections´, University of Tübingen, 72076 Tübingen, Germany
11 German Center for Infection Research (DZIF), partner site Tübingen, Tübingen, Germany

CONTENT

Animal Material
Figure S1 *Ircinia wistarii* underwater photograph
Figure S2 *Ircinia wistarii* voucher sample

Spectral data for ircinianin (1)
Figure S3 Chromatogram of the 90%-LC fraction
Figure S4 HR-MS spectrum, predicted formula, extracted UV profile of **1** (90% fraction)
Figure S5 Chromatogram of the 100%-LC fraction
Figure S6 HR-MS spectrum, predicted formula, extracted UV profile of **1** (100% fraction)
Figure S7 FT-IR spectrum of **1**
Figure S8 CD-spectrum of **1** in MeOH
Figure S9 *De-novo* structure elucidation of **1** and selected 2D-NMR correlations
Figure S10 Comparison of ^{1}H NMR spectra: **1** from 90% fraction vs. **1** from 100% fraction
Figure S11 ^{1}H NMR spectrum of **1** in d_4-MeOH
Figure S12 ^{13}C NMR spectrum of **1** in d_4-MeOH
Figure S13 DEPT135 NMR spectrum of **1** in d_4-MeOH
Figure S14 multiplicity edited ^{1}H-^{13}C HSQC NMR spectrum of **1** in d_4-MeOH
Figure S15 ^{1}H-^{1}H COSY NMR spectrum of **1** in d_4-MeOH
Figure S16 ^{1}H-^{1}H TOCSY NMR spectrum of **1** in d_4-MeOH
Figure S17 ^{1}H-^{13}C HMBC NMR spectrum of **1** in d_4-MeOH
Figure S18 ^{1}H-^{1}H NOESY NMR spectrum of **1** in d_4-MeOH

Spectral data for oxoircinianin (3)
Figure S19 HR-MS spectrum, predicted formula and extracted UV profile of **3**
Figure S20 Comparison of the MS^{2} spectra of **1**, **3** and **5**
Figure S21 Comparison of the ^{13}C NMR spectra of **3** and **5**

Spectral data for ircinianin lactam A (4)
Figure S22 Chromatogram of the 50%-LC fraction
Figure S23 HR-MS spectrum, predicted formula and extracted UV profile of **4**
Table S1 ^{1}H and ^{13}C NMR data of **4** in d_4-MeOH
Figure S24 ^{1}H NMR spectrum of **4** in d_4-MeOH
Figure S25 ^{13}C NMR spectrum of **4** in d_4-MeOH

Spectral data for oxoircinianin lactam A (5)
Figure S26 HR-MS spectrum, predicted formula and extracted UV profile of **5**
Table S2 ^{1}H and ^{13}C NMR data of **5** in d_4-MeOH
Figure S27 ^{1}H NMR spectrum of **5** in d_4-MeOH
Figure S28 ^{13}C NMR spectrum of **5** in d_4-MeOH

Spectral data for ircinianin lactone A (6)
Figure S29 HR-MS spectrum, predicted formula and extracted UV profile of **6**
Table S3 ^{1}H and ^{13}C NMR data of **6** in d_4-MeOH
Figure S30 ^{1}H NMR spectrum of **6** in d_4-MeOH
Figure S31 ^{13}C NMR spectrum of **6** in d_4-MeOH
Figure S32 multiplicity edited ^{1}H-^{13}C HSQC NMR spectrum of **6** in d_4-MeOH

Spectral data for ircinianin lactone B (7)

Figure S33 HR-MS spectrum, predicted formula and extracted UV profile of **7**
Table S4 Comparison of chemical shift values of **6** and **7**
Figure S34 ^{1}H NMR spectrum of **7** in d_4-MeOH
Figure S35 ^{13}C NMR spectrum of **7** in d_4-MeOH
Figure S36 DEPT135 NMR spectrum of **7** in d_4-MeOH
Figure S37 ^{1}H NMR spectrum of **7** in d_4-MeOH (before 2D experiments)
Figure S38 multiplicity edited ^{1}H-^{13}C HSQC NMR spectrum of **7** in d_4-MeOH
Figure S39 ^{1}H-^{1}H COSY NMR spectrum of **7** in d_4-MeOH
Figure S40 ^{1}H-^{13}C HSQC-TOCSY NMR spectrum of **7** in d_4-MeOH
Figure S41 ^{1}H-^{13}C HMBC NMR spectrum of **7** in d_4-MeOH
Figure S42 ^{1}H-^{1}H NOESY NMR spectrum of **7** in d_4-MeOH
Figure S43 Interpretation and visualization of the NOESY data of **7**

Spectral data for ircinianin lactone C (8)

Figure S44 HR-MS spectrum, predicted formula and extracted UV profile of **8**
Table S5 Comparison of chemical shift values of **1** and **8**
Figure S45 ^{1}H NMR spectrum of **8** in d_4-MeOH
Figure S46 ^{13}C NMR spectrum of **8** in d_4-MeOH
Figure S47 DEPT135 NMR spectrum of **8** in d_4-MeOH
Figure S48 ^{1}H NMR spectrum of **8** in d_4-MeOH (before 2D experiments)
Figure S49 multiplicity edited ^{1}H-^{13}C HSQC NMR spectrum of **8** in d_4-MeOH
Figure S50 ^{1}H-^{1}H COSY NMR spectrum of **8** in d_4-MeOH
Figure S51 ^{1}H-^{13}H HSQC-TOCSY NMR spectrum of **8** in d_4-MeOH
Figure S52 ^{1}H-^{13}C HMBC NMR spectrum of **8** in d_4-MeOH
Figure S53 ^{1}H-^{1}H NOESY NMR spectrum of **8** in d_4-MeOH

Biological assay data for ircinianin (1)

Table S6 Results of the antimicrobial assays
Figure S54 Results of the antiviral assays
Table S7 Results of the cytotoxicity assays

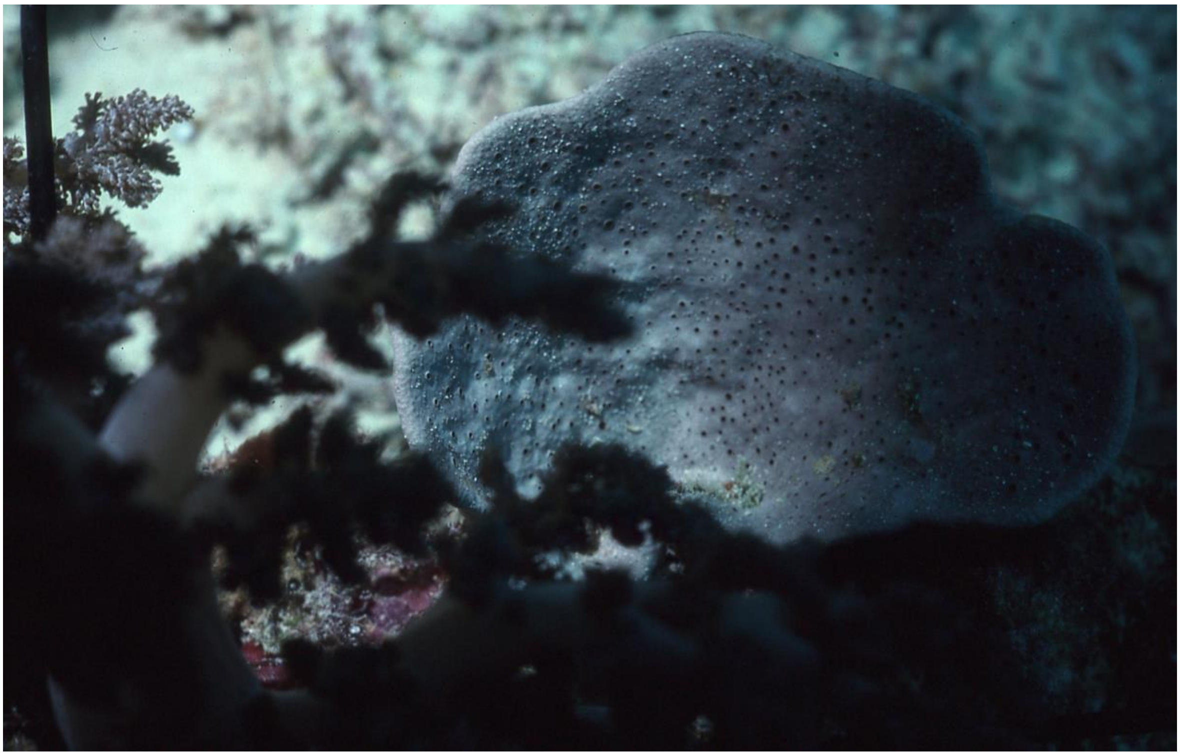

Figure S1. Underwater photograph of *Ircinia wistarii* (HER 6).

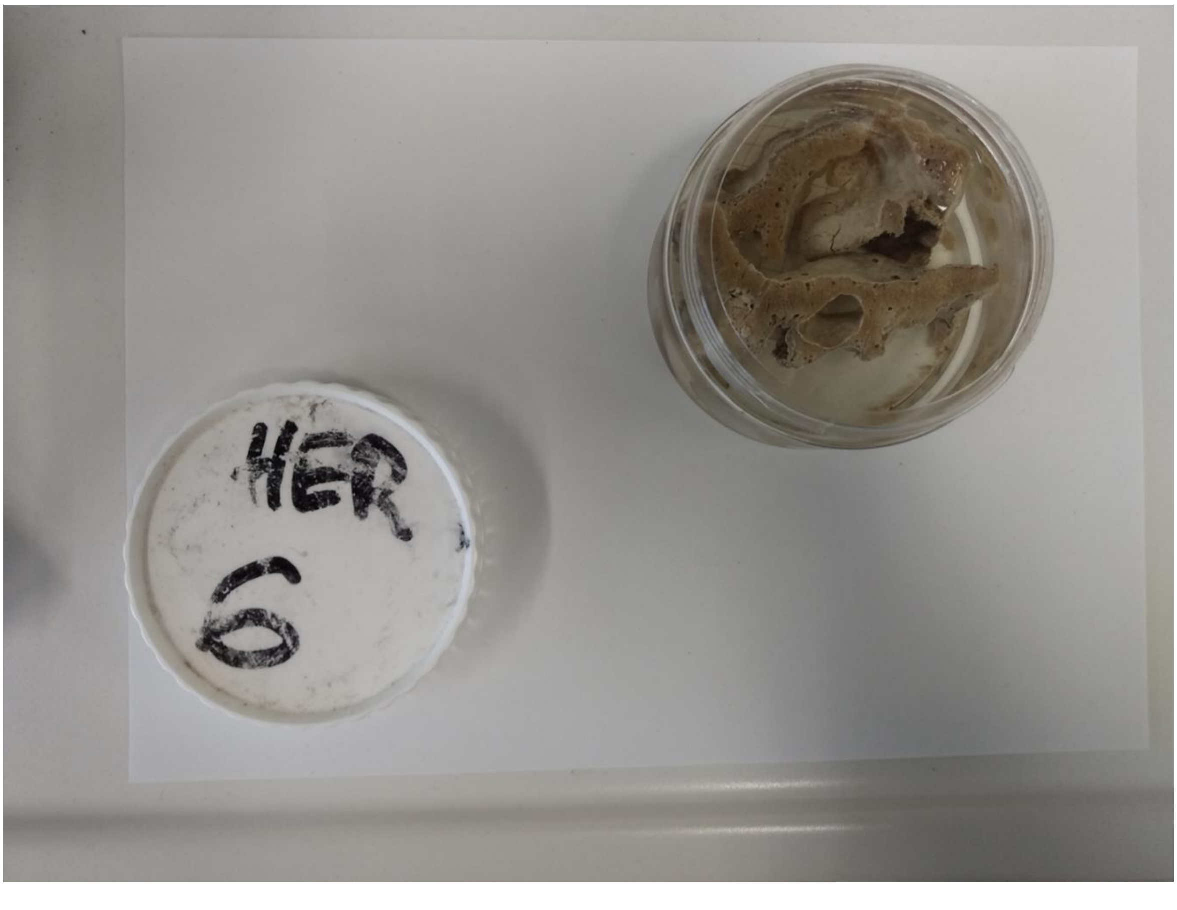

Figure S2. *Ircinia wistarii* (HER 6) – voucher sample.

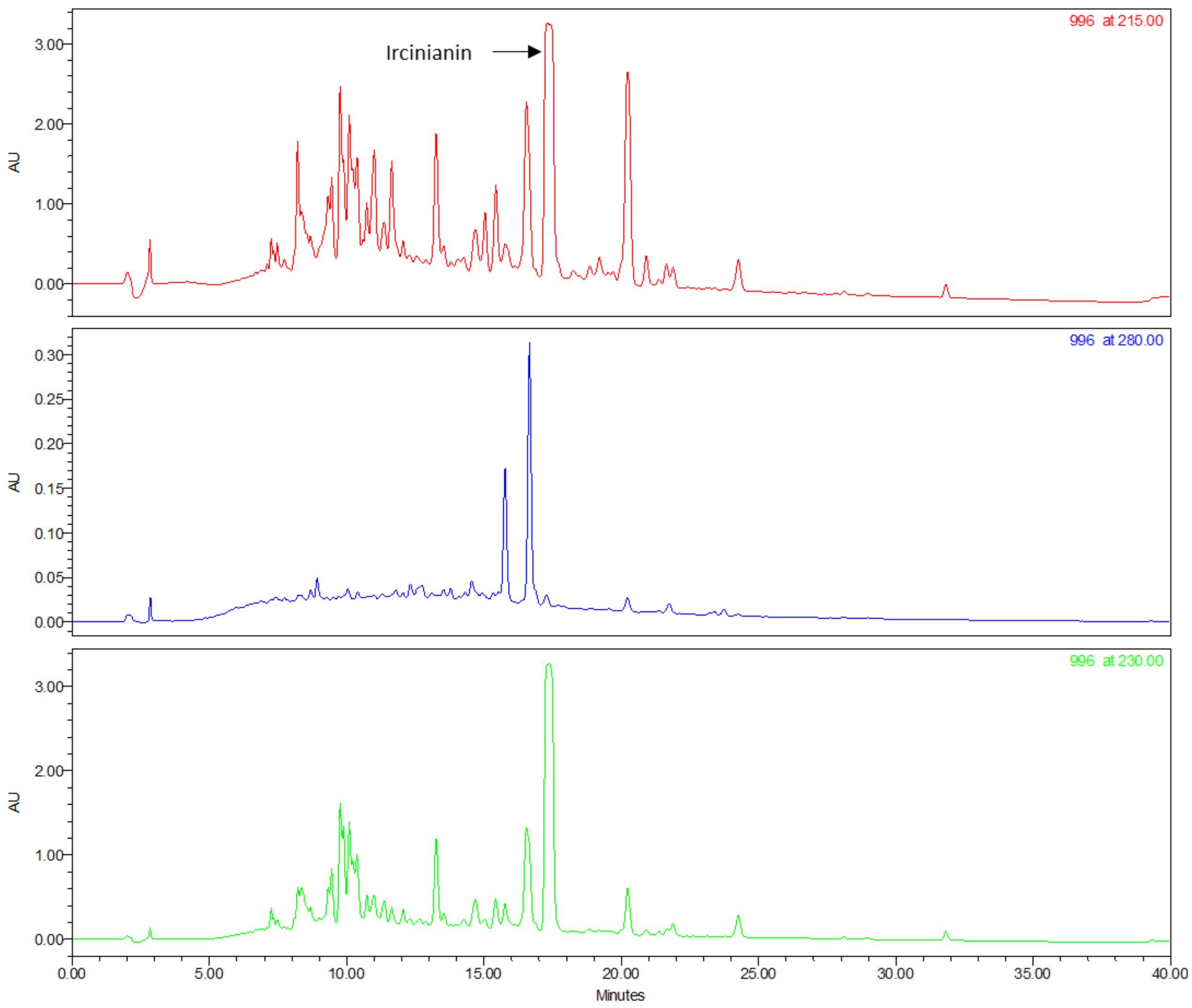

Figure S3. Chromatogram of the 90%-LC-fraction.

A

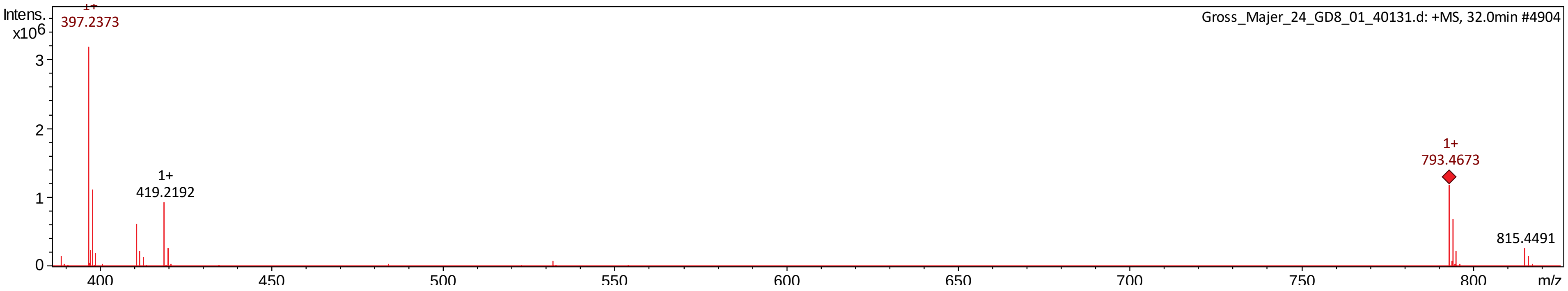

B

Meas. m/z	#	Ion Formula	m/z	err [ppm]	Mean err [ppm]	rdb	N-Rule	e‾ Conf	mSigma	Std I
397.2373	1	$C_{26}H_{29}N_4$	397.2387	3.5	3.1	15.0	ok	even	29.7	43.4
397.2373	2	$C_{25}H_{33}O_4$	397.2373	0.2	0.0	10.0	ok	even	42.6	61.2
397.2373	3	$C_{21}H_{29}N_6O_2$	397.2347	-6.6	-7.5	11.0	ok	even	56.6	84.5
397.2373	4	$C_{20}H_{33}N_2O_6$	397.2333	-10.0	-10.6	6.0	ok	even	69.5	103.4
397.2373	5	$C_{14}H_{33}N_6O_7$	397.2405	8.2	6.8	2.0	ok	even	98.7	153.5
397.2373	6	$C_{11}H_{25}N_{16}O$	397.2392	4.8	1.6	8.0	ok	even	99.6	159.5
397.2373	7	$C_{10}H_{29}N_{12}O_5$	397.2378	1.4	-1.1	3.0	ok	even	112.3	180.1

C

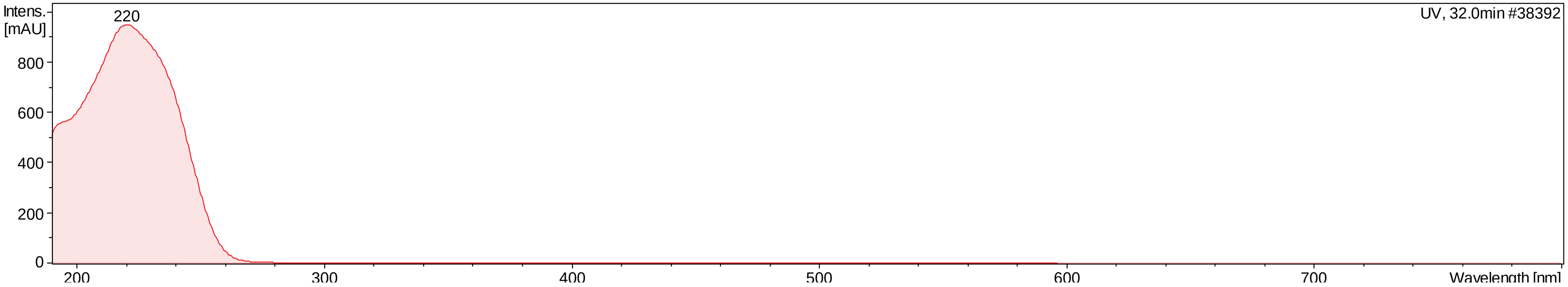

Figure S4. Ircinianin (**1**, 90%-fraction) Analysis – A: HRMS-result; B: predicted molecular formula; C: extracted UV-Profile.

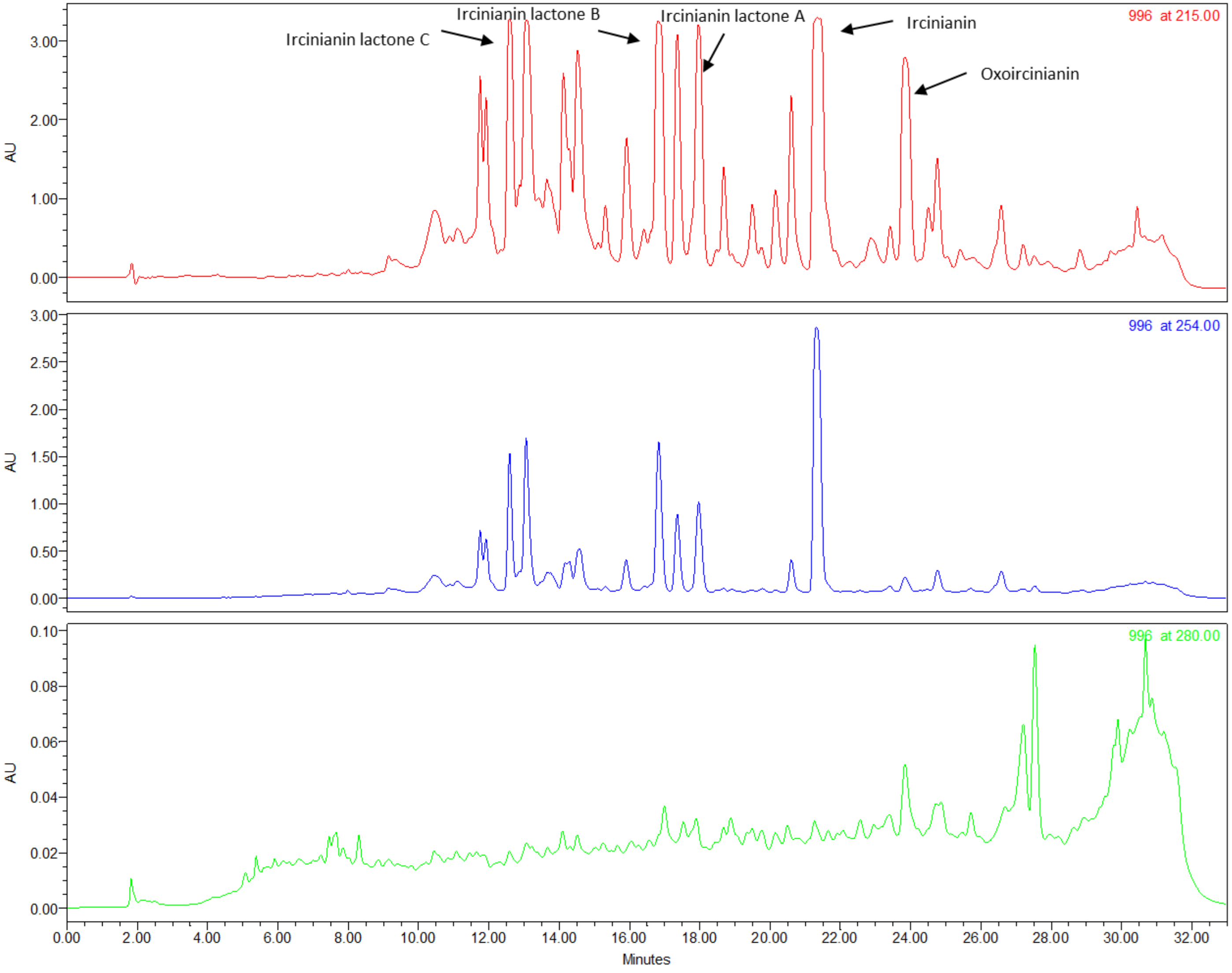

Figure S5. Chromatogram of the 100%-LC-fraction.

A

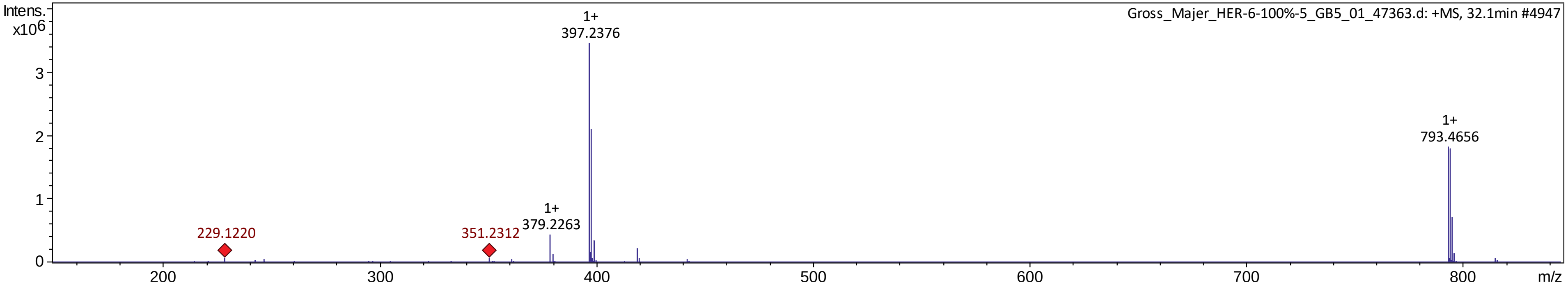

B

Meas. m/z	#	Ion Formula	m/z	err [ppm]	Mean err [ppm]	rdb	N-Rule	e⁻ Conf	mSigma	Std I
397.2376	1	$C_{26}H_{29}N_4$	397.2387	2.6	3.2	15.0	ok	even	180.9	209.1
397.2376	2	$C_{25}H_{33}O_4$	397.2373	-0.7	0.1	10.0	ok	even	194.0	227.3
397.2376	3	$C_{21}H_{29}N_6O_2$	397.2347	-7.5	-7.4	11.0	ok	even	207.9	250.4
397.2376	4	$C_{14}H_{33}N_6O_7$	397.2405	7.3	6.9	2.0	ok	even	250.1	319.6
397.2376	5	$C_{11}H_{25}N_{16}O$	397.2392	3.9	1.6	8.0	ok	even	250.8	325.4
397.2376	6	$C_{10}H_{29}N_{12}O_5$	397.2378	0.5	-1.1	3.0	ok	even	263.7	346.2

C

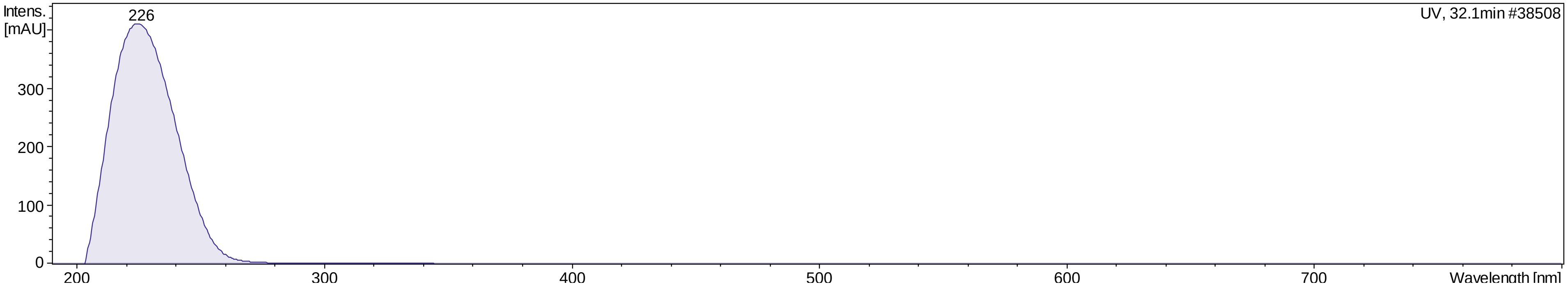

Figure S6. Ircinianin (**1**, 100%-fraction) MS-Analysis – A: HRMS-result; B: predicted molecular formula; C: extracted UV-Profile.

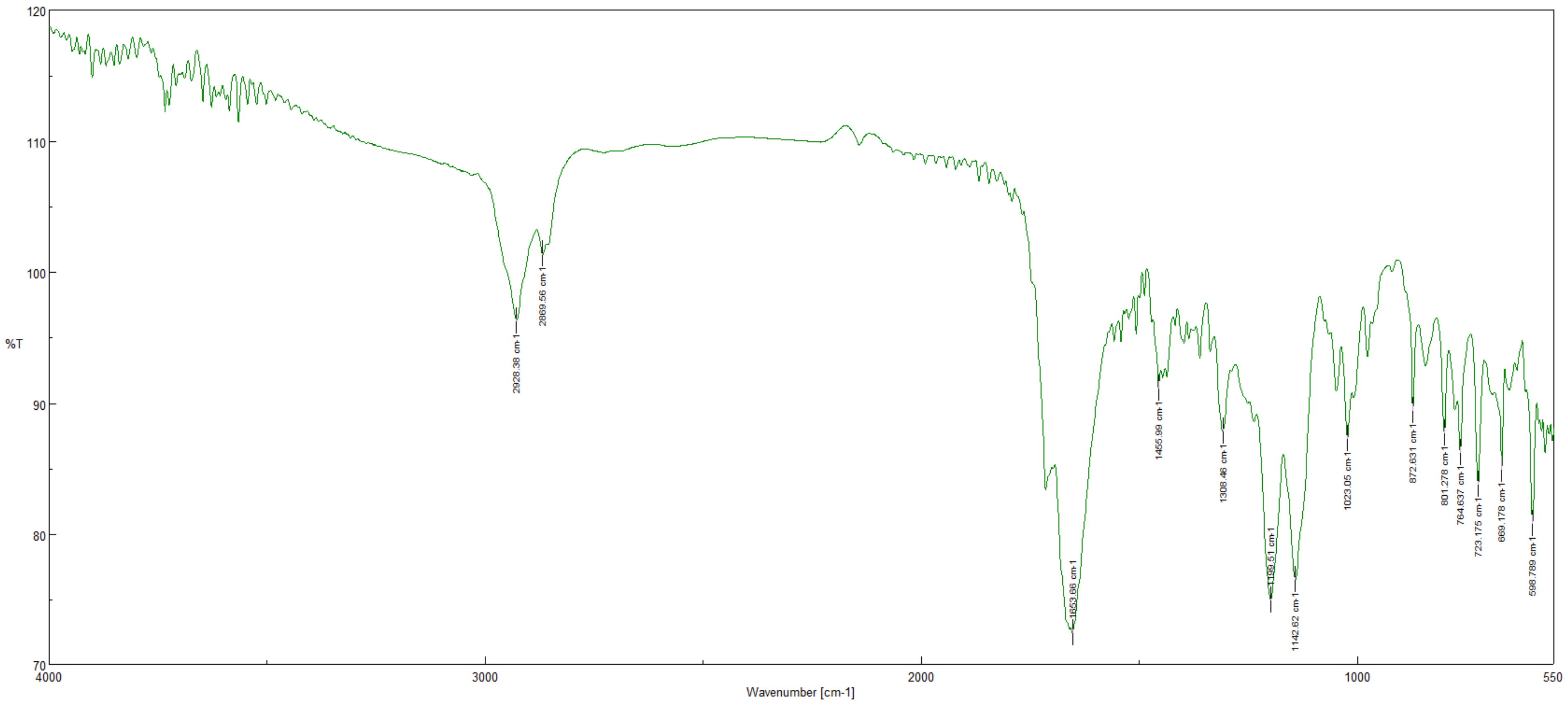

Figure S7. FT-IR spectrum of ircinianin (**1**).

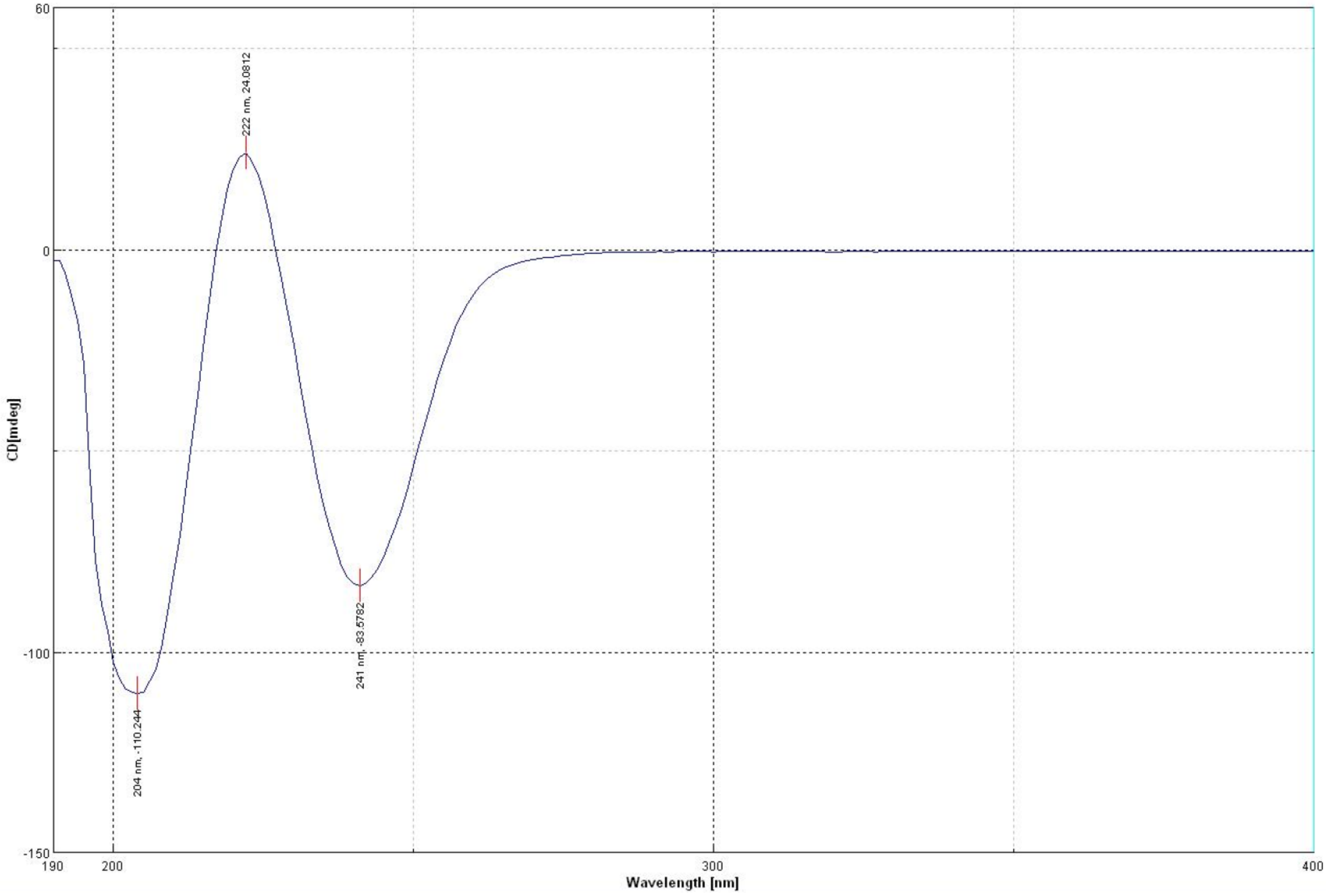

Figure S8. CD-spectrum of ircinianin (**1**) in MeOH.

Ircinianin (1) was isolated as a white powder from the 90% and 100% fraction and the molecular formula was deduced as $C_{25}H_{33}O_4$ requiring ten degrees of unsaturation – based on (+)-HRESIMS analysis ($[M+H]^+$ at *m/z* 379.2373 and 379.2376, respectively, calcd. as 379.2373, see Figures S4 and S6). ^{1}H, ^{13}C and DEPT 135 experiments (see Table 1, Figures S11-13) insinuated a furano sesterterpenoid with a tricyclic core. The ^{1}H showed three furan singlets at δ_H 7.38 (1H), 7.26 (1H) and 6.30 (1H) and additionally four signals of methyl groups (δ_H 0.92 (3H), 1.57 (3H), 1.64 (3H), 1.71 (3H)) [5]. The assumption of a mono substituted furan moiety was encouraged by four signals in ^{13}C / DEPT135 spectra (δ_C 144.0 (CH), 140.3 (CH), 126.5 (C), 112.1 (CH)), the characteristic carbon shifts of a tetronic acid pattern (δ_C 179.2 (C), 177.7 (C) and 97.5 (C)) could be also deduced [3]. Furthermore, four olefinic carbons with resonances at δ_C 123.6 (CH), 125.0 (CH), 136.6 (C), 137.1 (C) were detected, whereby the two quaternary carbons exhibited a characteristic shift caused by a methyl-substitution. Together with four methyl (δ_C 16.3, 20.7, 20.8, 6.1), five methylene (δ_C 25.3, 27.3, 29.5, 33.6, 40.5), four methine (δ_C 33.2, 46.2, 48.7, 52.0) and one quaternary carbon (δ_C 86.9), the ircinianin scaffold was completed. The subtraction of eight double bound equivalents (6x double bounds, 1x furan ring and 1x tetronic acid ring) from the total of ten degrees of unsaturation, pointed out two additional ring closures, which was also in accordance with a potential indene motive within the tricyclic indene-spirotetronic acid formation. From the results of ^{1}H-^{1}H-TOCSY (see Figure S16) together with ^{1}H-^{1}H-COSY and ^{1}H-^{13}C HMBC experiments, three major fragments could be assigned. One fragment (C-15 to C-20) represented the five-membered ring of the indene motif with a methyl group attached to C-18, while the second fragment (C-8 to C-14) was a combination of parts of the indene together with the alkyl linker with double bonds incorporated between C-12 to C-13 and C-8 to C-10, whereas C-8 was substituted with a further methyl group. The third fragment (C-1 to C-7) covered the furan ring together with the remaining three carbons of the alkyl linker. Diagnostic ^{1}H-^{13}C HMBC correlations could be used to combine these fragments to the known scaffold, whereby through space correlations of a ^{1}H-^{1}H-NOESY experiment were used to corroborate the molecular architecture in the polycyclic core unit. The absolute configuration was secured by X-ray analysis [26] and CD-spectroscopy (Figure S8) [23].

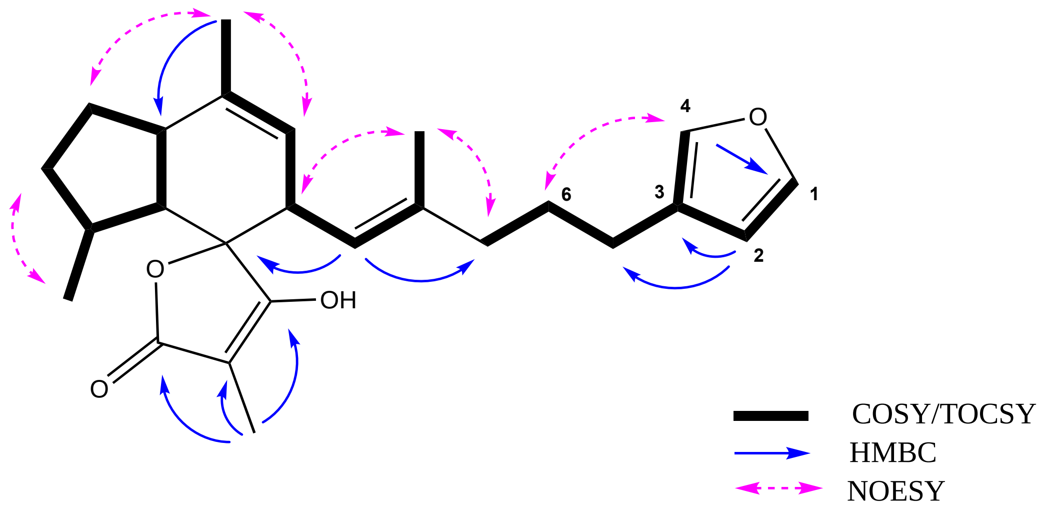

Figure S9. Key 2D-NMR correlations for **1**.

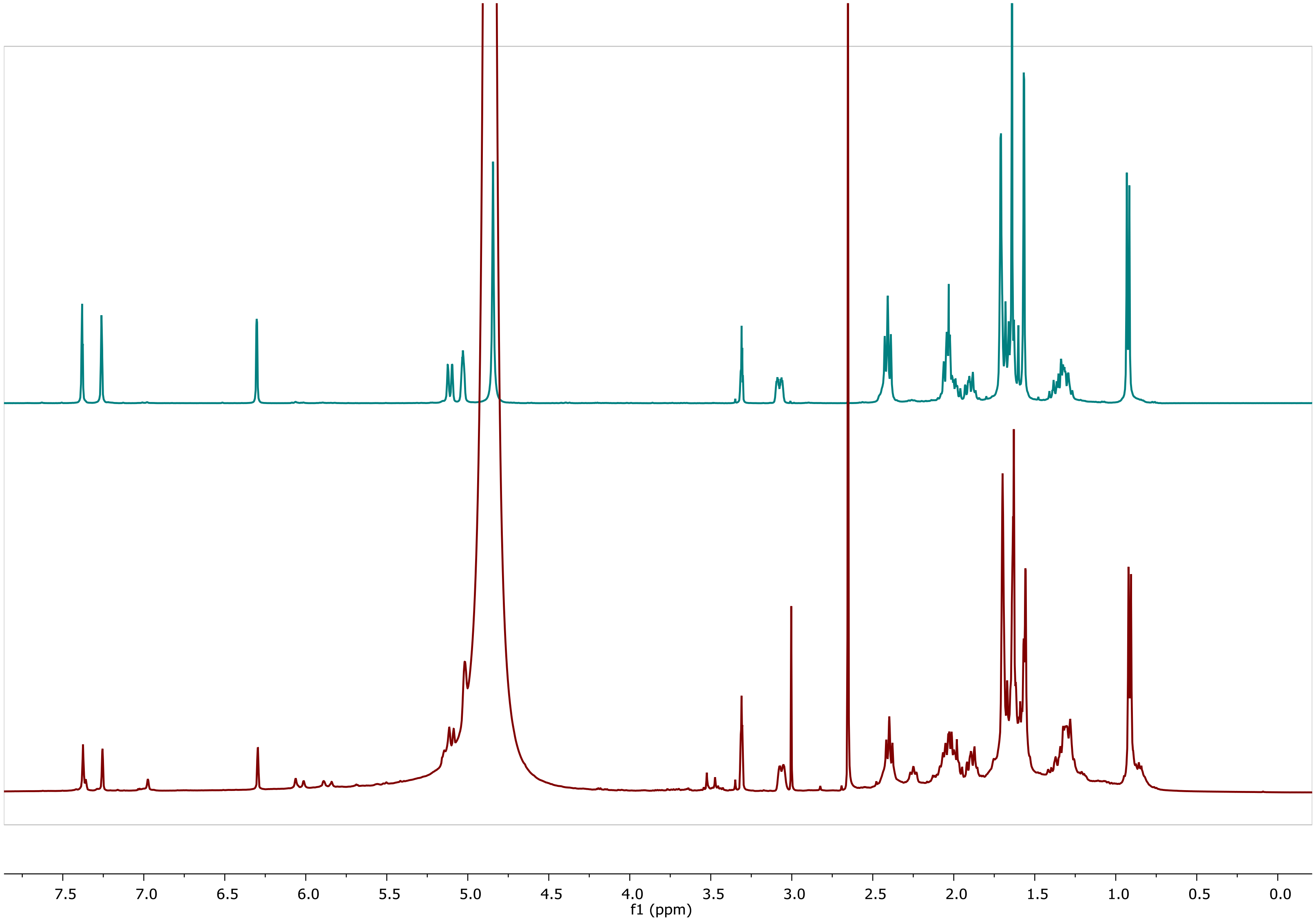

Figure S10. 400 MHz ^{1}H NMR spectrum of ircinianin (**1**, 90%-fraction, top) and ircinianin (**1**,100%-fraction, bottom) in d_4-MeOH.

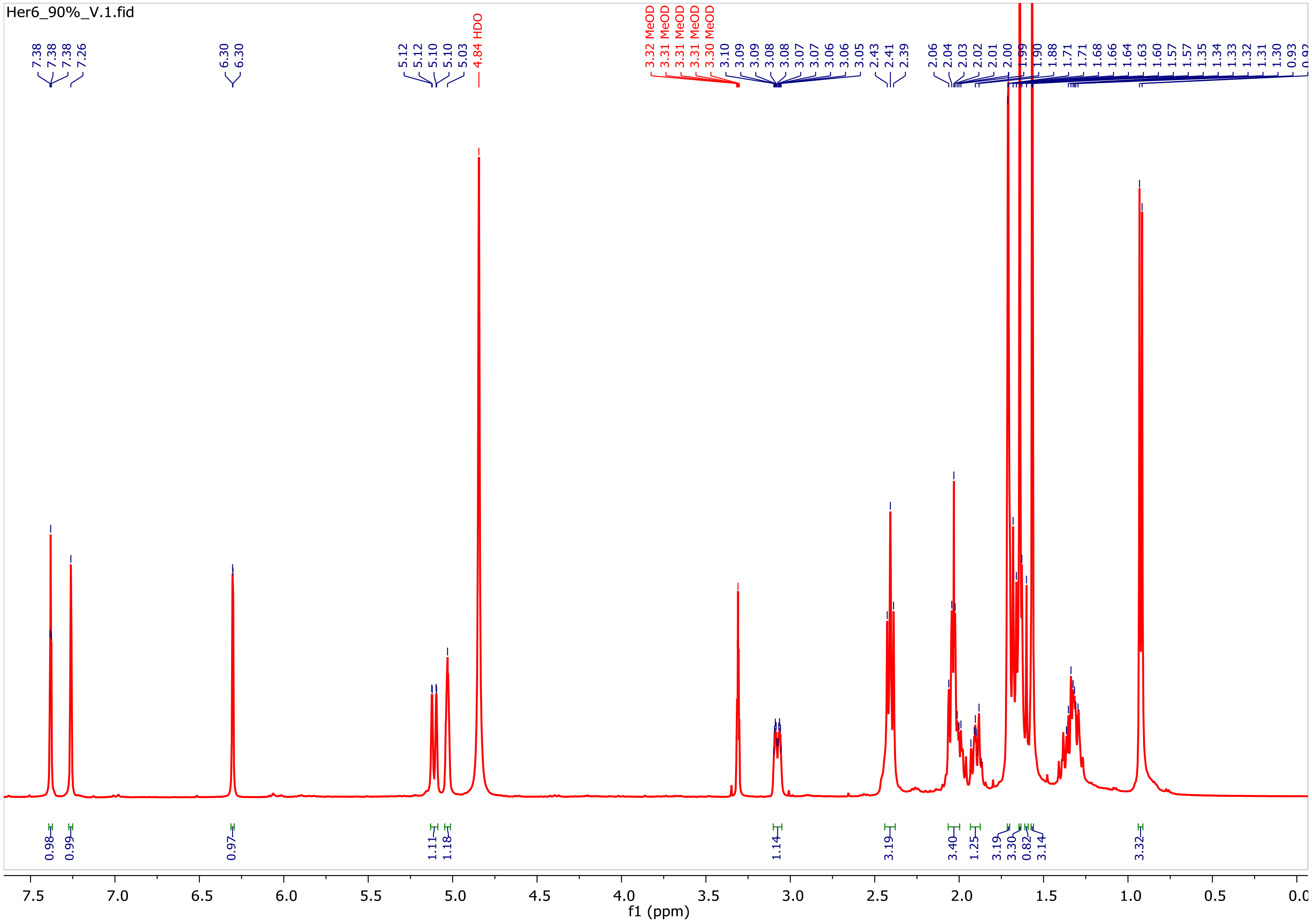

Figure S11. 400 MHz ^{1}H NMR spectrum of ircinianin (**1**, 90%-fraction) in d_4-MeOH.

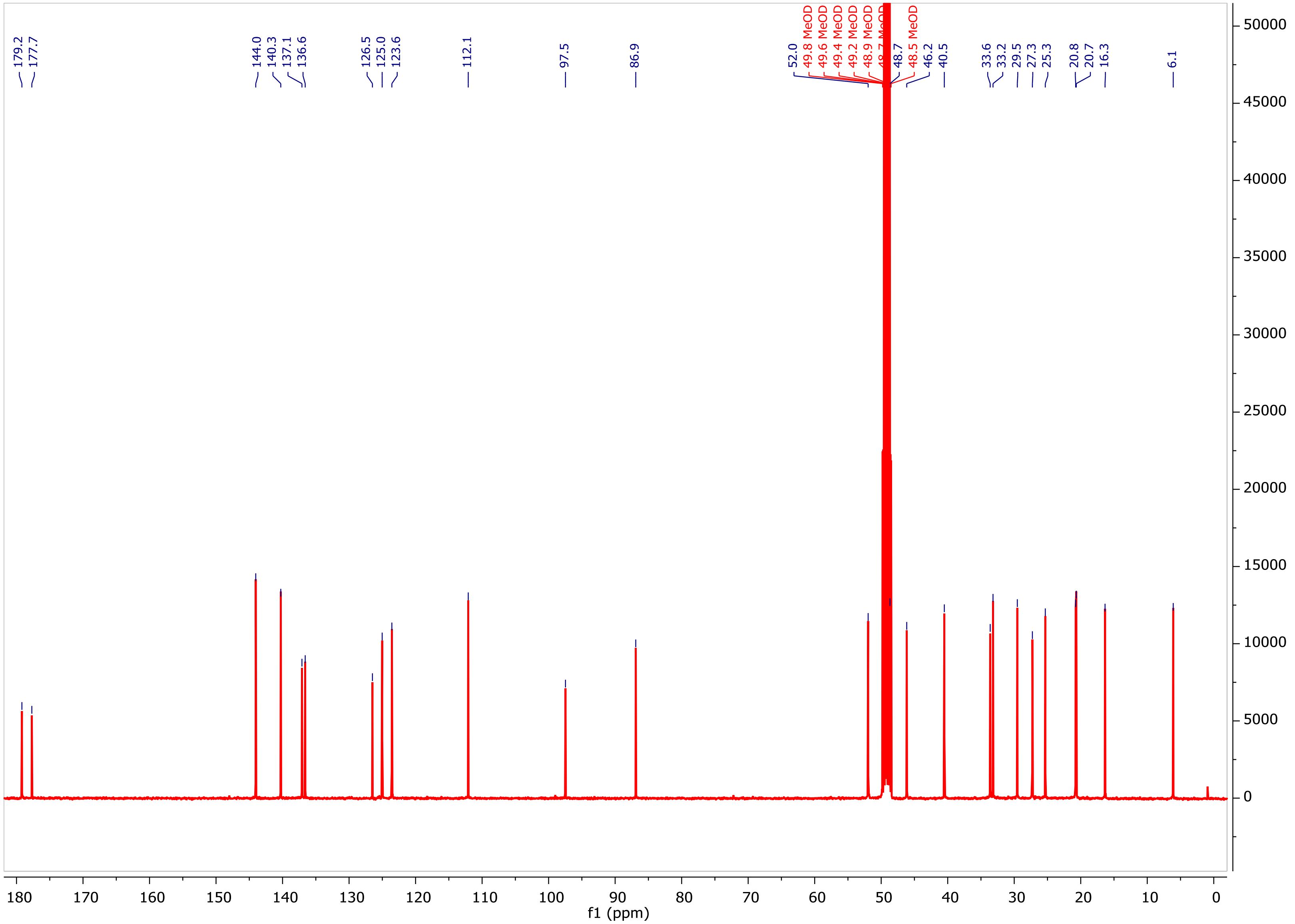

Figure S12. 100 MHz ^{13}C NMR spectrum of ircinianin (**1**, 90%-fraction) in d_4-MeOH.

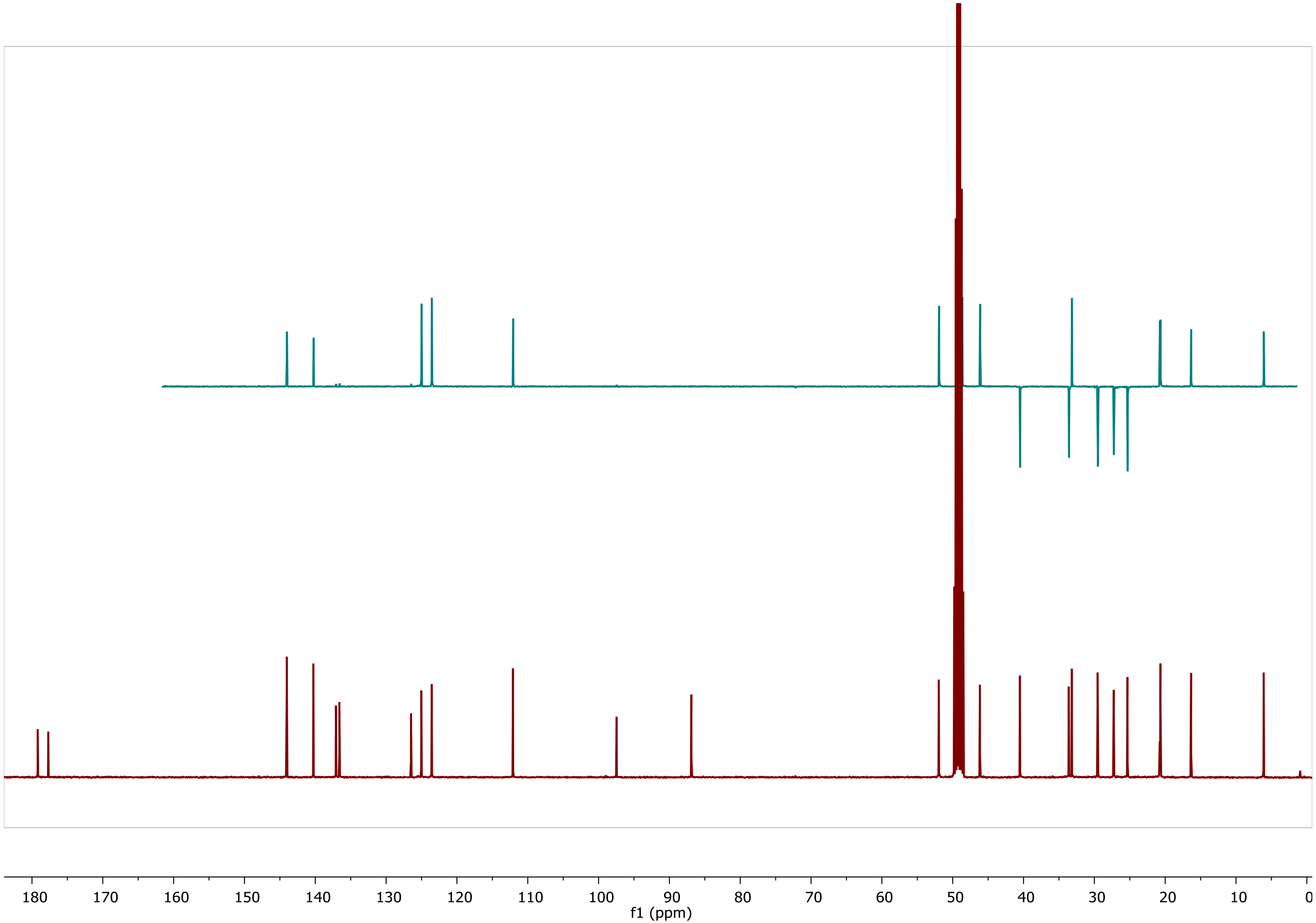

Figure 13. 100 MHz ^{13}C NMR spectrum (bottom) and 400 MHz DEPT135 NMR spectrum (top) of ircinianin (**1**, 90%-fraction) in d_4-MeOH.

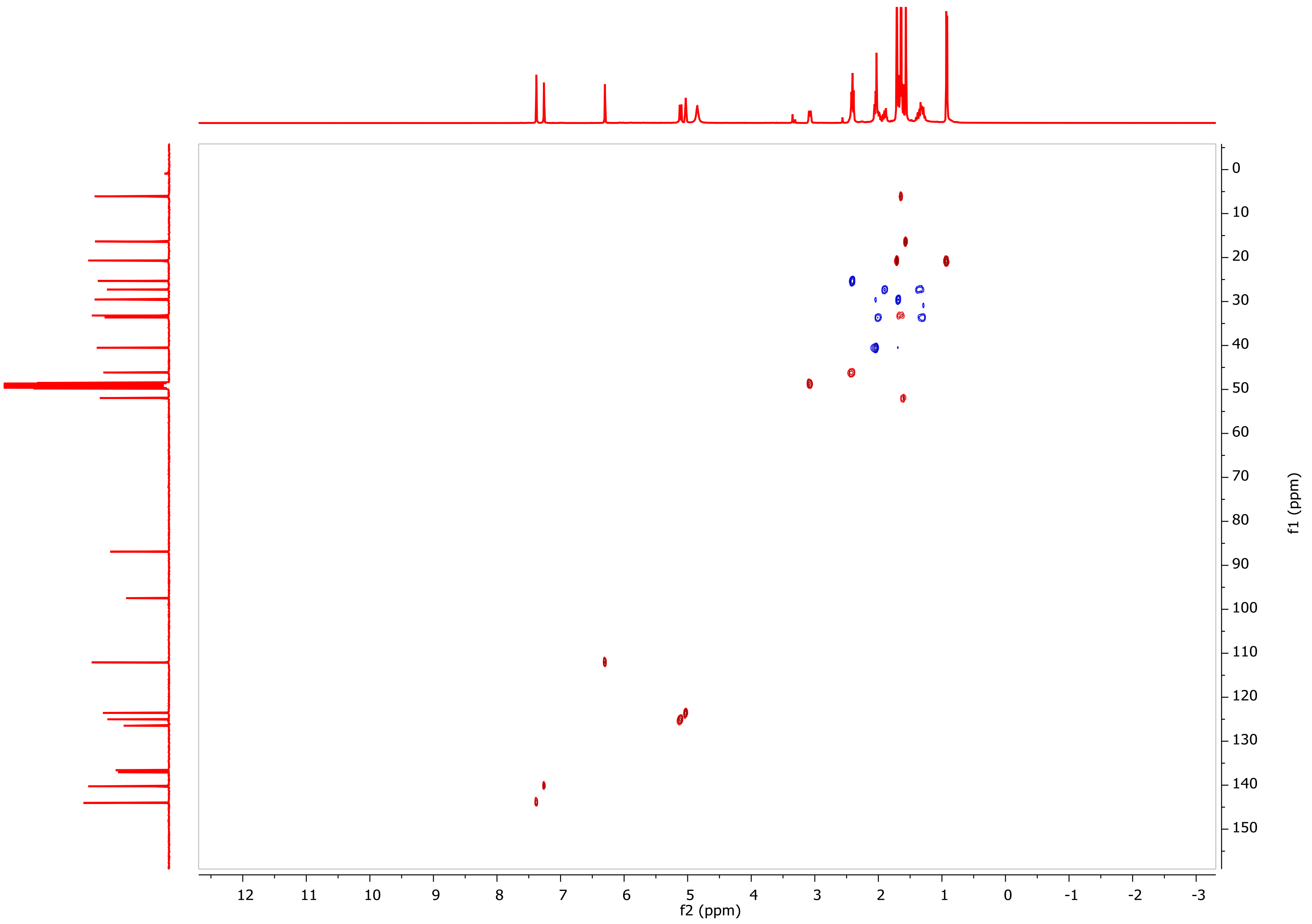

Figure S14. 400 MHz multiplicity edited ^{1}H-^{13}C HSQC NMR spectrum of ircinianin (**1**, 90%-fraction) in d_4-MeOH.

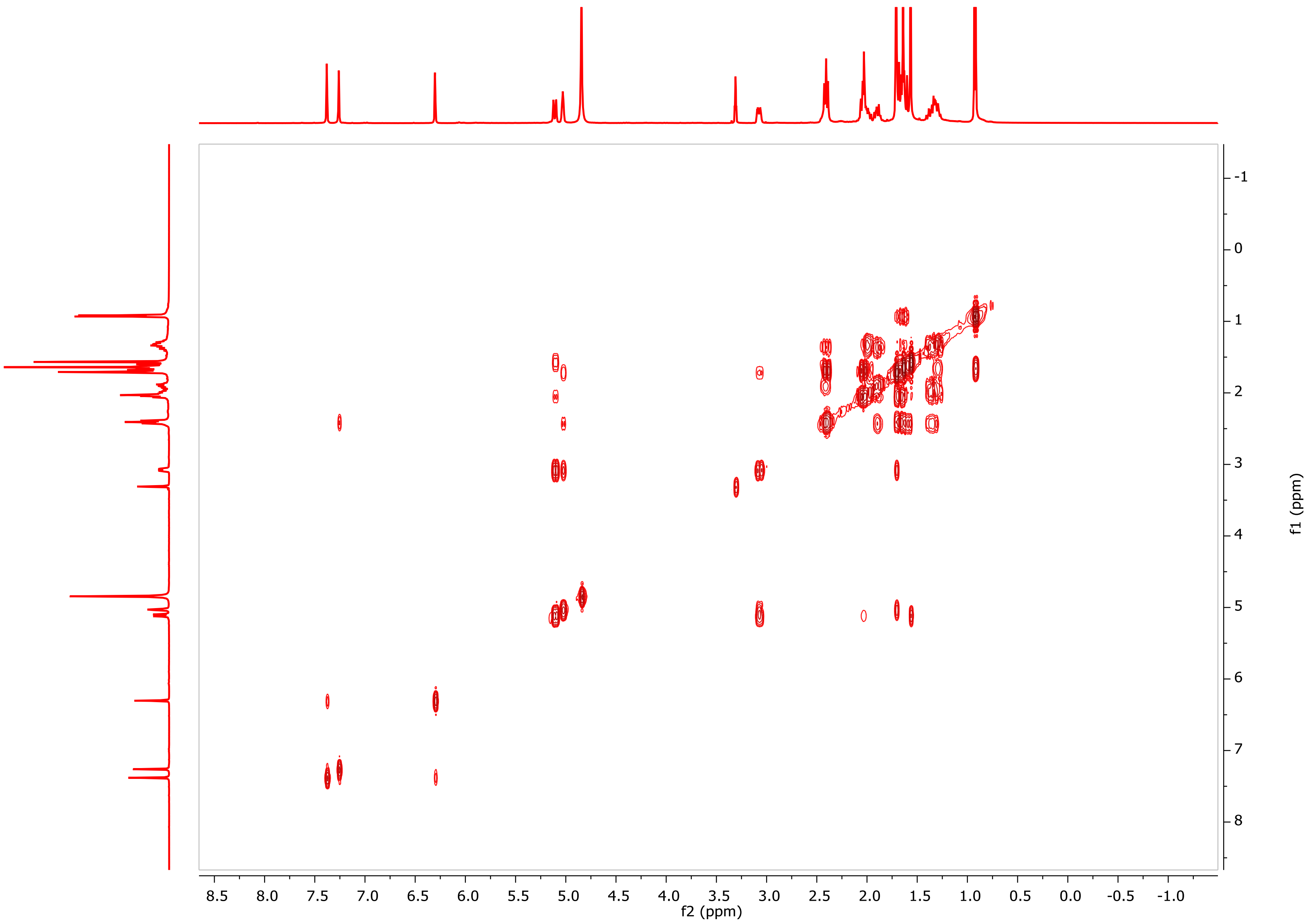

Figure S15. 400 MHz ^{1}H-^{1}H COSY NMR spectrum of ircinianin (**1**, 90%-fraction) in d_4-MeOH.

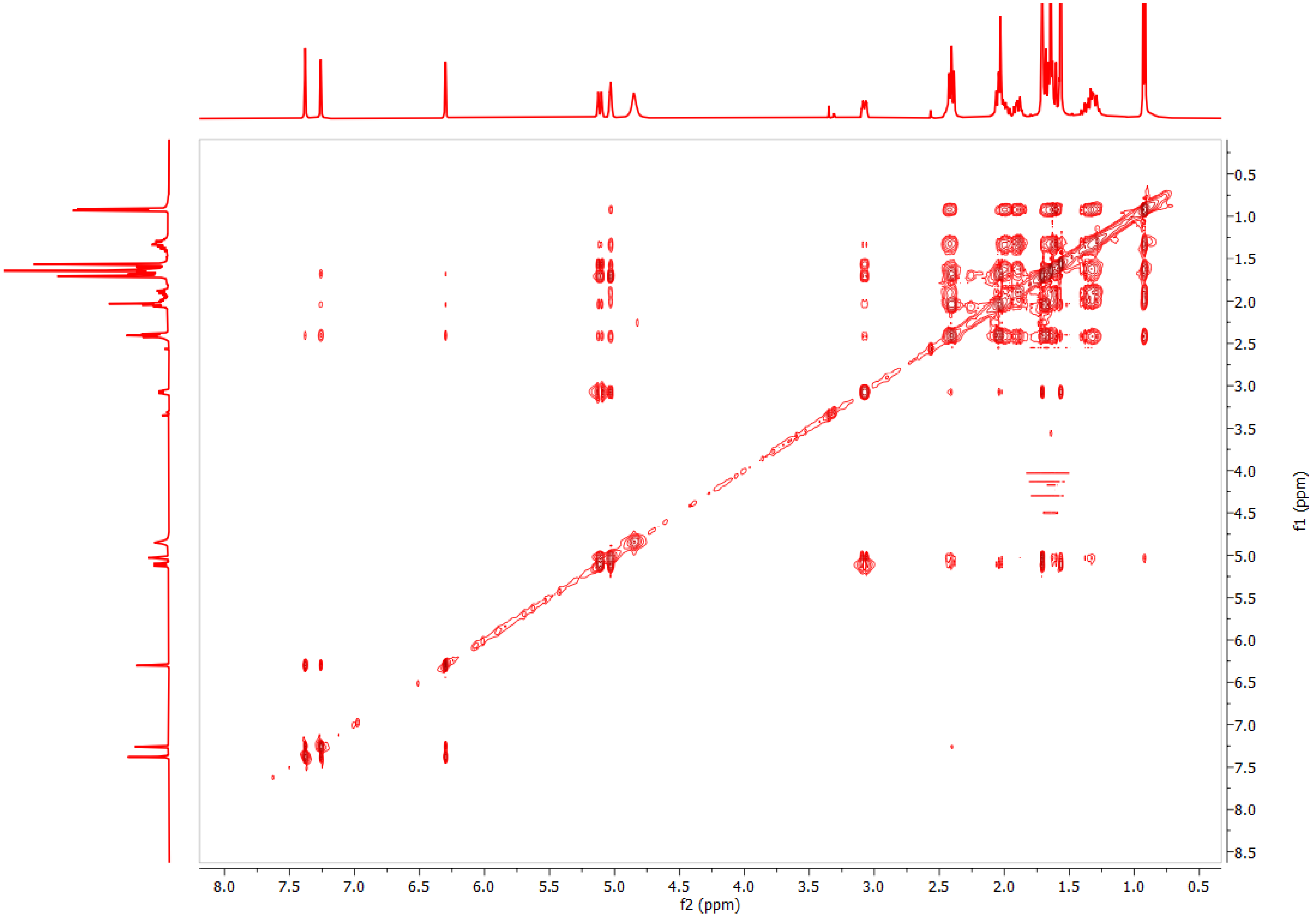

Figure S16. 400 MHz ^{1}H-^{1}H TOCSY NMR spectrum of ircinianin (**1**, 90%-fraction) in d_4-MeOH.

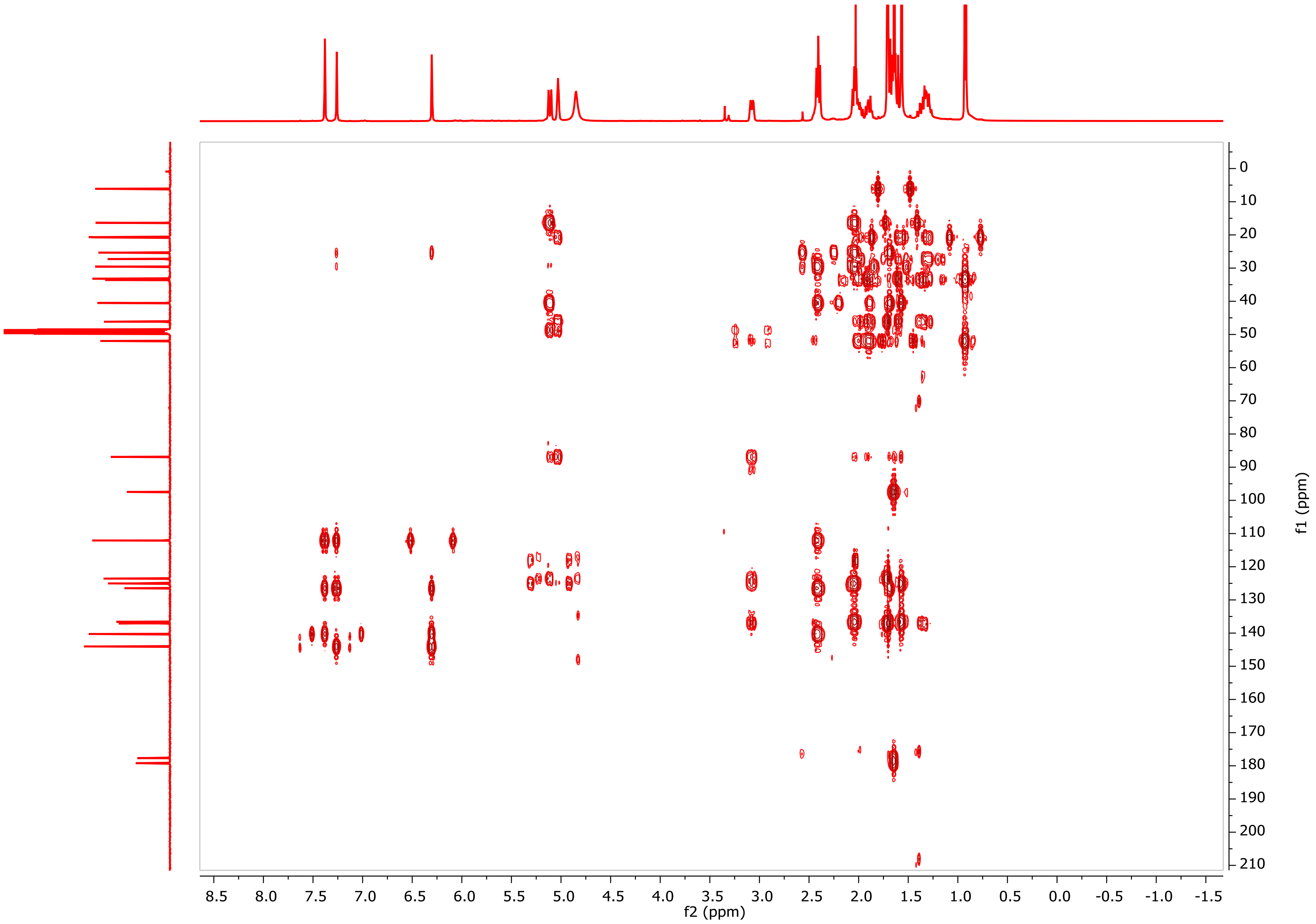

Figure S17. 400 MHz ^{1}H-^{13}C HMBC NMR spectrum of ircinianin (**1**, 90%-fraction) in d_4-MeOH.

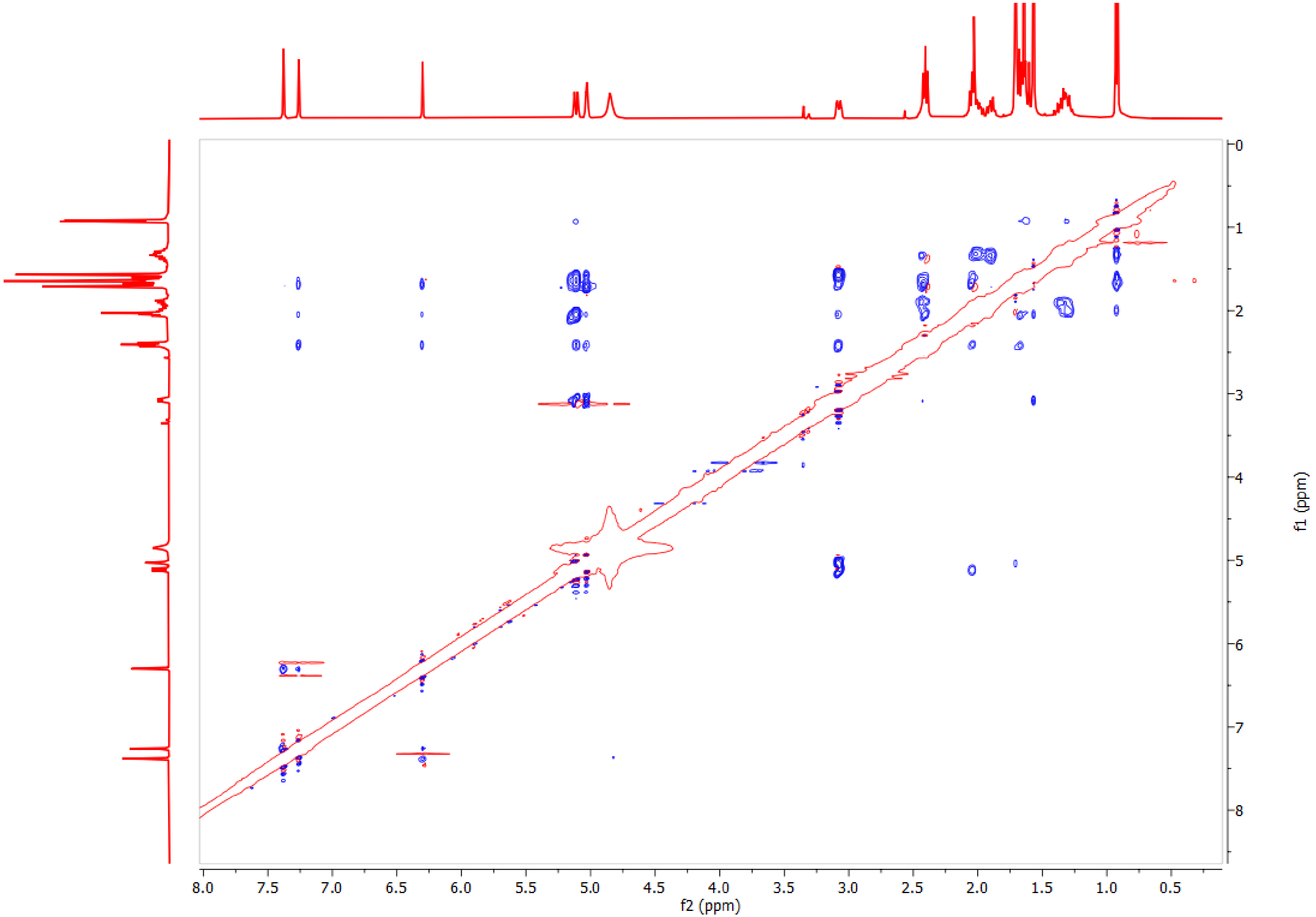

Figure S18. 400 MHz ^{1}H-^{1}H NOESY NMR spectrum of ircinianin (**1**, 90%-fraction) in d_4-MeOH.

A

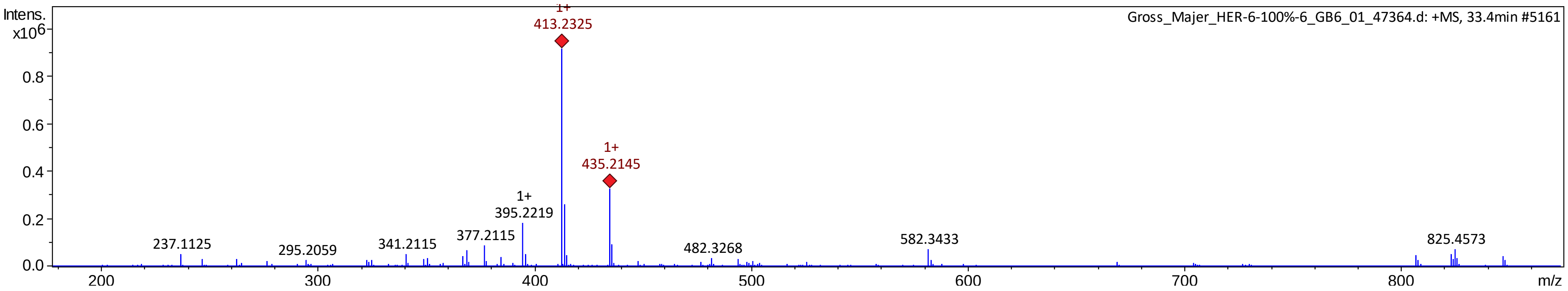

B

Meas. m/z	#	Ion Formula	m/z	err [ppm]	Mean err [ppm]	rdb	N-Rule	e⁻ Conf	mSigma	Std I
413.2325	1	$C_{25}H_{33}O_5$	413.2323	-0.7	-0.9	10.0	ok	even	5.5	8.9
413.2325	2	$C_{26}H_{29}N_4O$	413.2336	2.6	2.0	15.0	ok	even	9.3	11.9
413.2325	3	$C_{21}H_{29}N_6O_3$	413.2296	-7.2	-8.1	11.0	ok	even	19.7	32.5

C

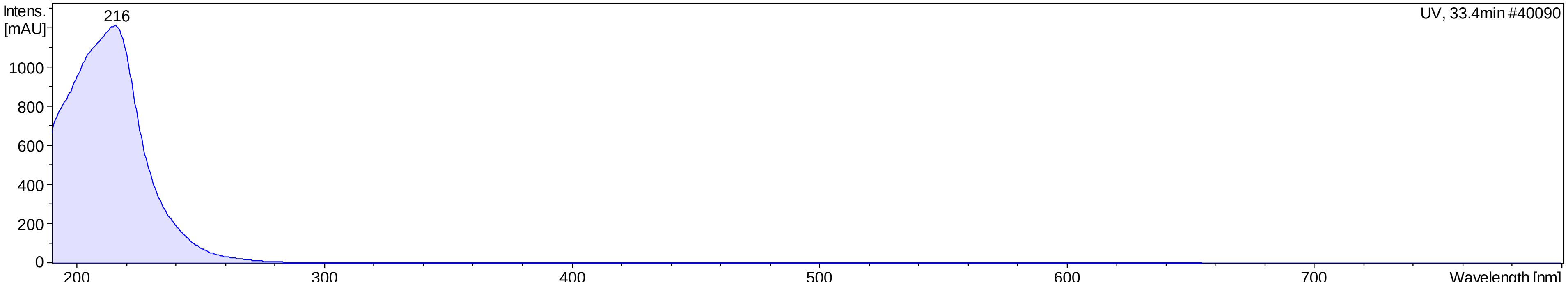

Figure S19. Oxoircinianin (**3**, 100%-fraction) MS-Analysis – A: HRMS-result; B: predicted molecular formula; C: extracted UV-Profile.

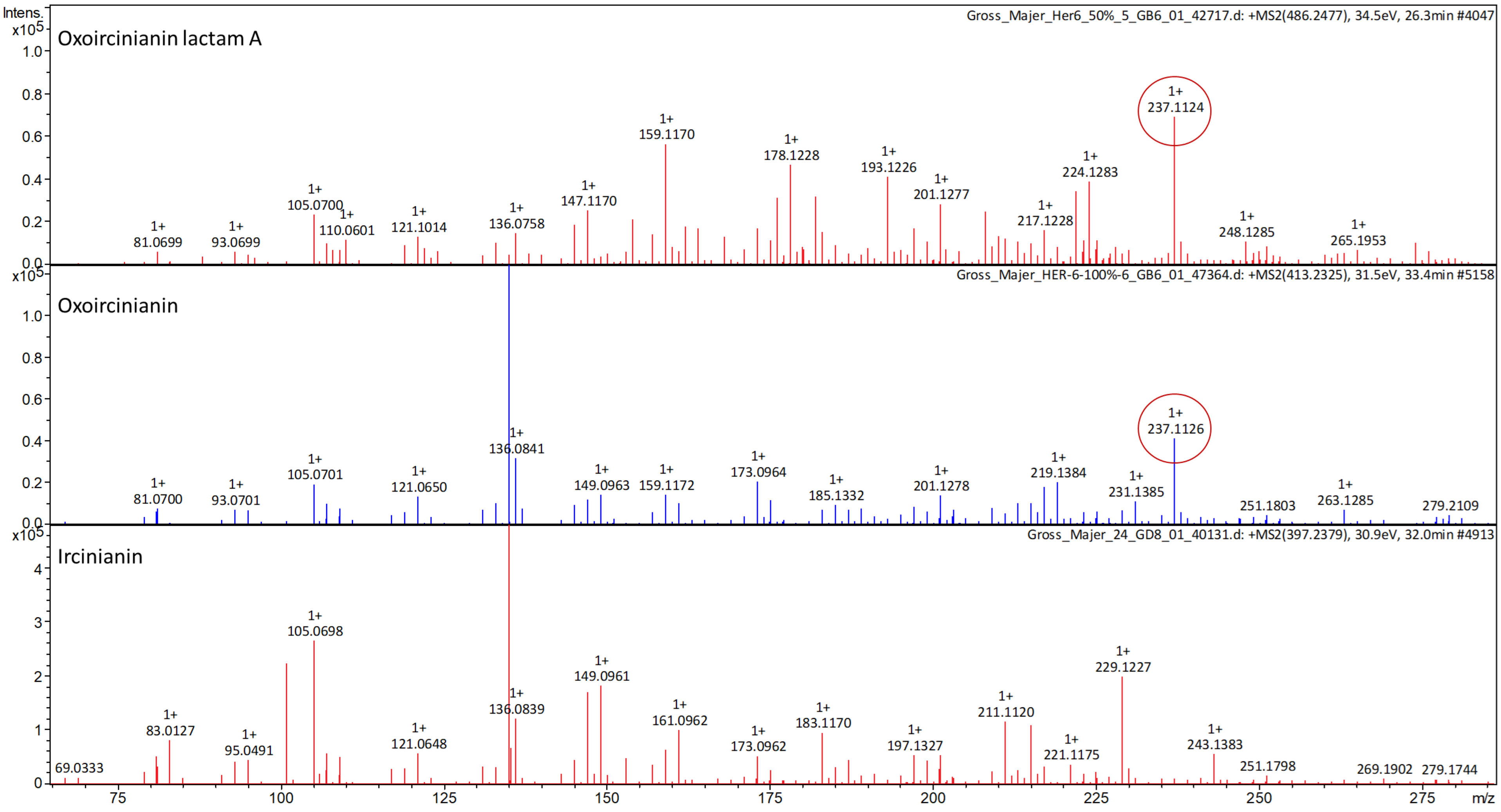

Figure S20. Comparison of the MS^2 spectra of ircinianin (**1**), oxoircinianin lactam A (**5**) and oxoircinianin (**3**) – the characteristic fragment of **5** and **3**, indicating the "oxo"-skeleton, is highlighted. Oxoircinianin (**3**) was characterized by HRESIMS. The scaffold affiliation was proven by MS^2 analysis in comparison with **1** and **5** which exhibited comparable fragmentation patterns. Furthermore, a ^{13}C NMR experiment of an oxoircinianin enriched fraction confirmed the "oxo"-molecular architecture in the tetronic acid motif.

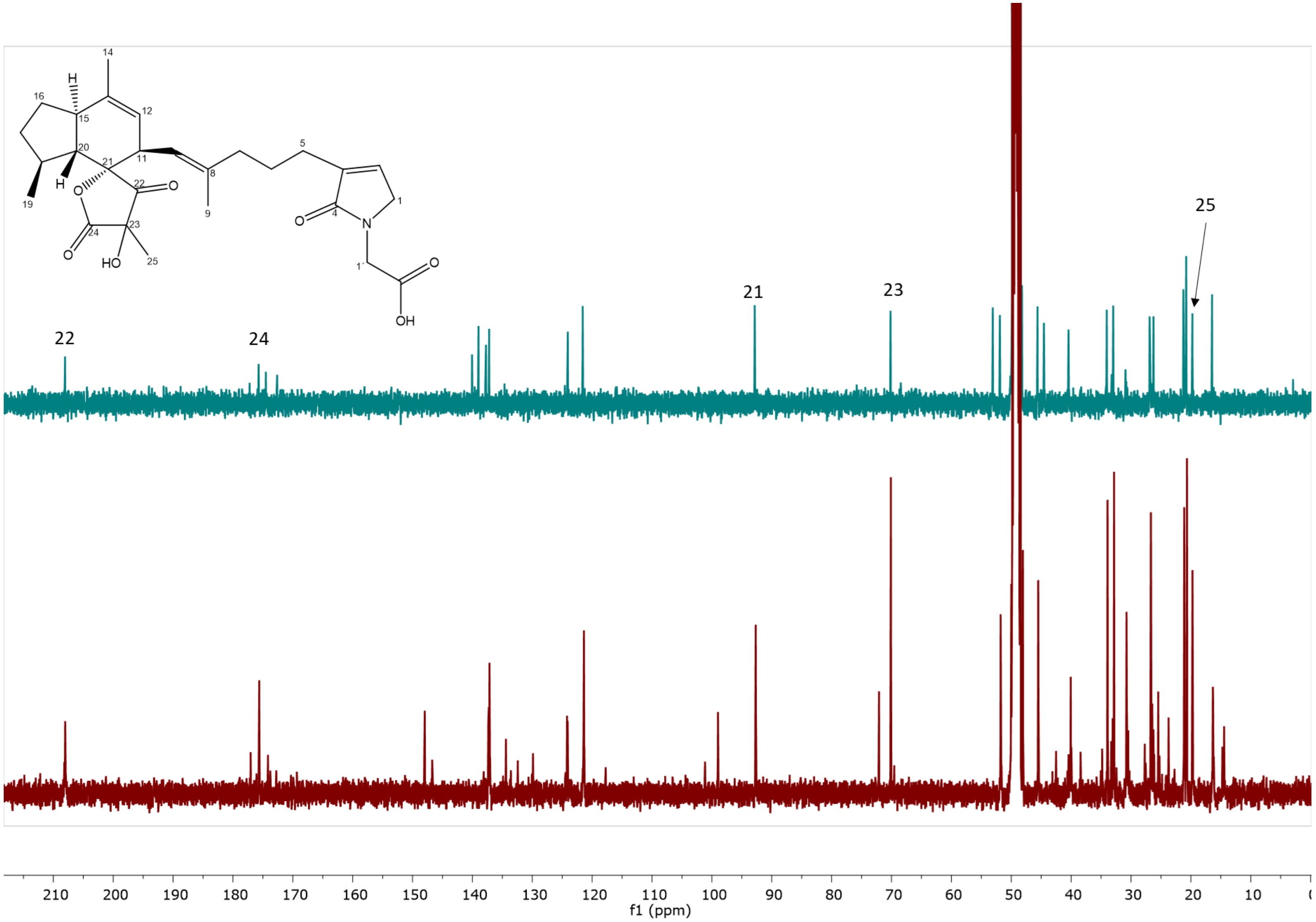

Figure S21. ^{13}C (100 MHz) of oxoircinianin lactam (**5**) A (top) and ^{13}C (100 MHz) of oxoircinianin (**3**) containing fraction (bottom) in d_4-MeOH – Peaks representing the “oxo”-form of the tetronic acid motif are labeled.

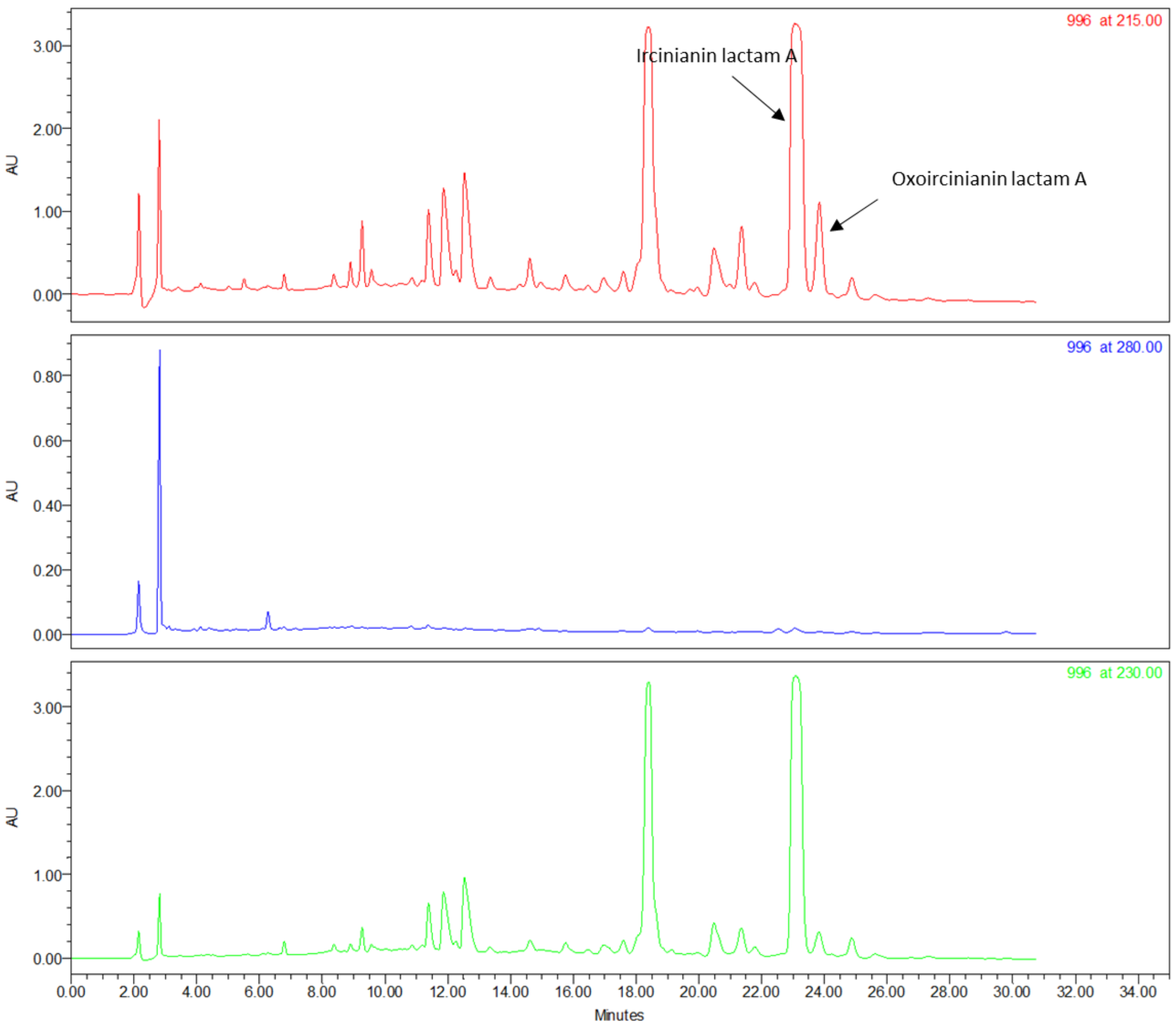

Figure S22. Chromatogram of the 50%-LC-fraction.

A

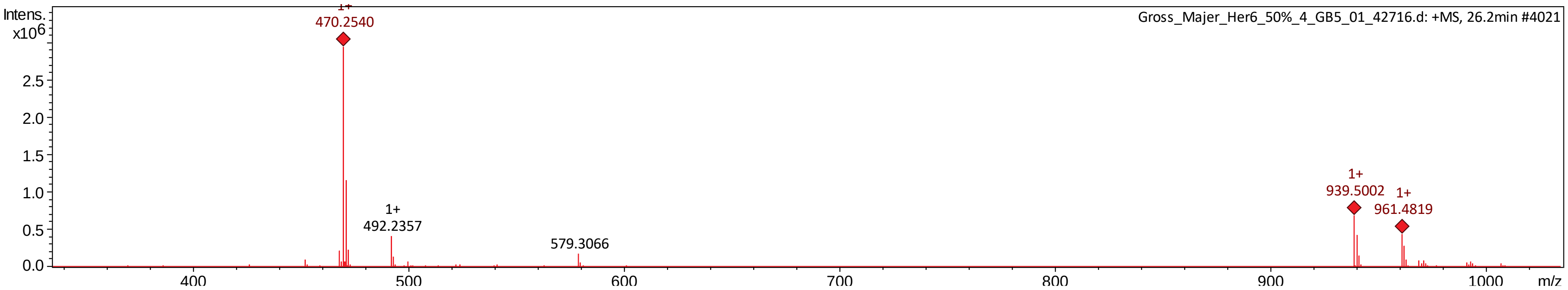

B

Meas. m/z	#	Ion Formula	m/z	err [ppm]	Mean err [ppm]	rdb	N-Rule	e⁻ Conf	mSigma	Std I
470.2540	1	$C_{28}H_{32}N_5O_2$	470.2551	2.3	2.3	16.0	ok	even	35.6	56.5
470.2540	2	$C_{27}H_{36}NO_6$	470.2537	-0.5	-0.3	11.0	ok	even	46.7	72.9
470.2540	3	$C_{24}H_{28}N_{11}$	470.2524	-3.4	-4.2	17.0	ok	even	55.4	77.6
470.2540	4	$C_{23}H_{32}N_7O_4$	470.2510	-6.2	-6.7	12.0	ok	even	68.0	95.0
470.2540	5	$C_{16}H_{36}N_7O_9$	470.2569	6.3	5.4	3.0	ok	even	110.2	161.1
470.2540	6	$C_{13}H_{28}N_{17}O_3$	470.2556	3.4	1.3	9.0	ok	even	111.3	166.9
470.2540	7	$C_{12}H_{32}N_{13}O_7$	470.2542	0.6	-1.2	4.0	ok	even	123.9	186.6
470.2540	8	$C_9H_{24}N_{23}O$	470.2529	-2.3	-5.8	10.0	ok	even	125.1	192.9

C

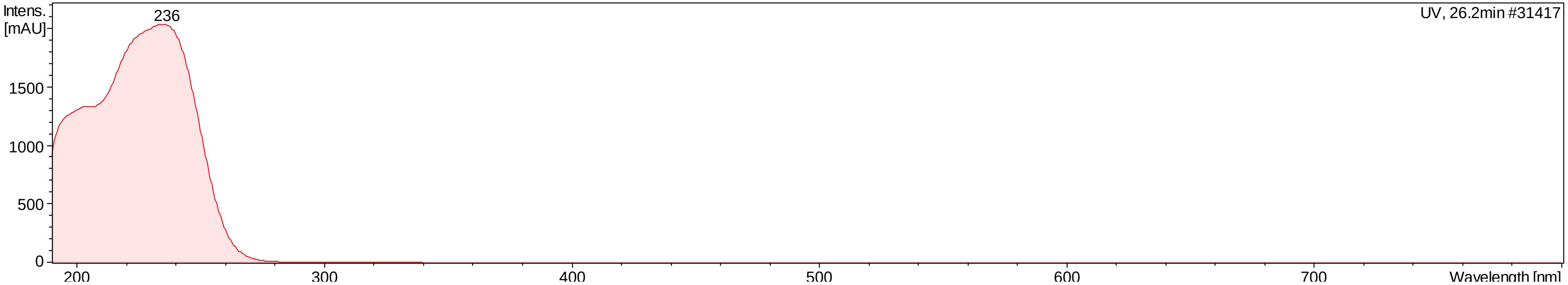

Figure S23. Ircinianin lactam A (**4**, 50%-fraction) MS-Analysis – A: HRMS-result; B: predicted molecular formula; C: extracted UV-Profil.

Table S1. ^{1}H (400 MHz) and ^{13}C (100 MHz) NMR data of ircinianin lactam A (**4**) in d_4-MeOH. Chemical shifts are given in ppm.

(-) Ircinianin lactam A (4)		
**Position**	**δ_H (mult., *J* [Hz])**	**δ_C**
**1**	4.07, m^{A}	53.2
**2**	6.94, m	139.1
**3**	-	140.1
**4**	-	172.5
**5**	2.22-2.29, m	26.2
**6**	1.71, m^{B}	27.0
**7**	2.08, dd (7.2, 6.9)	40.4
**8**	-	136.2
**9**	1.57, d (1.1)	16.2
**10**	5.13, d (10.2)	125.5
**11**	3.07, dm (10.2)	48.8^{E}
**12**	5.02, m	123.5
**13**	-	137.0
**14**	1.71, m^{B}	20.71^{F}
**15**	2.39-2.47 (m)	46.1
**16**	*a.* 1.87-1.92, m^{C}<br>*b.* 1.19-1.42, m^{D*}	27.3
**17**	*a.* 1.99, m^{C}<br>*b.* 1.19-1.42, m^{D*}	33.6
**18**	1.66-1.69, m*	33.2
**19**	0.93, m*	20.71^{F}
**20**	1.58-1.64, m	51.7
**21**	-	86.8
**22**	-	179.2
**23**	-	97.4
**24**	-	177.7
**25**	1.65, s	6.1
**1`**	4.07, br s	44.4
**2`**	-	174.6

$^{A/B/C/D/F}$ Overlapping signals within one column.
* Overlapped by n-butanol impurity.
^{E} Overlapped by solvent peak.

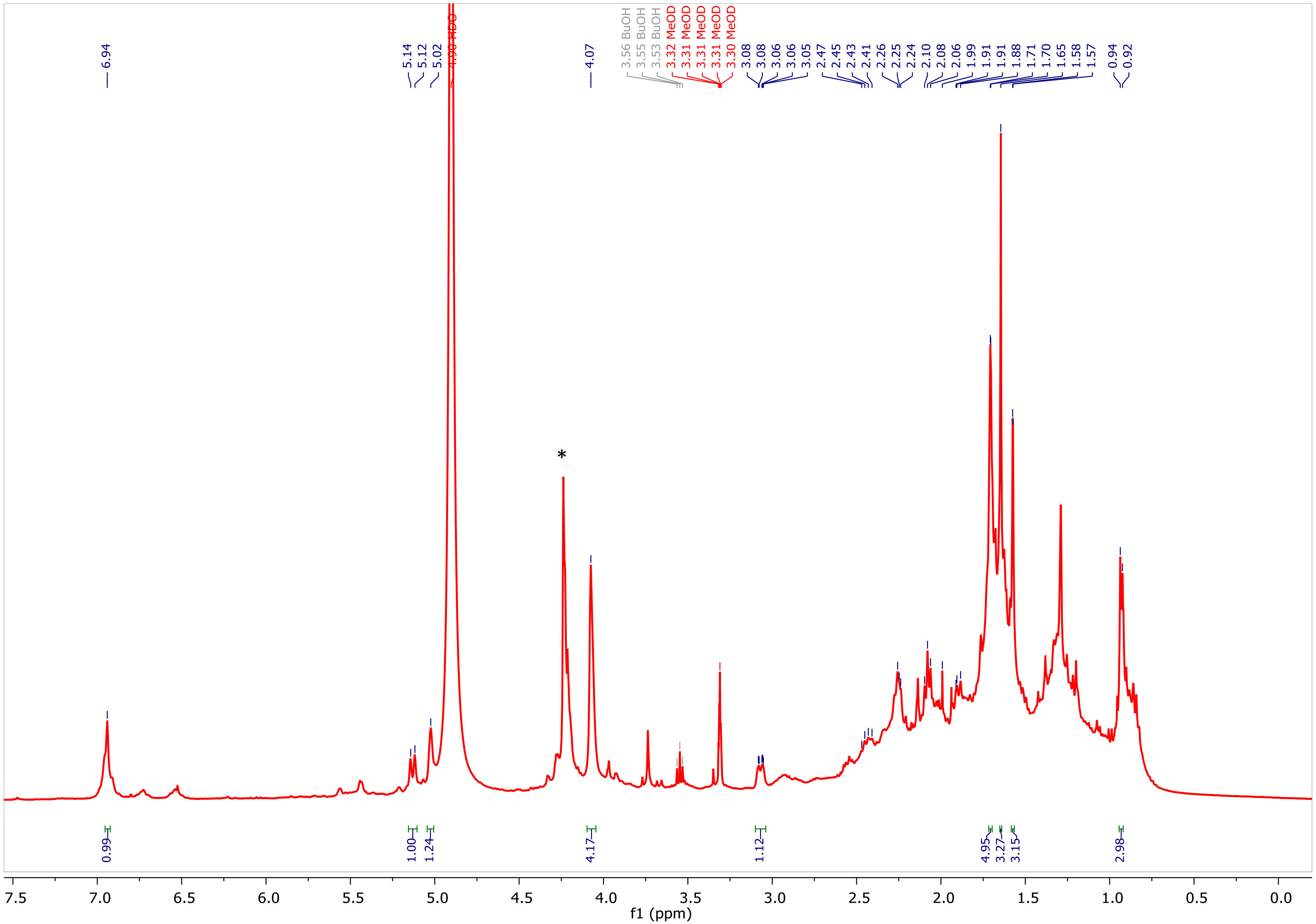

Figure S24. 400 MHz ^{1}H NMR spectrum of ircinianin lactam A (**4**, 50%-fraction) in d_4-MeOH. Asterisk denotes an impurity at 4.23 ppm.

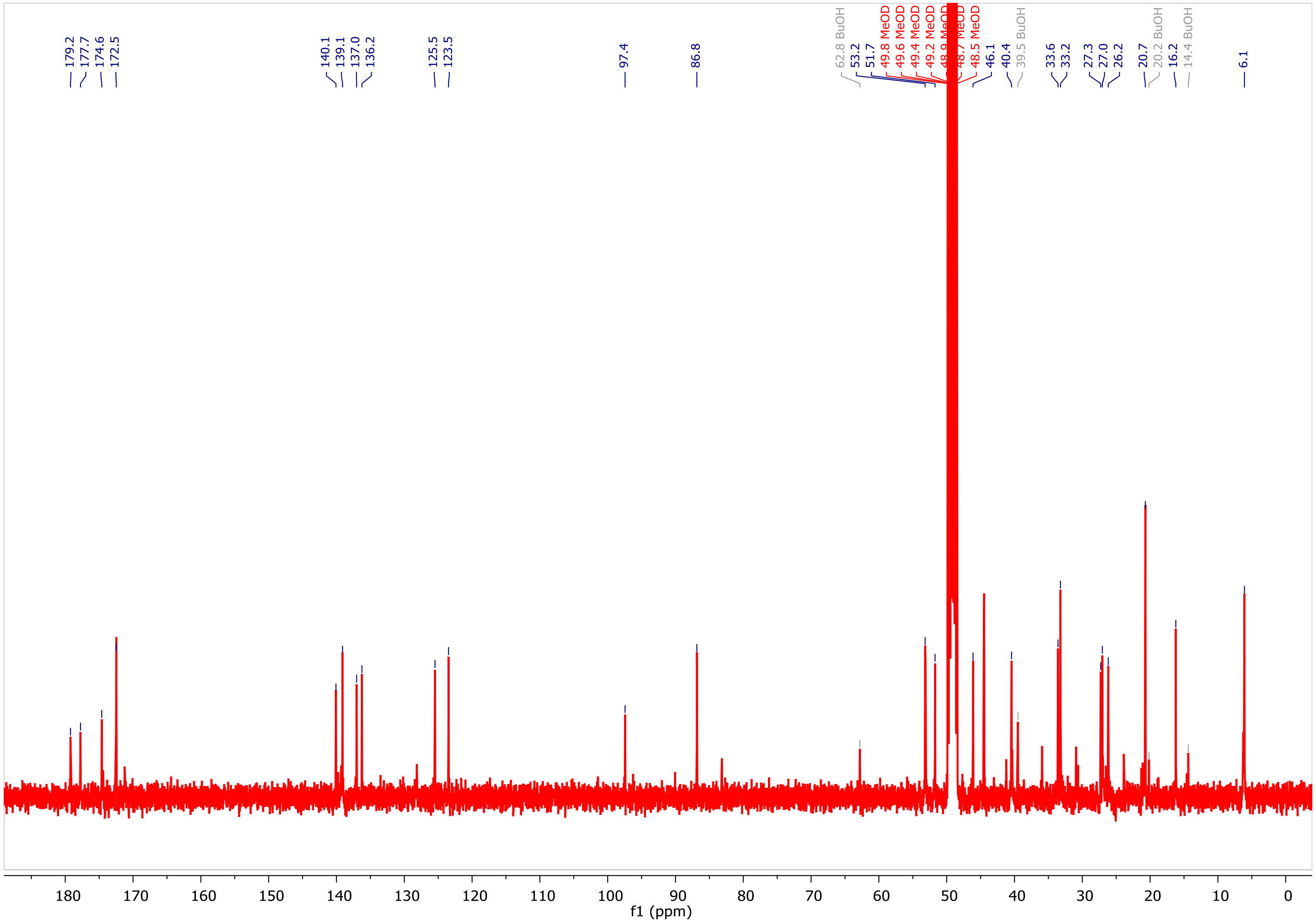

Figure S25. 100 MHz ^{13}C NMR spectrum of ircinianin lactam A (**4**, 50%-fraction) in d_4-MeOH.

A

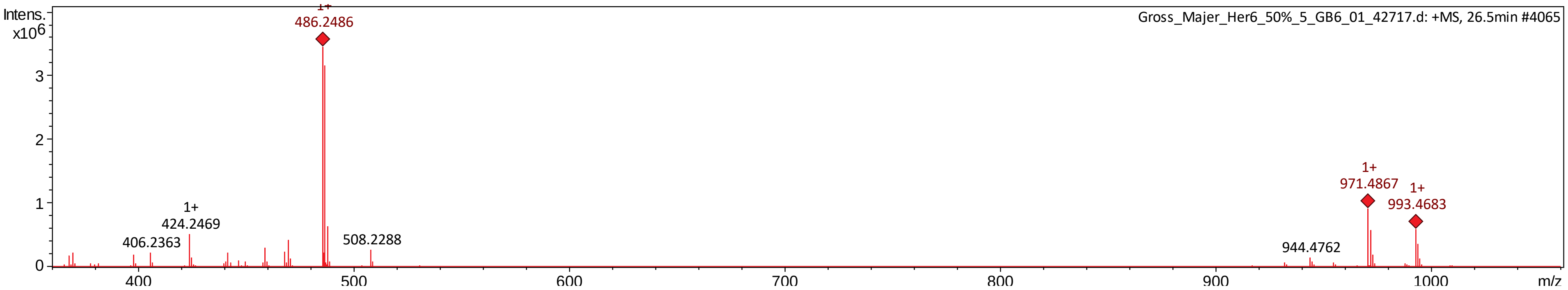

B

Meas. m/z	#	Ion Formula	m/z	err [ppm]	Mean err [ppm]	rdb	N-Rule	e⁻ Conf	mSigma	Std I
486.2486	1	$C_{28}H_{32}N_5O_3$	486.2500	2.9	4.1	16.0	ok	even	300.2	318.9
486.2486	2	$C_{27}H_{36}NO_7$	486.2486	0.1	1.6	11.0	ok	even	311.6	335.7
486.2486	3	$C_{23}H_{32}N_7O_5$	486.2459	-5.4	-4.7	12.0	ok	even	323.7	358.5
486.2486	4	$C_{22}H_{36}N_3O_9$	486.2446	-8.2	-7.2	7.0	ok	even	335.0	376.2
486.2486	5	$C_{24}H_{28}N_{11}O$	486.2473	-2.7	-2.2	17.0	ok	even	360.7	341.7
486.2486	6	$C_{14}H_{24}N_{21}$	486.2518	6.6	5.4	14.0	ok	even	404.1	411.7
486.2486	7	$C_{16}H_{36}N_7O_{10}$	486.2518	6.7	7.1	3.0	ok	even	416.0	425.7
486.2486	8	$C_{13}H_{28}N_{17}O_4$	486.2505	3.9	3.1	9.0	ok	even	416.9	431.1
486.2486	9	$C_{12}H_{32}N_{13}O_8$	486.2491	1.1	0.7	4.0	ok	even	429.7	451.0
486.2486	10	$C_9H_{24}N_{23}O_2$	486.2478	-1.6	-3.6	10.0	ok	even	430.7	457.0

C

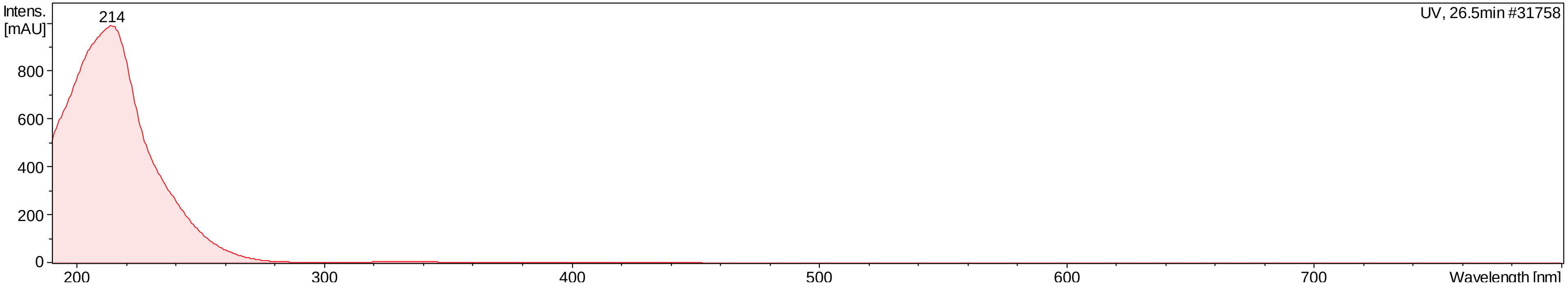

Figure S26. Oxoircinianin lactam A (**5**, 50%-fraction) MS-Analysis – A: HRMS-result; B: predicted molecular formula; C: extracted UV-Profile.

Table S2. 400 MHz ^{1}H and 100 MHz ^{13}C NMR-data of oxoircinianin lactam A (**5**) in d_4-MeOH. Chemical shifts are given in ppm.

(-)-Oxoircinianin lactam A (5)		
**Position**	**δ_H (mult., *J* [Hz])**	**δ_C**
**1**	4.07, m	53.1
**2**	6.96, m	139.0
**3**	-	140.1
**4**	-	174.5
**5**	2.26, br t (7.5)	26.3
**6**	1.64-1.70, m*	26.9^A
**7**	2.05, dd (7.3, 7,1)^B	40.4
**8**	-	137.7
**9**	1.58, d (1.1)	16.5
**10**	5.00, m^C	124.1
**11**	3.34, m^F	48.2
**12**	5.03, m^C	121.6
**13**	-	137.2
**14**	1.72, d (1.2)^E	21.3
**15**	2.36-2.49, m	45.6
**16**	*a.* 1.89-1.95, m<br>*b.* 1.28-1.36, m^D	26.8^A
**17**	*a.* 2.05, m^B<br>*b.* 1.28-1.36, m^D	34.1
**18**	1.73, m^E	33.0
**19**	0.83, d (6.1)	20.8
**20**	1.65-1.73, m*	51.9
**21**	-	92.9
**22**	-	208.0
**23**	-	70.2
**24**	-	175.7
**25**	1.38, s	19.8
**1´**	4.23, s	44.6
**2´**	-	172.6

^{A} Assignments interchangeable.
* Overlapped by other signals.
$^{B/C/D/E}$ Overlapping signals within one column.
^{F} Overlapped by solvent peak.

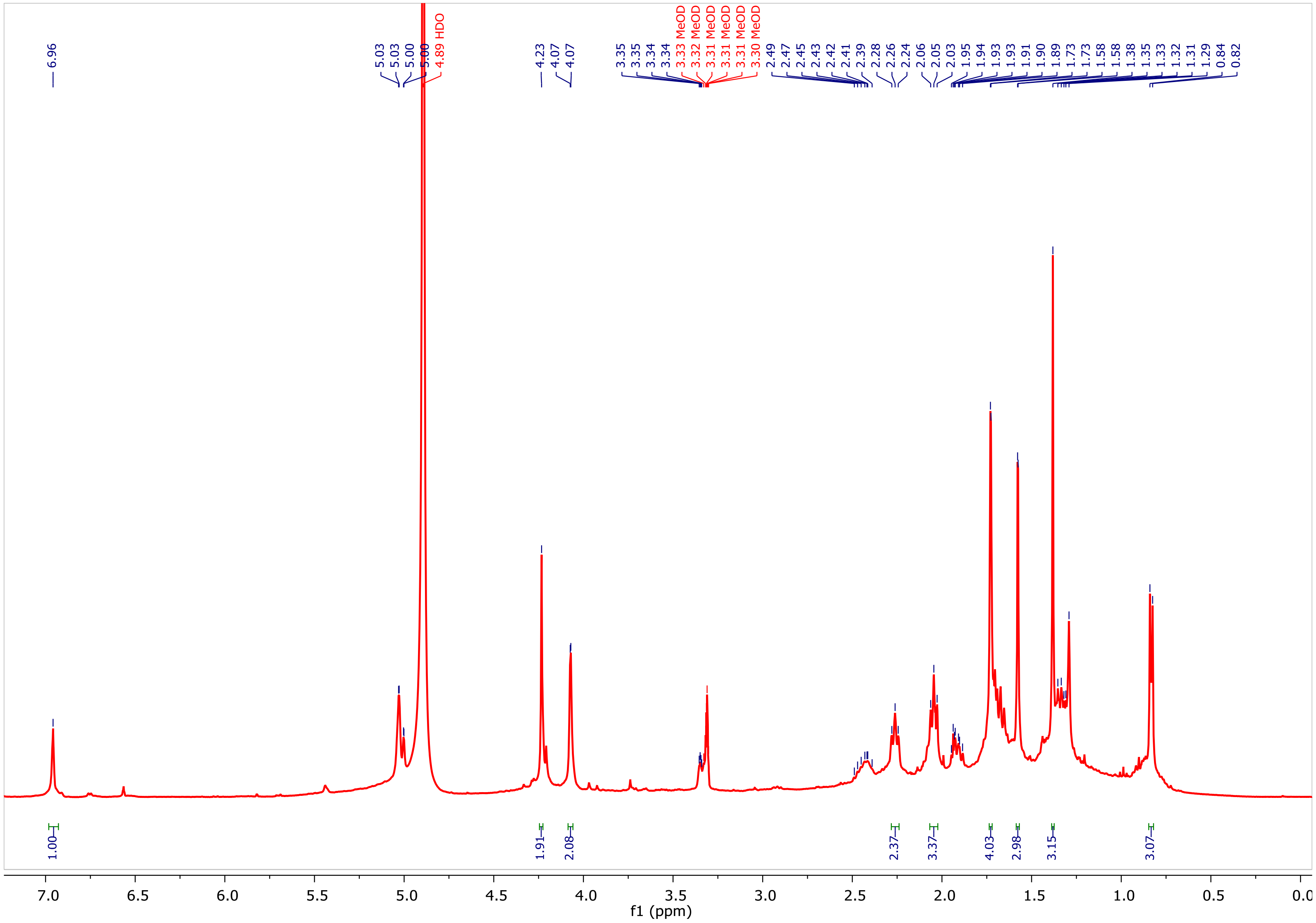

Figure S27. 400 MHz ^{1}H NMR spectrum of oxorcinianin lactam A (**5**, 50%-fraction) in d_4-MeOH.

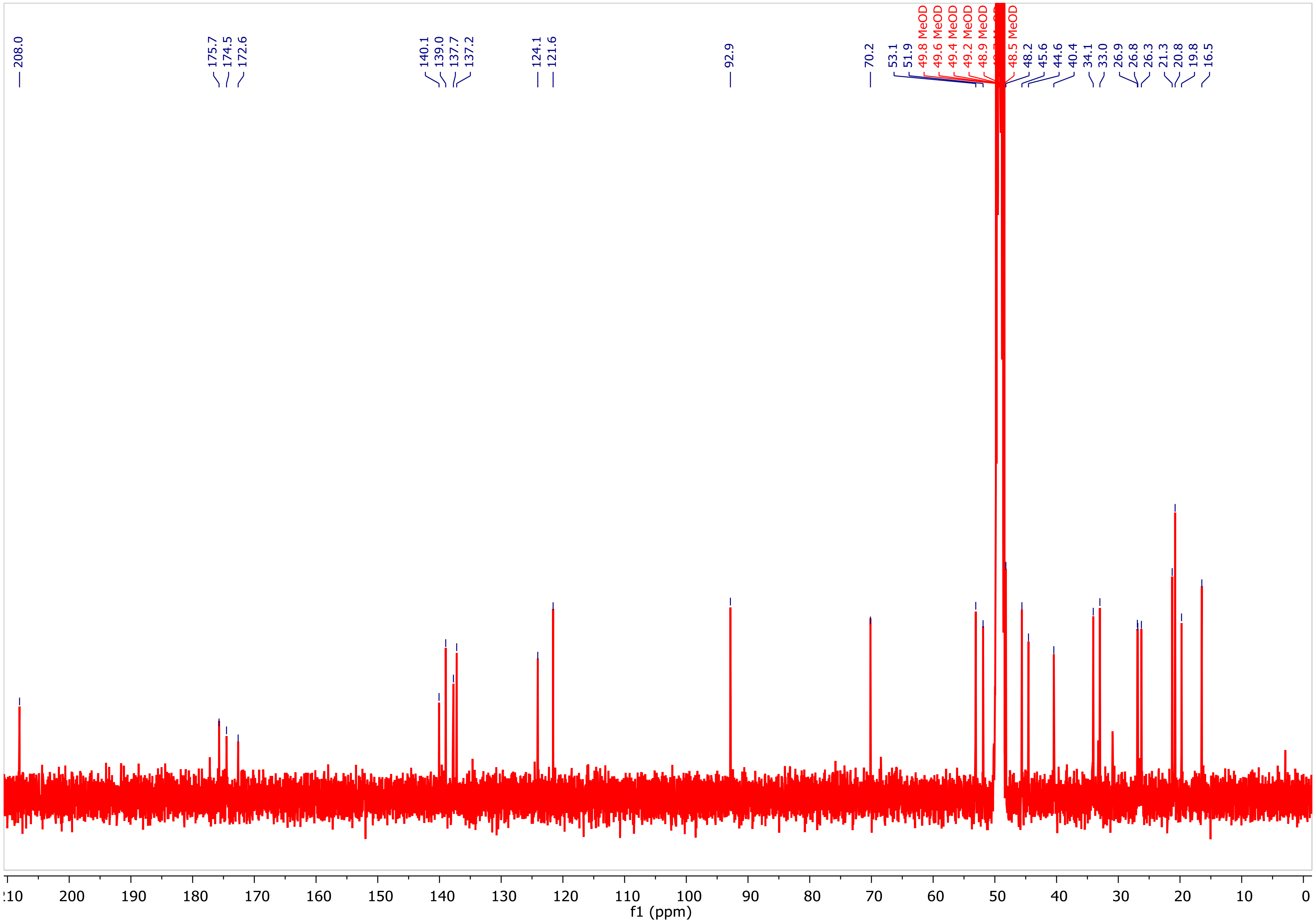

Figure S28. 100 MHz ^{13}C NMR spectrum of oxoircinianin lactam A (**5**, 50%-fraction) in d_4-MeOH.

A

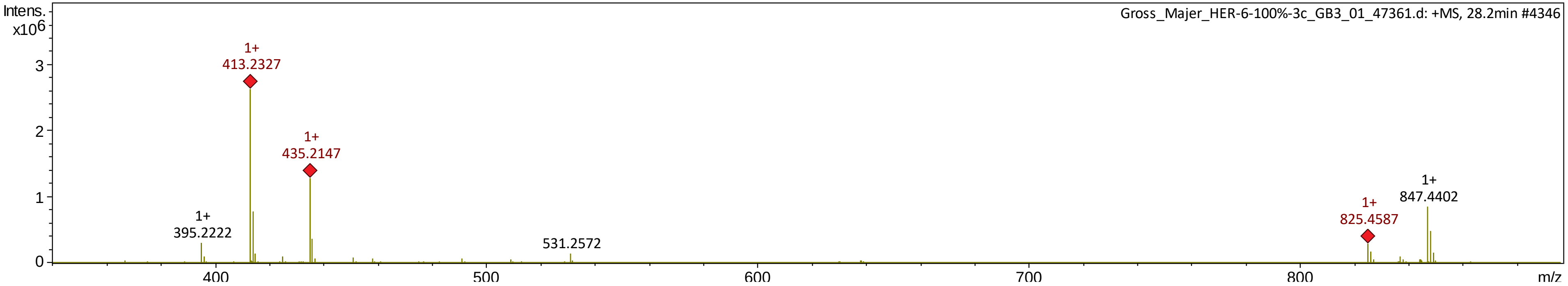

B

Meas. m/z	#	Ion Formula	m/z	err [ppm]	Mean err [ppm]	rdb	N-Rule	e⁻ Conf	mSigma	Std I
413.2327	1	$C_{26}H_{29}N_4O$	413.2336	2.1	1.5	15.0	ok	even	4.6	6.3
413.2327	2	$C_{25}H_{33}O_5$	413.2323	-1.1	-1.4	10.0	ok	even	11.6	17.7
413.2327	3	$C_{21}H_{29}N_6O_3$	413.2296	-7.6	-8.6	11.0	ok	even	25.7	41.2
413.2327	4	$C_{14}H_{33}N_6O_8$	413.2354	6.6	5.1	2.0	ok	even	67.6	109.7
413.2327	5	$C_{11}H_{25}N_{16}O_2$	413.2341	3.3	0.2	8.0	ok	even	68.6	115.8
413.2327	6	$C_{10}H_{29}N_{12}O_6$	413.2328	0.1	-2.4	3.0	ok	even	81.2	136.3
413.2327	7	$C_7H_{21}N_{22}$	413.2314	-3.2	-8.3	9.0	ok	even	82.4	143.0

C

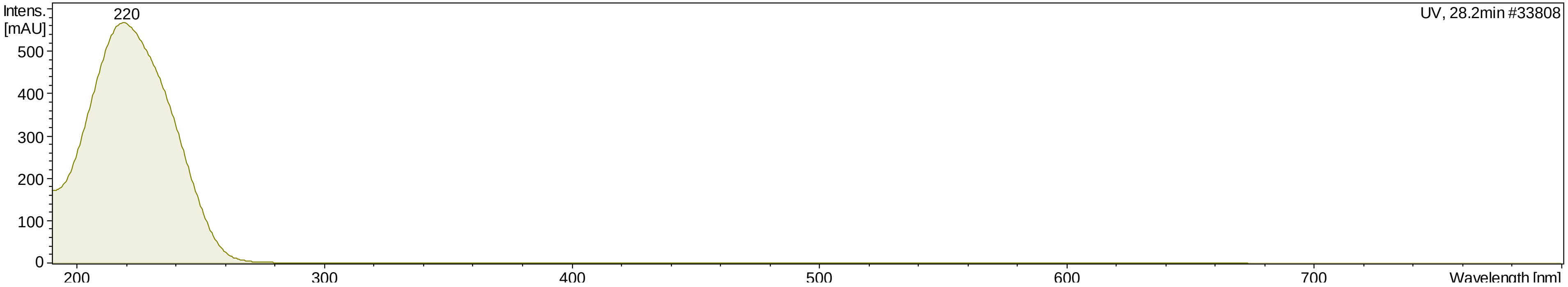

Figure S29. Ircinianin lactone A (**6**, 100%-fraction) MS-Analysis – A: HRMS-result; B: predicted molecular formula; C: extracted UV-Profile.

Table S3. 400 MHz ^{1}H and 100 MHz ^{13}C NMR-data of ircinianin lactone A (**6**) in d_4-MeOH. Chemical shifts are given in ppm.

(-)-Ircinianin lactone A (6)		
**Position**	**δ_H (mult., J [Hz])^a**	**δ_C**
**1**	4.83, br q (1.7)	72.1
**2**	7.37, br quin (1.4)	147.9
**3**	-	134.4
**4**	-	177.0
**5**	2.26, m	25.7
**6**	1.69, m^B	26.7
**7**	2.08, dd (7.6, 7.4)	40.3
**8**	-	136.0
**9**	1.58, d (1.1)	16.1
**10**	5.14, d (10.4)	125.3
**11**	3.07, dm (10.4)	48.6^F
**12**	5.03, m	123.3
**13**	-	137.0
**14**	1.71, d (1.1)^B	20.5^C
**15**	2.41, m	46.0
**16**	*a.* 1.89, m^A<br>*b.* 1.32, m^C	27.1
**17**	*a.* 2.01, m^A<br>*b.* 1.30, m^C	33.5
**18**	1.64, m^E	33.1
**19**	0.92, d (6.1)	20.6^C
**20**	1.61, m*	51.7
**21**	-	86.7
**22**	-	179.3^D
**23**	-	97.3
**24**	-	177.6^D
**25**	1.64, s^E	6.0

[a] Assignments supported by ^{1}H-^{13}C multiplicity edited HSQC.
[A/B/E] Overlapping signals within a column.
[C/D] Assignments interchangeable.
* Overlapped by other signals.
[F] Overlapped by solvent peak.

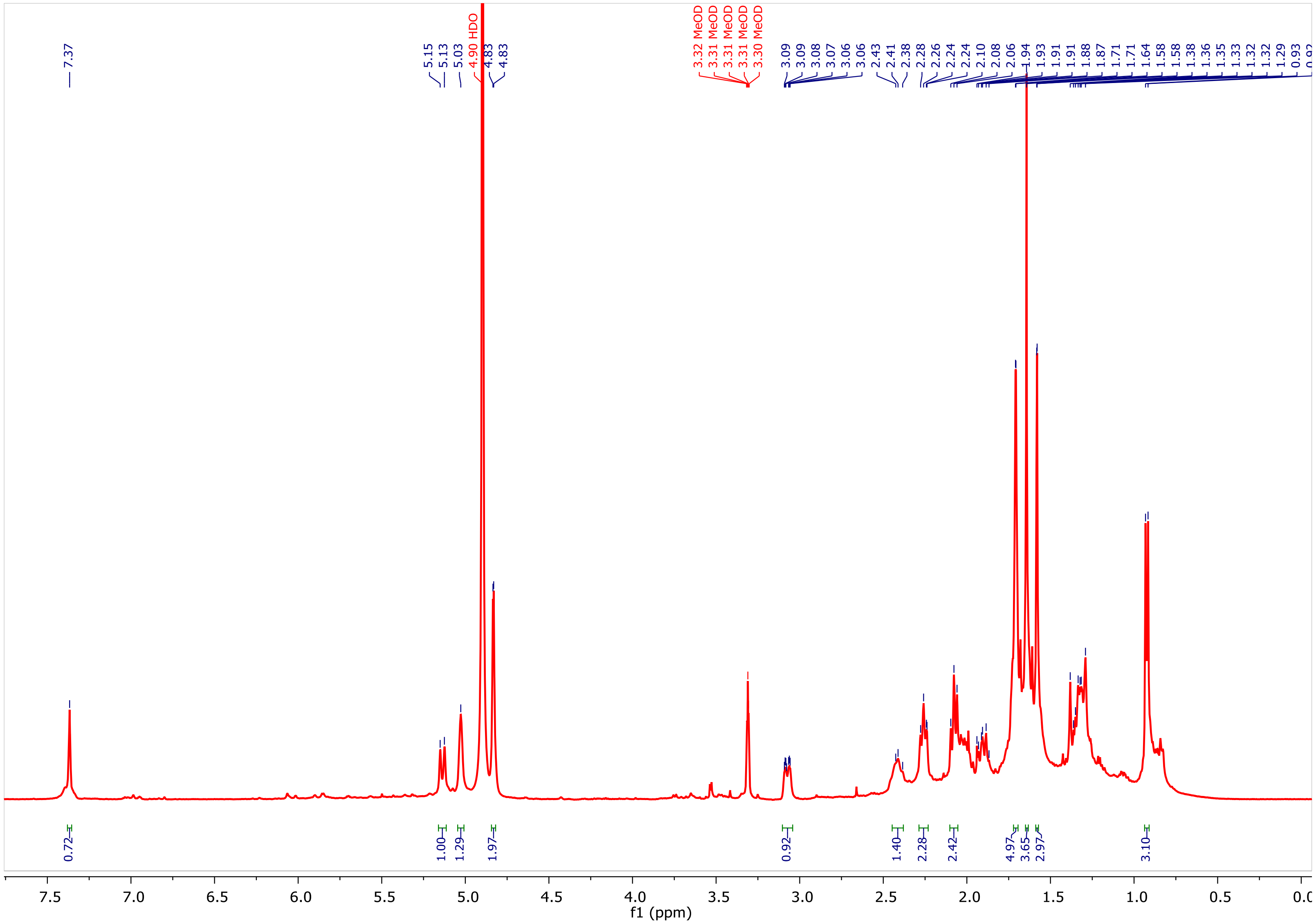

Figure S30. 400 MHz ^{1}H NMR spectrum of ircinianin lactone A (**6**, 100%-fraction) in d_4-MeOH.

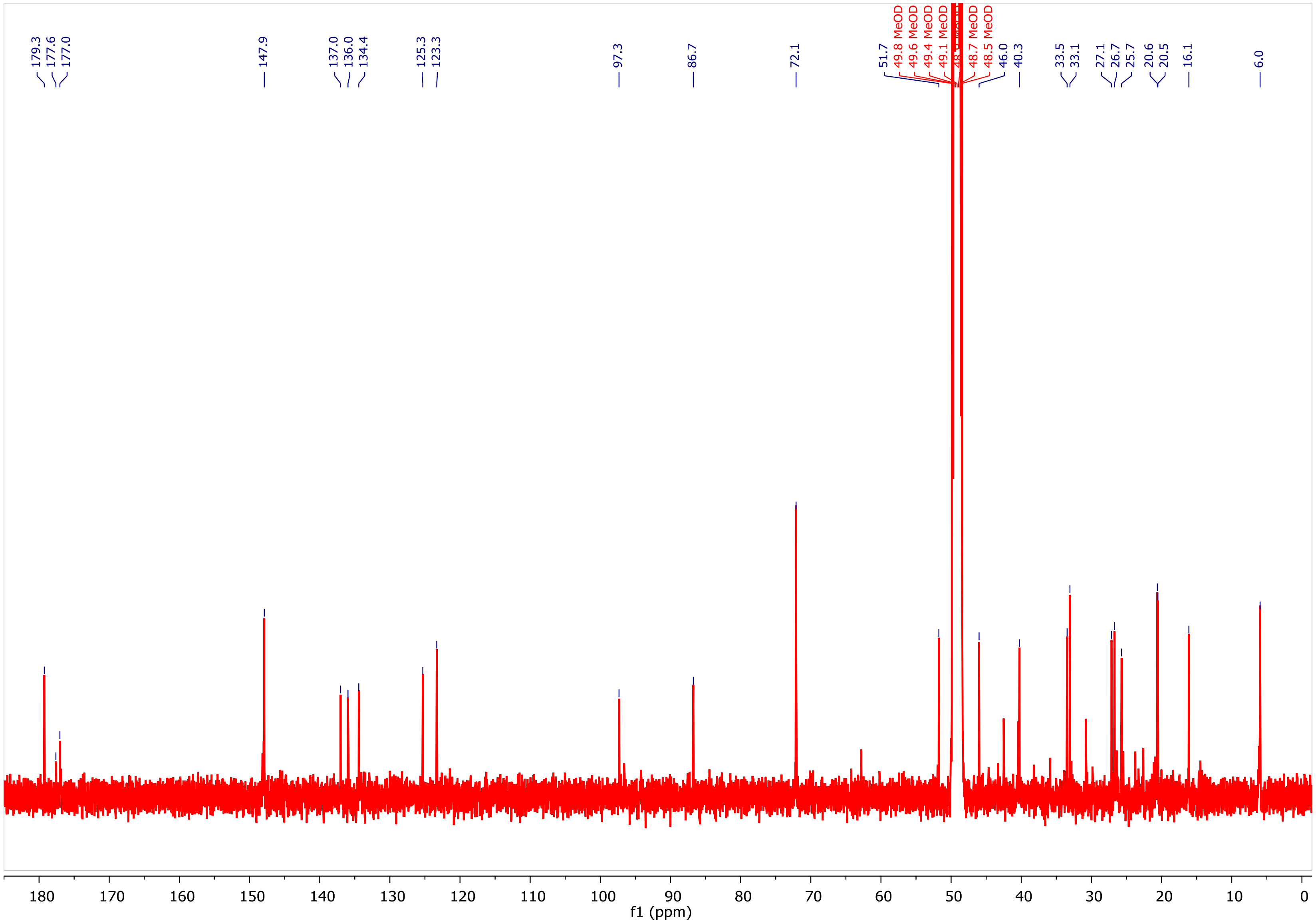

Figure S31. 100 MHz ^{13}C NMR spectrum of ircinianin lactone A (**6**, 100%-fraction) in d_4-MeOH.

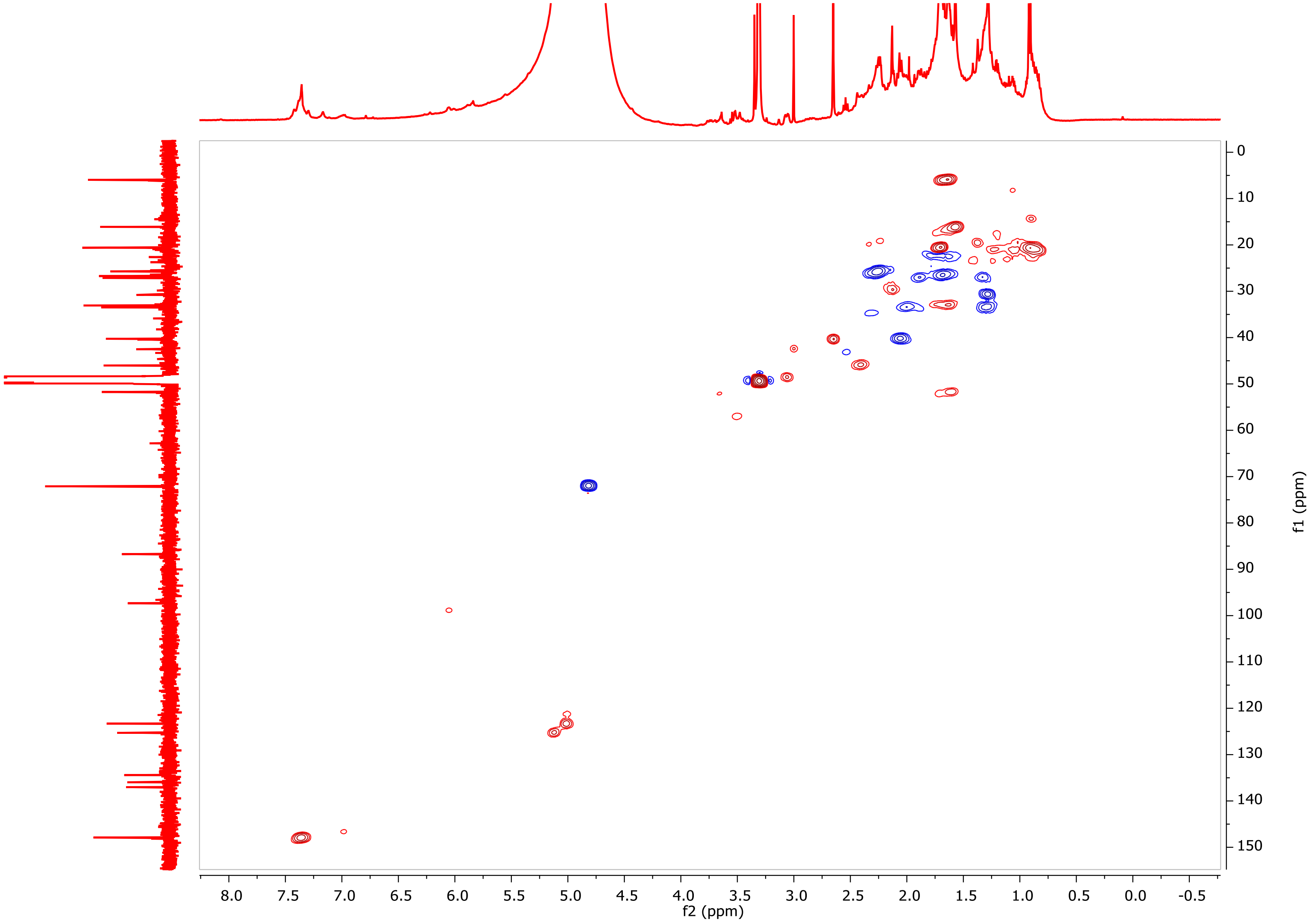

Figure S32. 400 MHz multiplicity edited ^{1}H-^{13}C HSQC NMR spectrum of ircinianin lactone A (**6**, 100%-fraction) in d_4-MeOH.

A

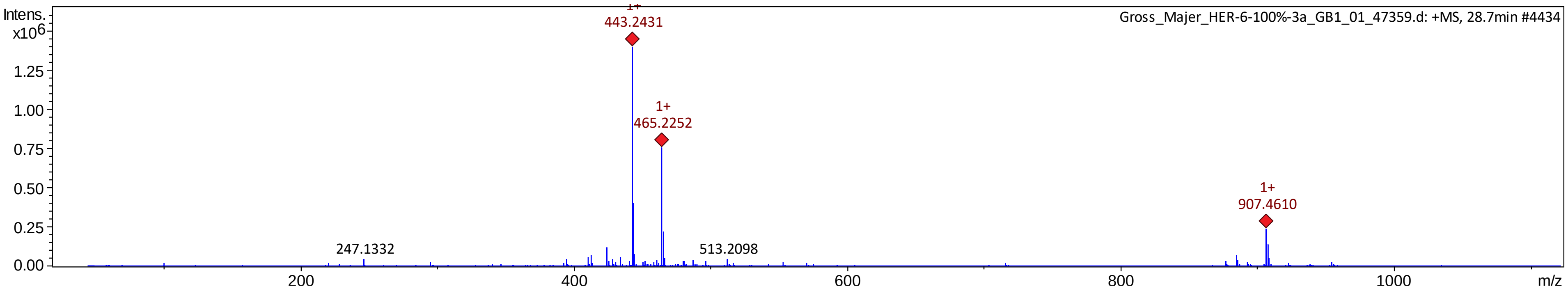

B

Meas. m/z	#	Ion Formula	m/z	err [ppm]	Mean err [ppm]	rdb	N-Rule	e⁻ Conf	mSigma	Std I
443.2431	1	$C_{26}H_{35}O_6$	443.2428	-0.6	-2.1	10.0	ok	even	3.2	7.1
443.2431	2	$C_{23}H_{27}N_{10}$	443.2415	-3.7	-5.2	16.0	ok	even	10.1	16.3
443.2431	3	$C_{27}H_{31}N_4O_2$	443.2442	2.4	1.5	15.0	ok	even	13.3	17.0
443.2431	4	$C_{22}H_{31}N_6O_4$	443.2401	-6.7	-7.9	11.0	ok	even	16.5	27.6

C

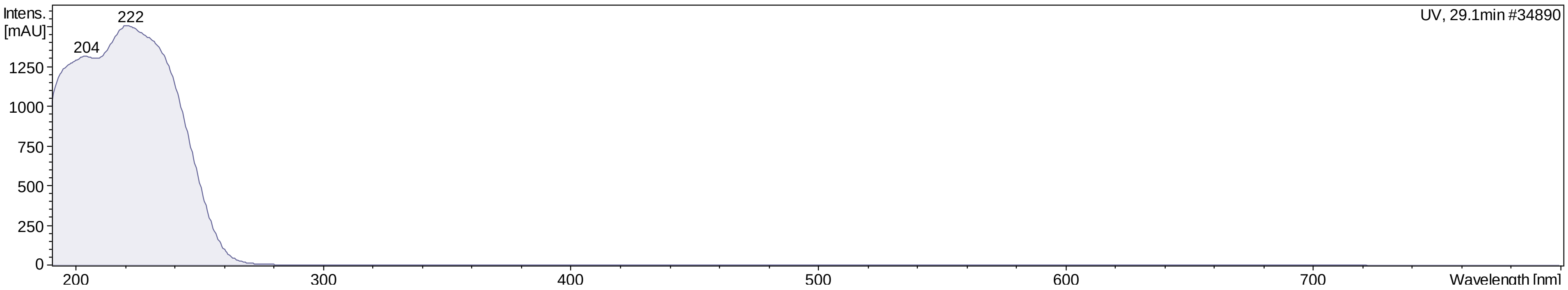

Figure S33. Ircinianin lactone B (**7**), 100%-fraction) MS-Analysis – A: HRMS-result; B: predicted molecular formula; C: extracted UV-Profile.

Table S4. Comparison ^{1}H (400 MHz) and ^{13}C (100 MHz) chemical shift values (ppm) of ircinianin lactone A (**6**) and ircinianin lactone B (**7**), recorded in d_4-MeOH. The column on the far right of the table indicates the shift-deviations in a color-coded way: no deviations; minor to significant shift deviations; strong shift deviations.

Ircinianin lactone A (6)			*Ircinianin lactone B (7)*			Δ
**Position**	**δ_H, mult. (*J* in Hz)**	**δ_C, type**	**Position**	**δ_H, mult. (*J* in Hz)**	**δ_C, type**	**$\lvert \delta_H(6)- \delta_H(7)\rvert$ / $\lvert \delta_C(6)- \delta_C(7)\rvert$**
1	4.83, br q (1.7)	72.1, CH_2	1	5.84, br q (1.2)	104.4, CH	1.01 / 32.3
2	7.37, br quin (1.4)	147.9, CH	2	6.98, br quin (1.2)	144.65; 144.72, CH	0.39 / 3.18 - 3.25
3	-	134.4, C	3	-	139.03; 139.08, C	- / 4.63 - 4.68
4	-	177.0, C	4	-	173.5, C	- / 3.5
5	2.26, m	25.7, CH_2	5	2.26, br t (7.8)	25.5, CH_2	0.00 / 0.2
6	1.69, m	26.7, CH_2	6	1.67, m	26.44; 26.47, CH_2	0.02 / 0.23 - 0.26
7	2.08, dd (7.6, 7.4)	40.3, CH_2	7	2.07, dd (7.9, 7.2)	40.2, CH_2	0.01 / 0.1
8	-	136.0, C	8	-	135.75; 135.78, C	- / 0.22 - 0.25
9	1.58, d (1.1)	16.1, CH_3	9	1.57, d (1.3)	16.06; 16.08, CH_3	0.01 / 0.02 – 0.04
10	5.14, d (10.4)	125.3, CH	10	5.12, m	125.41; 125.44, CH	0.02 / 0.11 – 0.14
11	3.07, dm (10.4)	48.6, CH	11	3.06, dm (10.3)	48.6, CH	0.01 / 0.0
12	5.03, m	123.3, CH	12	5.01, m	123.3, CH	0.02 / 0.0
13	-	137.0, C	13	-	137.0, C	- / 0.0
14	1.71, d (1.1)	20.5, CH_3	14	1.70, d (1.4)	20.56, CH_3	0.01 / 0.06
15	2.41, m	46.0, CH	15	2.40, m	46.0, CH	0.01 / 0.0
16	1.89, m; 1.32, m	27.1, CH_2	16	1.89, m; 1.32, m	27.1, CH_2	0.00; 0.00 / 0.0
17	2.01, m; 1.30, m	33.5, CH_2	17	2.00, m; 1.29, m	33.4, CH_2	0.01; 0.01 / 0.1
18	1.64, m	33.1, CH	18	1.63, m	33.1, CH	0.01 / 0.0
19	0.92, d (6.1)	20.6, CH_3	19	0.92, d (6.2)	20.62, CH_3	0.00 / 0.02
20	1.61, m	51.7, CH	20	1.61, m	51.7, CH	0.00 / 0.0
21	-	86.7, C	21	-	86.7, C	- / 0.0
22	-	179.3, C	22	-	179.3, C	- / 0.0
23	-	97.3, C	23	-	97.3, C	- / 0.0
24	-	177.6, C	24	-	177.6, C	- / 0.0
25	1.64, s	6.0, CH_3	25	1.63, br s	6.0, CH_3	0.01 / 0.0
1´	-	-	1´	3.52, br s	57.13; 57.16, CH_3	3.52 / 57.13 -57.16

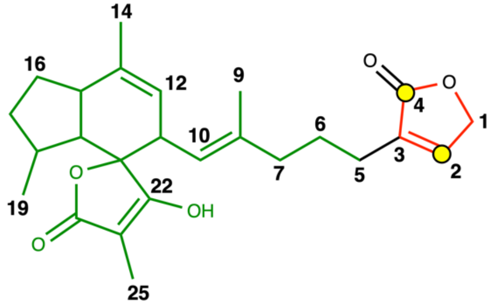

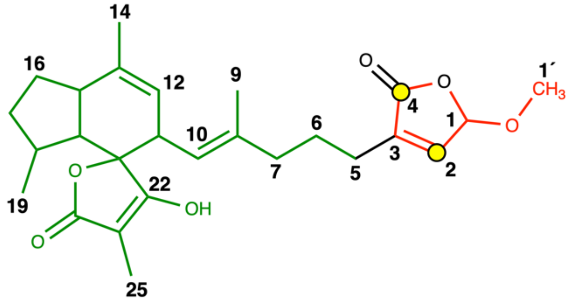

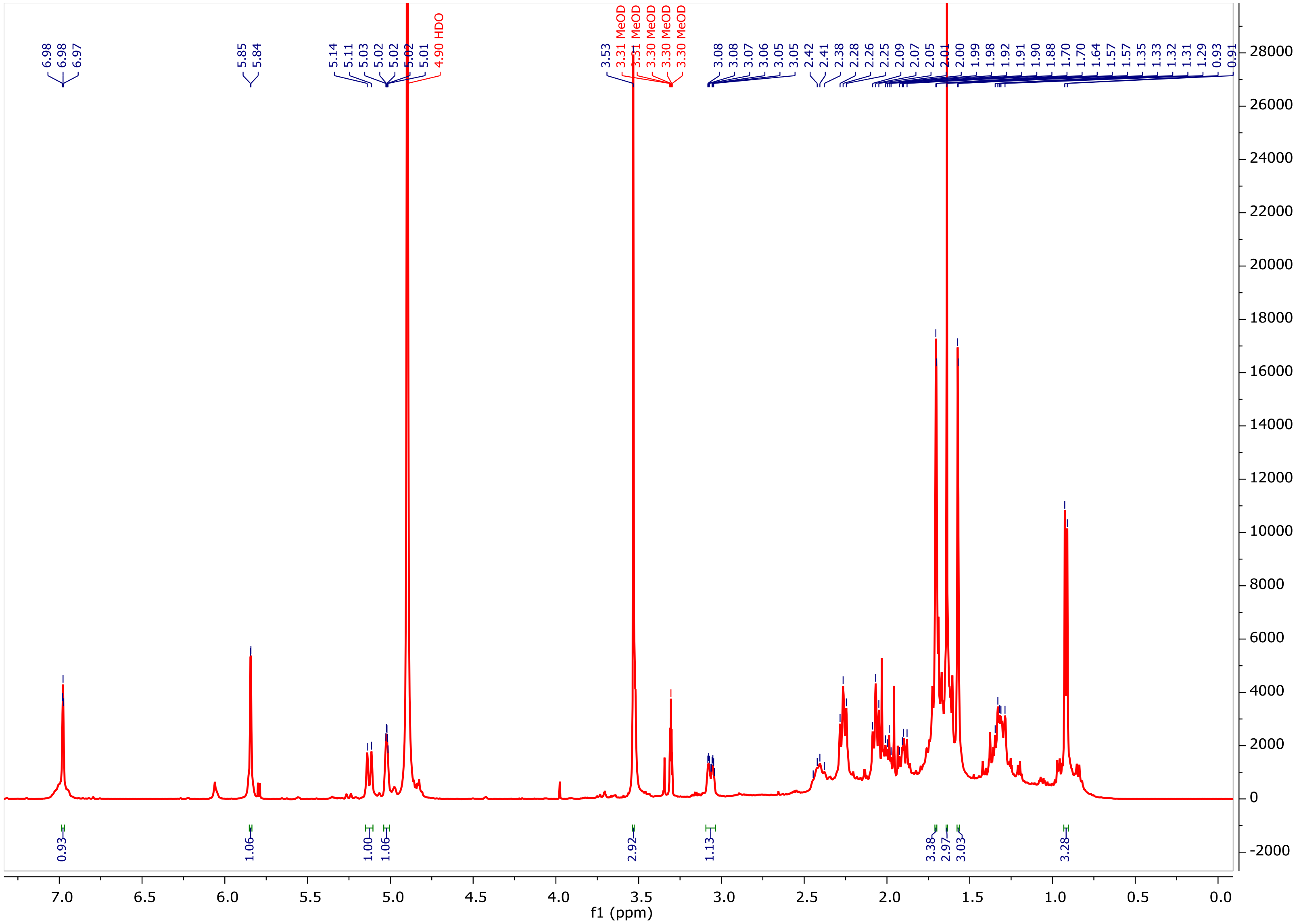

Figure S34. 400 MHz ^{1}H NMR spectrum of ircinianin lactone B (**7**, 100%-fraction) in d_4-MeOH.

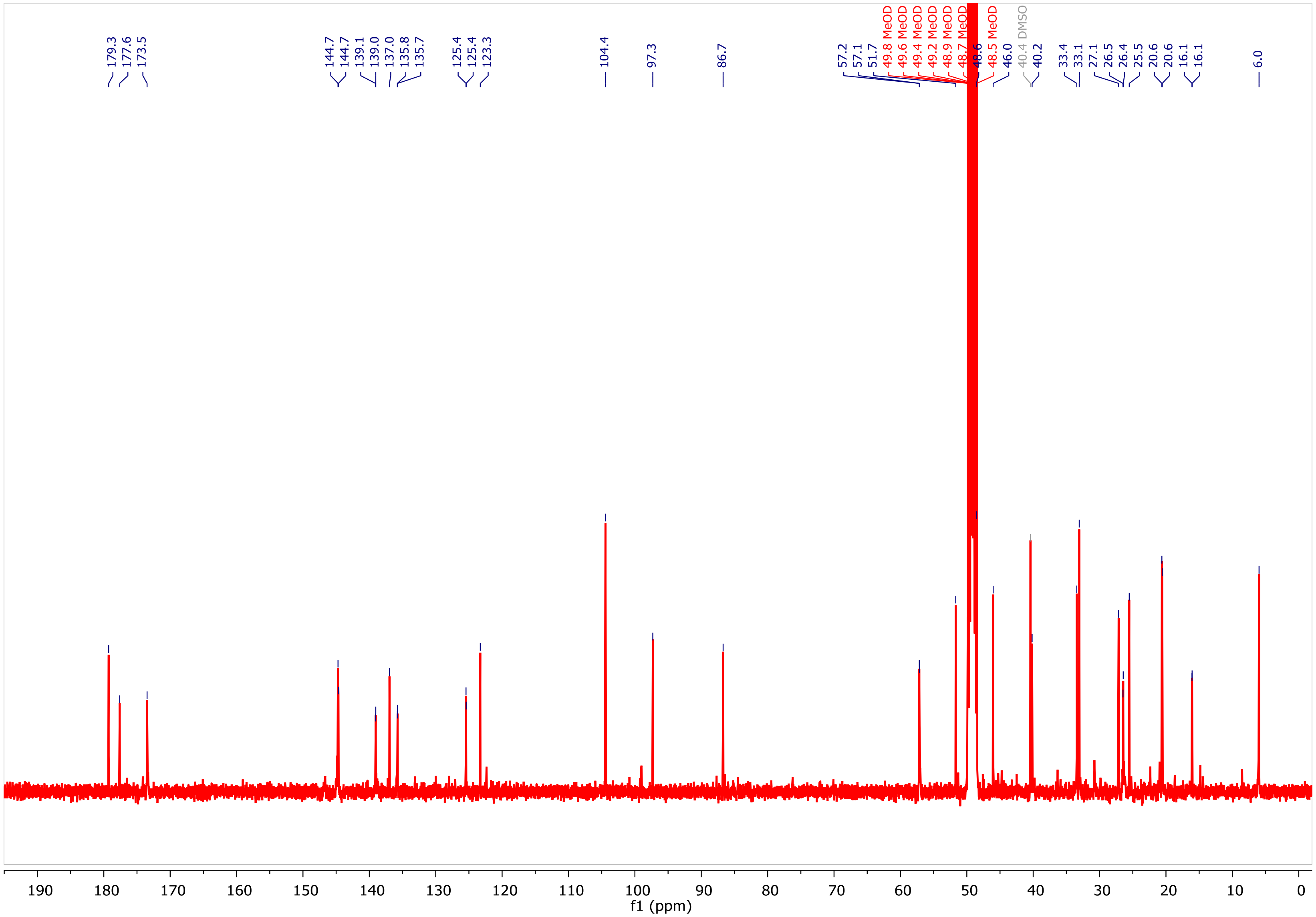

Figure S35. 100 MHz ^{13}C NMR spectrum of ircinianin lactone B (**7**, 100%-fraction) in d_4-MeOH.

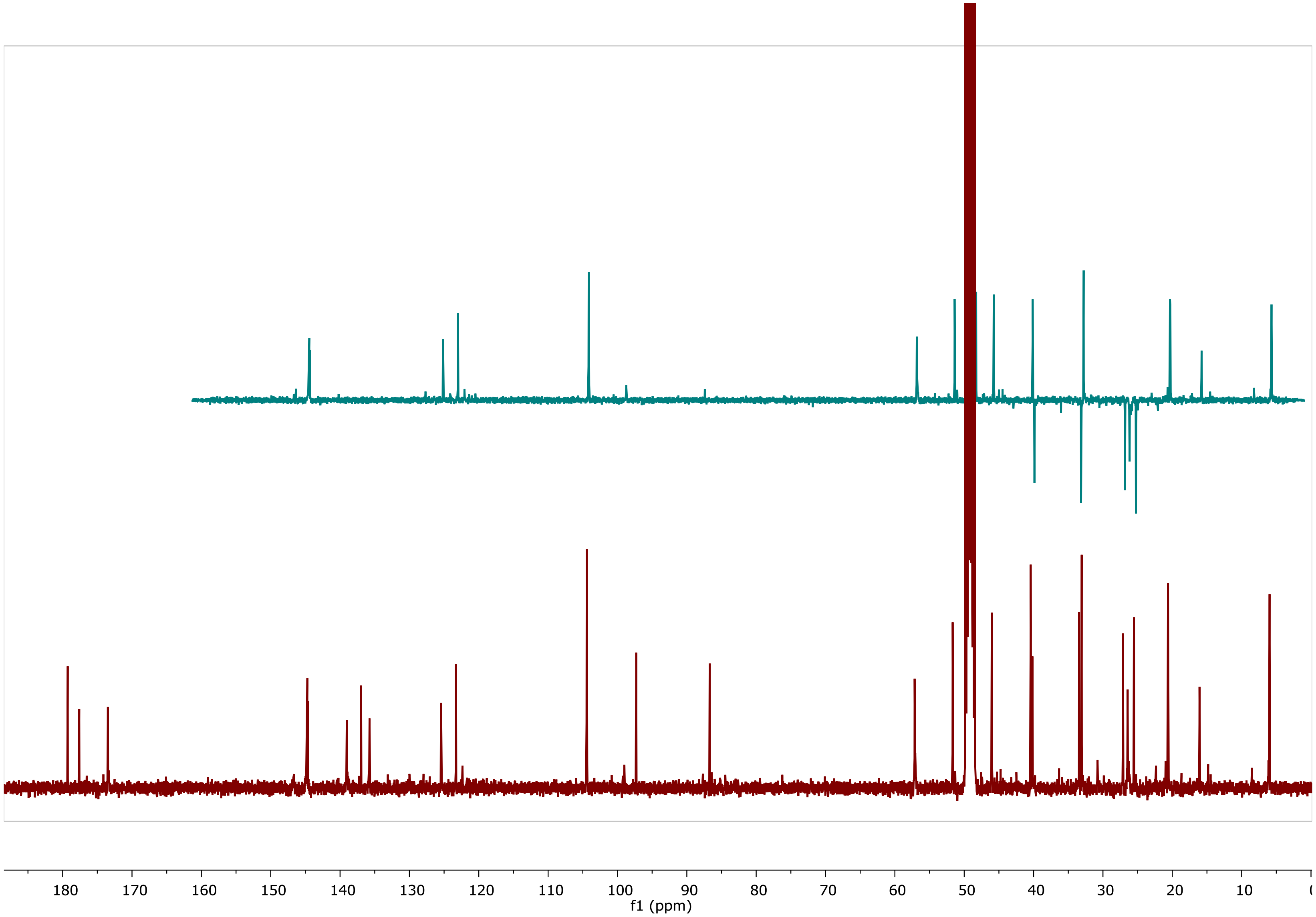

Figure S36. 100 MHz ^{13}C (bottom) and 400 MHz DEPT135 (top) NMR spectrum of ircinianin lactone B (**7**, 100%-fraction) in d_4-MeOH.

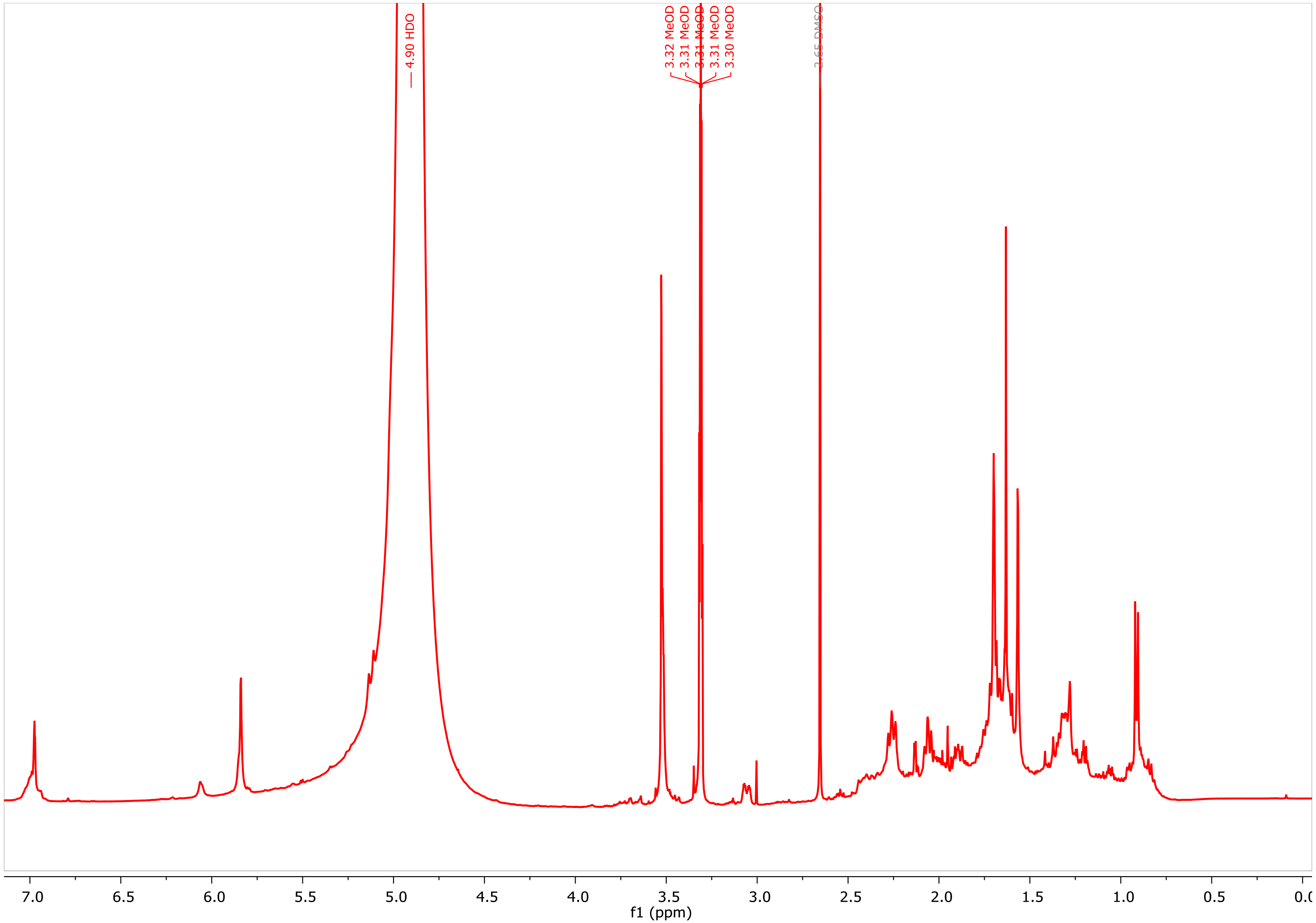

Figure S37. 400 MHz ^{1}H NMR spectrum of ircinianin lactone B (**7**, 100%-fraction) in d_4-MeOH (before 2D NMR experiments).

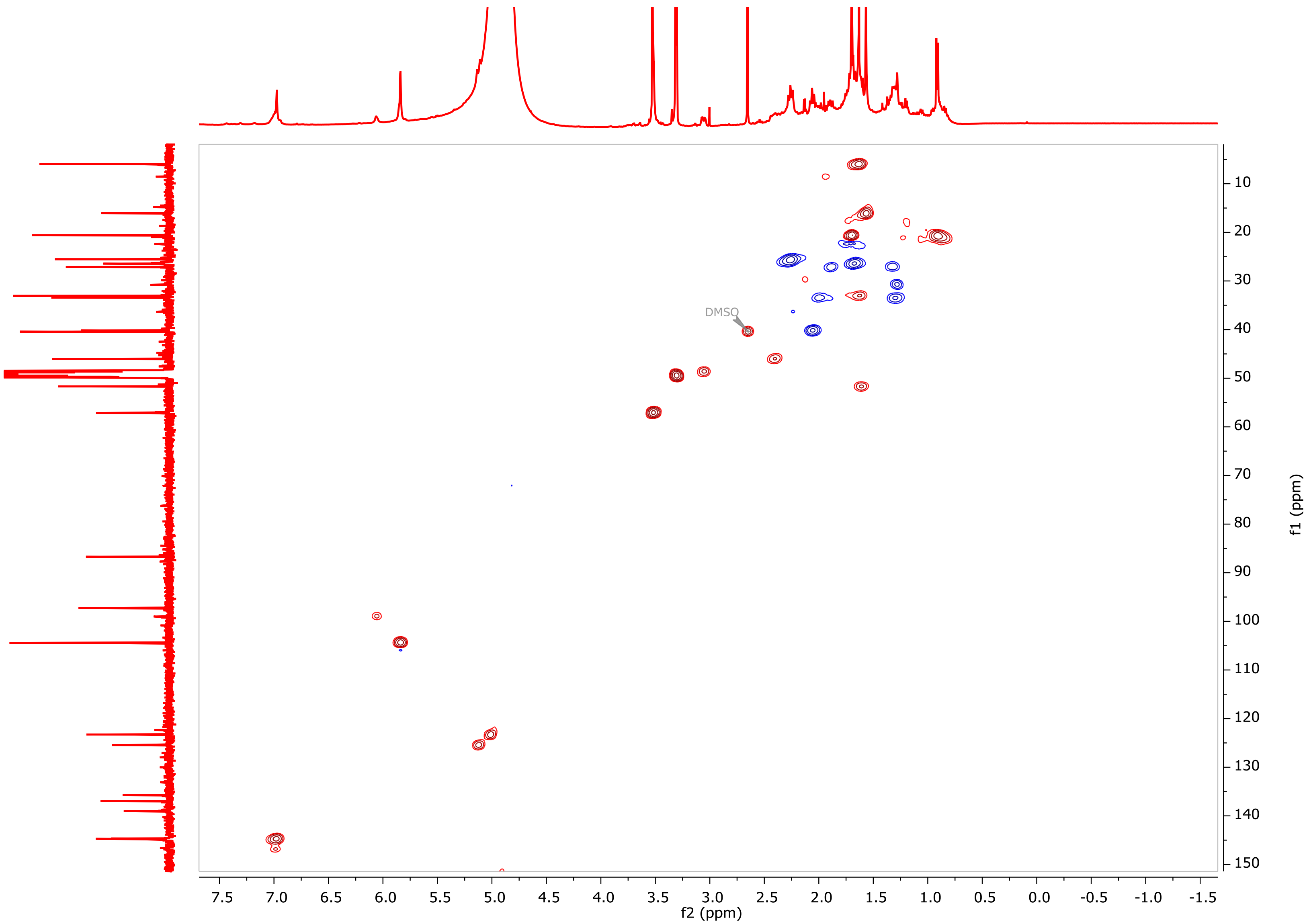

Figure S38. 400 MHz multiplicity edited ^{1}H-^{13}C HSQC NMR spectrum of ircinianin lactone B (**7**, 100%-fraction) in d_4-MeOH.

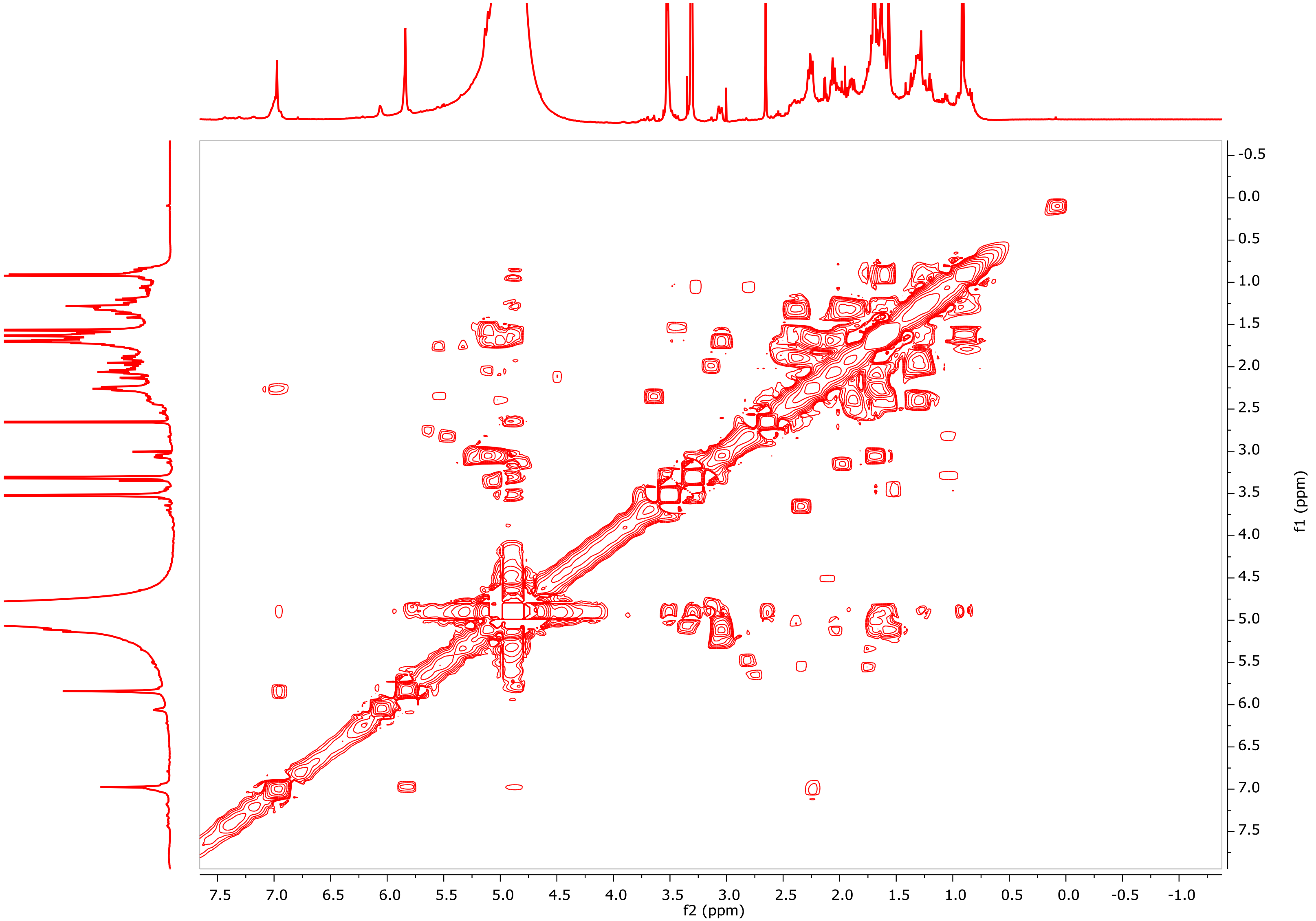

Figure S39. 400 MHz ^{1}H-^{1}H COSY NMR spectrum of ircinianin lactone B (**7**, 100%-fraction) in d_4-MeOH.

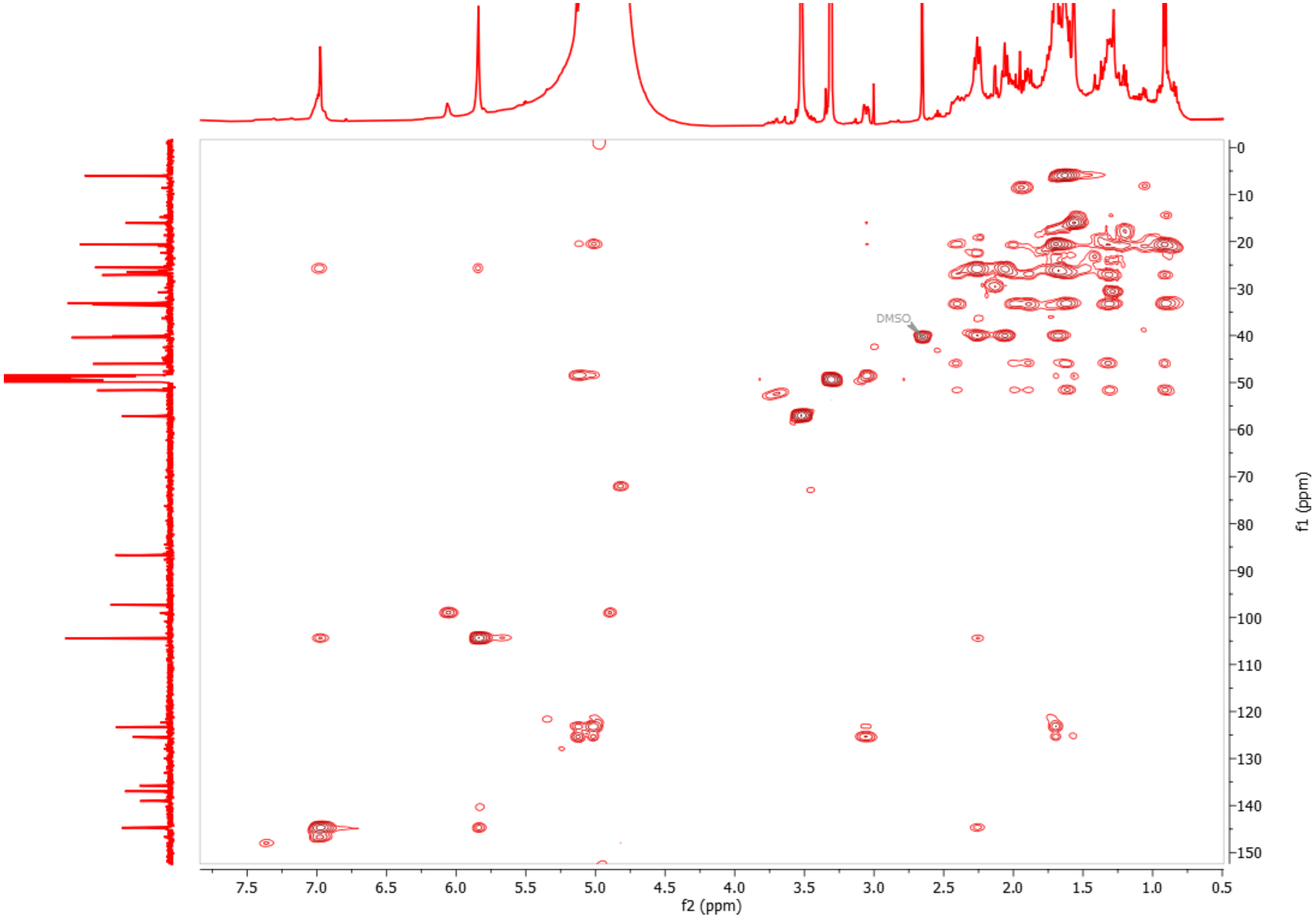

Figure S40. 400 MHz ^{1}H-^{13}C HSQC-TOCSY NMR spectrum of ircinianin lactone B (**7**, 100%-fraction) in *d*$_4$-MeOH.

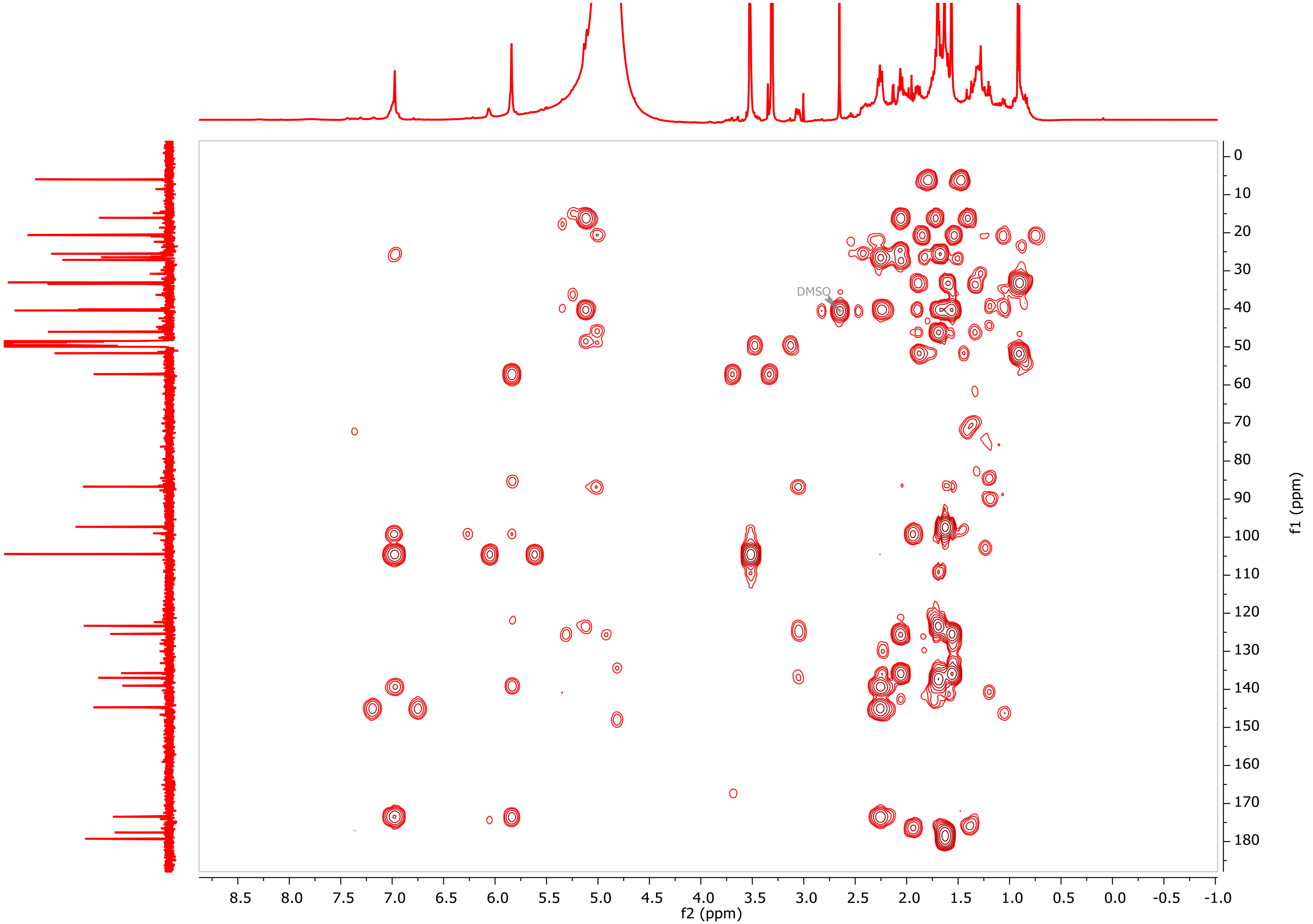

Figure S41. 400 MHz ^{1}H-^{13}C HMBC NMR spectrum of ircinianin lactone B (**7**, 100%-fraction) in d_4-MeOH.

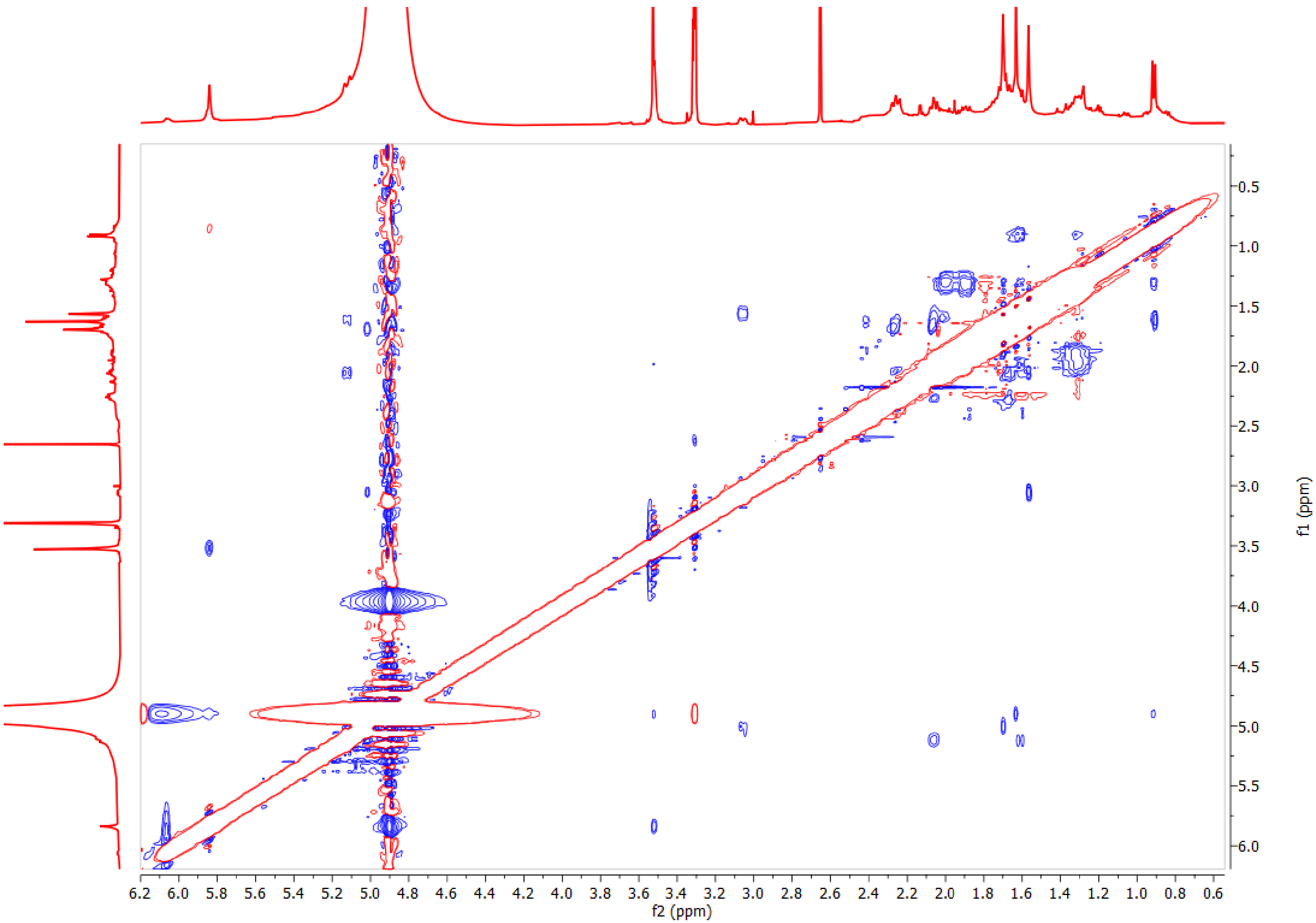

Figure S42. 400 MHz ^{1}H-^{1}H NOESY NMR spectrum of ircinianin lactone B (**7**, 100%-fraction) in d_4-MeOH.

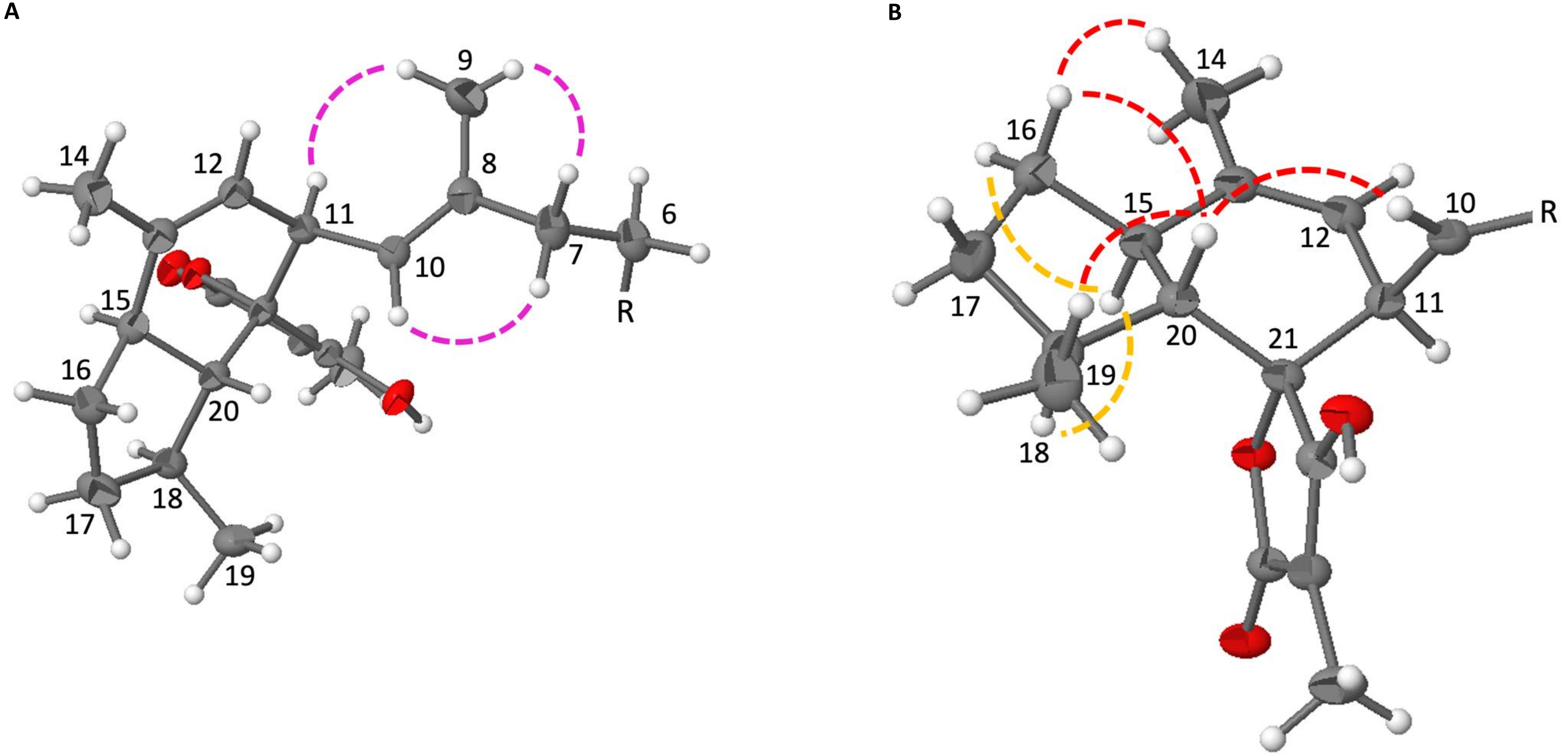

Figure S43. Visualization of the key cross peaks, observed in the ^{1}H-^{1}H NOESY NMR spectrum of **7**.
A) Key-NOE contacts, drawn in magenta dashed lines, which supported the deduction of the *E*-double bond geometry of $^{8,10}\Delta$, located in the alkene side chain.
B) Key-NOE contacts, that allowed the deduction of the relative configuration of atoms C-11, C15, C-18 and C-20. Red dashed lines indicate NOE contacts on the upper side of the molecule, while orange dashed lines represent the NOE correlations on the lower side of the molecule.

A

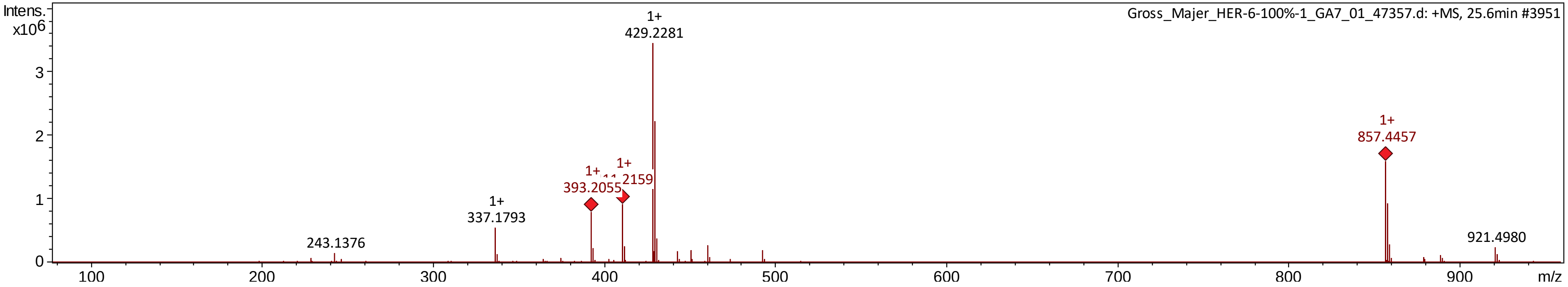

B

Meas. m/z	#	Ion Formula	m/z	err [ppm]	Mean err [ppm]	rdb	N-Rule	e⁻ Conf	mSigma	Std I
429.2281	1	$C_{25}H_{33}O_6$	429.2272	-2.3	-0.1	10.0	ok	even	185.1	242.6
429.2281	2	$C_{26}H_{29}N_4O_2$	429.2285	0.8	2.4	15.0	ok	even	200.5	225.2
429.2281	3	$C_{22}H_{25}N_{10}$	429.2258	-5.4	-4.7	16.0	ok	even	214.4	247.7
429.2281	4	$C_{21}H_{29}N_6O_4$	429.2245	-8.5	-7.5	11.0	ok	even	227.5	266.3
429.2281	5	$C_{15}H_{29}N_{10}O_5$	429.2317	8.3	8.5	7.0	ok	even	256.8	314.9
429.2281	6	$C_{14}H_{33}N_6O_9$	429.2304	5.1	5.8	2.0	ok	even	269.7	335.1
429.2281	7	$C_{11}H_{25}N_{16}O_3$	429.2290	2.0	1.1	8.0	ok	even	270.5	340.7
429.2281	8	$C_{10}H_{29}N_{12}O_7$	429.2277	-1.1	-1.5	3.0	ok	even	283.3	361.5
429.2281	9	$C_7H_{21}N_{22}O$	429.2263	-4.3	-6.9	9.0	ok	even	284.1	367.6

C

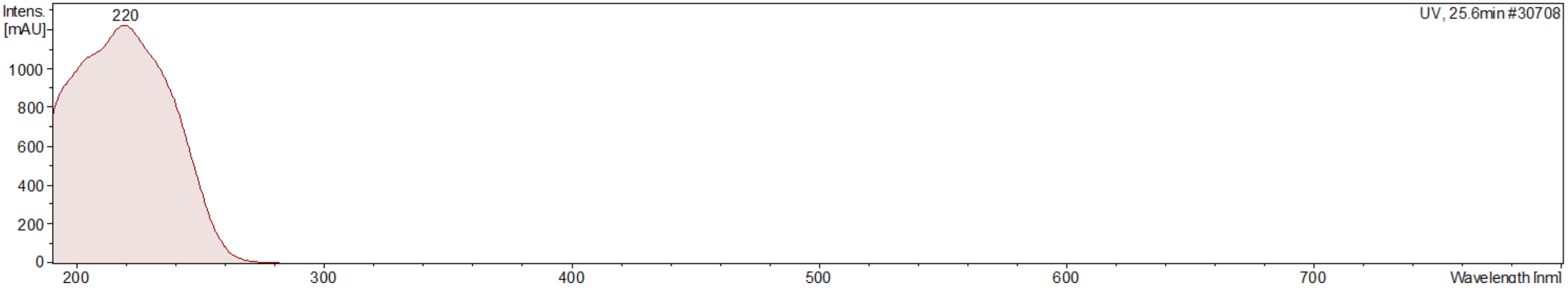

Figure S44. Ircinianin lactone C (**8**, 100%-fraction) MS-Analysis – A: HRMS-result; B: predicted molecular formula; C: extracted UV-Profile.

Table S5. Comparison ^{1}H (400 MHz) and ^{13}C (100 MHz) chemical shift values (ppm) of ircinianin (**1**) and ircinianin lactone C (**8**), recorded in d_4-MeOH. The column on the far right of the table indicates the shift-deviations in a color-coded way: no deviations; minor to significant shift deviations; strong shift deviations. Please note the different numbering schemes for the 5-membered ring systems (furan vs. ß-substituted γ-hydroxy-γ-butenolide ring).

Ircinianin (1)			*Ircinianin lactone C (8)*			Δ
**Position**	**δ_H, mult. (*J* in Hz)**	**δ_C, type**	**Position**	**δ_H, mult. (*J* in Hz)**	**δ_C, type**	**$\lvert\delta_H(1)-\delta_H(8)\rvert$ / $\lvert\delta_C(1)-\delta_C(8)\rvert$**
**1**	7.38, t (1.7)	144.0, CH	**4**	-	173.8, C	7.38 / 29.8
**2**	6.30, d (0.9)	112.1, CH	**3**	5.90, d (2.3)	117.9, CH	0.40 / 5.8
**3**	-	126.5, C	**2**	-	172.7, C	- / 46.2
**4**	7.26, m	140.3, CH	**1**	6.02, d (2.3)	101.1, CH	1.24 / 39.2
5	2.41, br t (7.5)	25.3, CH$_2$	5	2.43, m	28.2, CH$_2$	0.02 / 2.9
6	1.68, m	29.5, CH$_2$	6	1.76, m	25.9, CH$_2$	0.08 / 3.6
7	2.04, m	40.5, CH$_2$	7	2.11, m	40.4, CH$_2$	0.07 / 0.1
8	-	136.6, C	8	-	135.9, C	- / 0.3
9	1.57, d (1.3)	16.3, CH$_3$	9	1.59, d	16.2, CH$_3$	0.02 / 0.1
10	5.11, dd (10.3, 1.1)	125.0, CH	10	5.15, d (10.3)	125.7, CH	0.04 / 0.7
11	3.08, dm (10.3)	48.7, CH	11	3.08, dm (10.1)	48.7, CH	0.00 / 0.0
12	5.03, m	123.6, CH	12	5.03, m	123.4, CH	0.00 / 0.2
13	-	137.1, C	13	-	137.2, C	- / 0.1
14	1.71, m	20.8, CH$_3$	14	1.71, m	20.68, CH$_3$	0.00 / 0.12
15	2.42, m	46.2, CH	15	2.42, m	46.2, CH	0.00 / 0.0
16	1.89, m; 1.34, m	27.3, CH$_2$	16	1.89, m; 1.34, m	27.3, CH$_2$	0.00; 0.00 / 0.0
17	2.00, m; 1.32, m	33.6, CH$_2$	17	2.02, m; 1.31, m	33.6, CH$_2$	0.02; 0.01 / 0.0
18	1.65, m	33.2, CH	18	1.64, m	33.2, CH	0.01 / 0.0
19	0.92, d (6.3)	20.7, CH$_3$	19	0.93, d (6.2)	20.74, CH$_3$	0.01 / 0.04
20	1.61, m	52.0, CH	20	1.62, m	51.8, CH	0.01 / 0.2
21	-	86.9, C	21	-	86.8, C	- / 0.1
22	-	179.2, C	22	-	179.1, C	- / 0.1
23	-	97.5, C	23	-	97.5, C	- / 0.0
24	-	177.7, C	24	-	177.7, C	- / 0.0
25	1.64, br s	6.1, CH$_3$	25	1.65, br s	6.1, CH$_3$	0.01 / 0.0

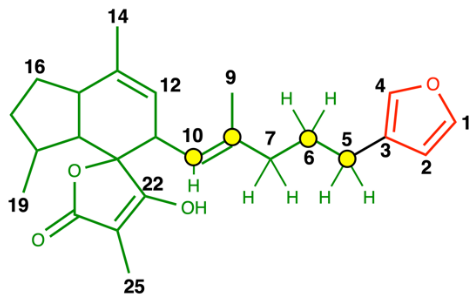

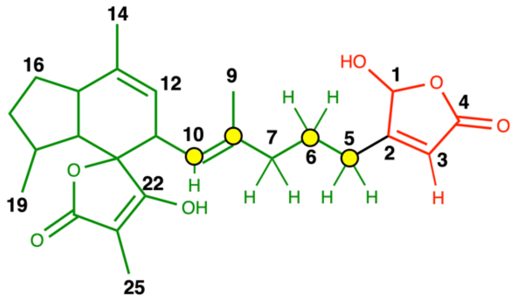

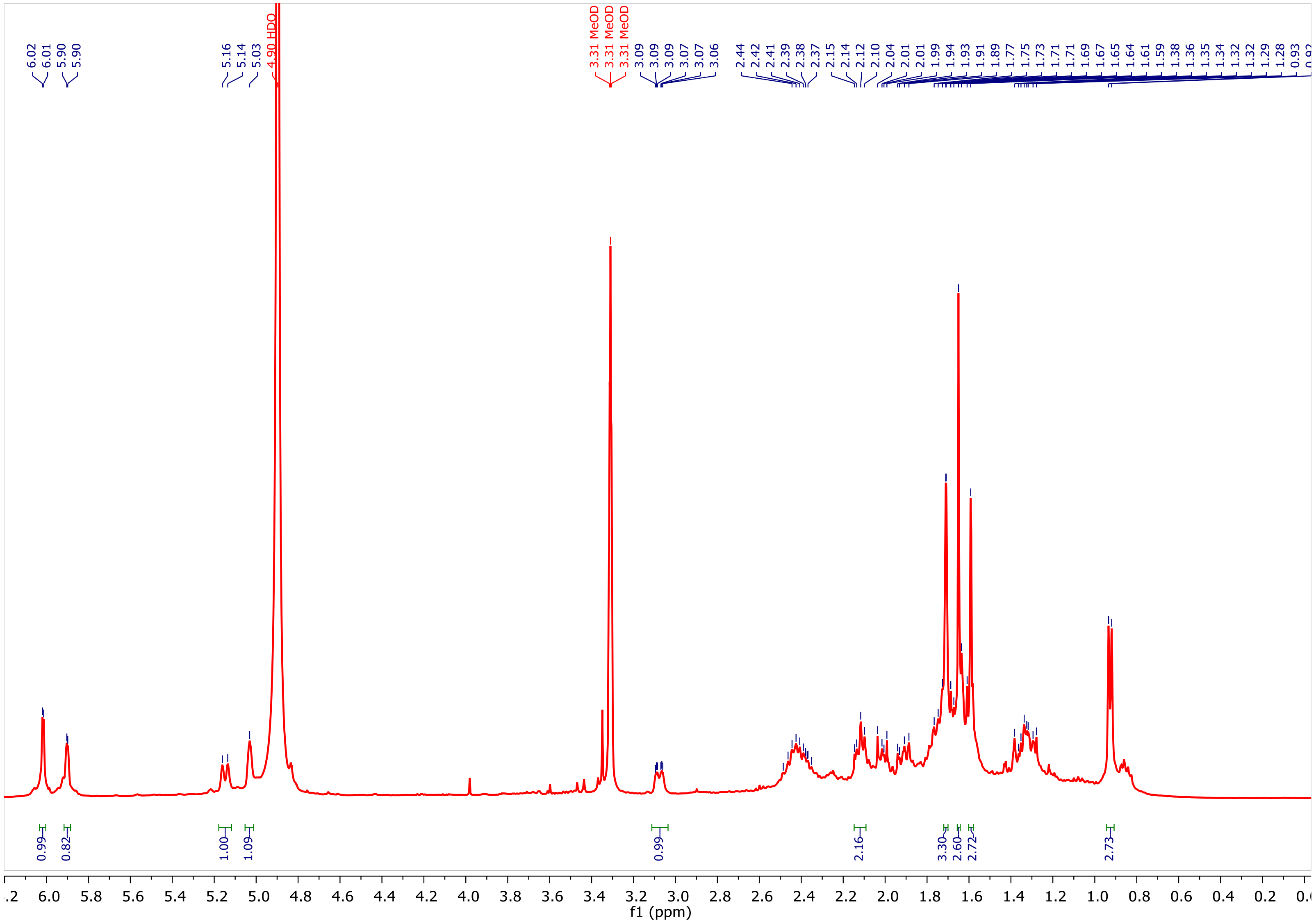

Figure S45. 400 MHz ^{1}H NMR spectrum of ircinianin lactone C (**8**, 100%-fraction) in d_4-MeOH.

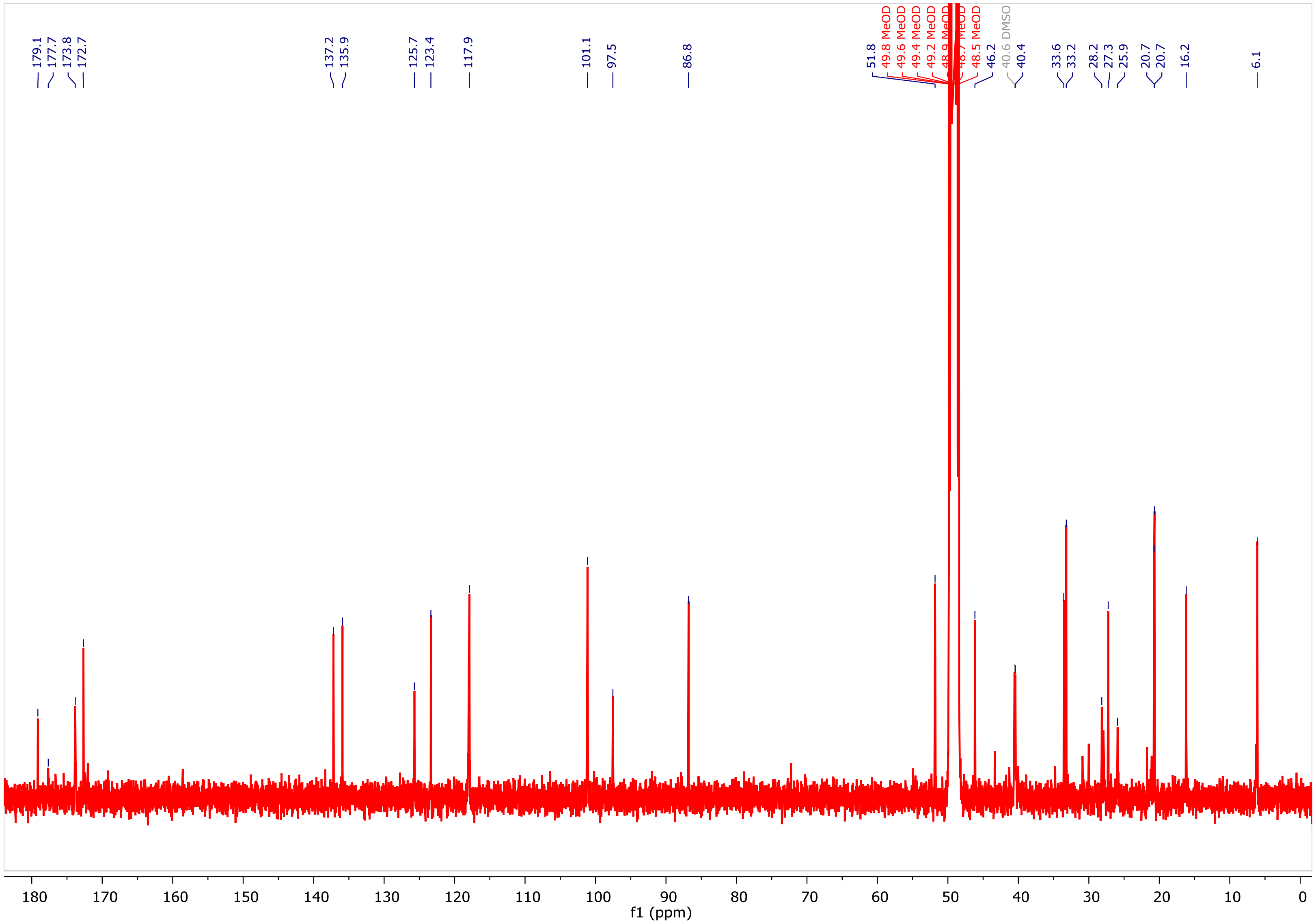

Figure S46. 100 MHz ^{13}C NMR spectrum of ircinianin lactone C (**8**, 100%-fraction) in d_4-MeOH.

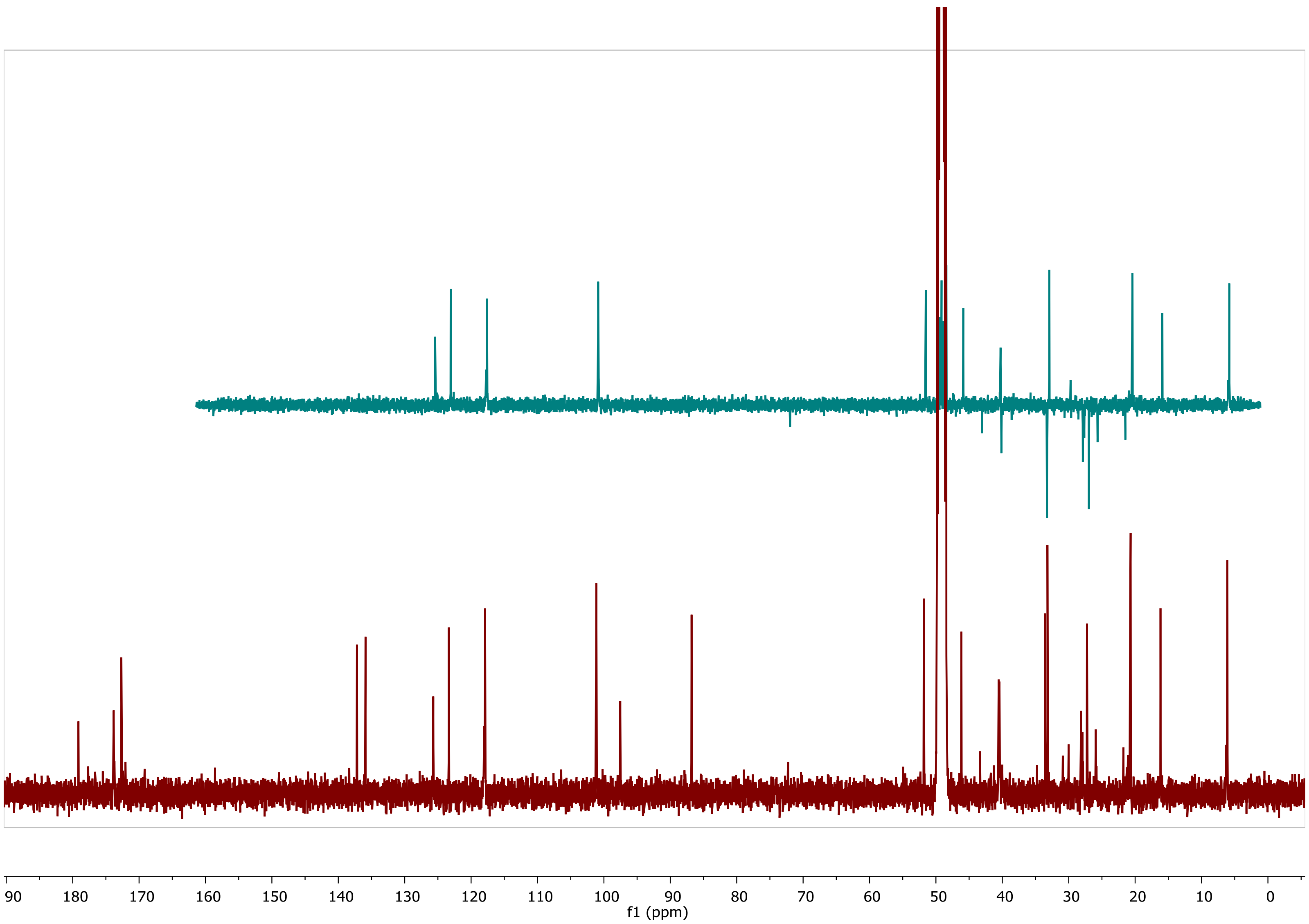

Figure S47. 100 MHz ^{13}C (bottom) and 400 MHz DEPT135 (top) NMR spectrum of ircinianin lactone C (**8**, 100%-fraction) in d_4-MeOH.

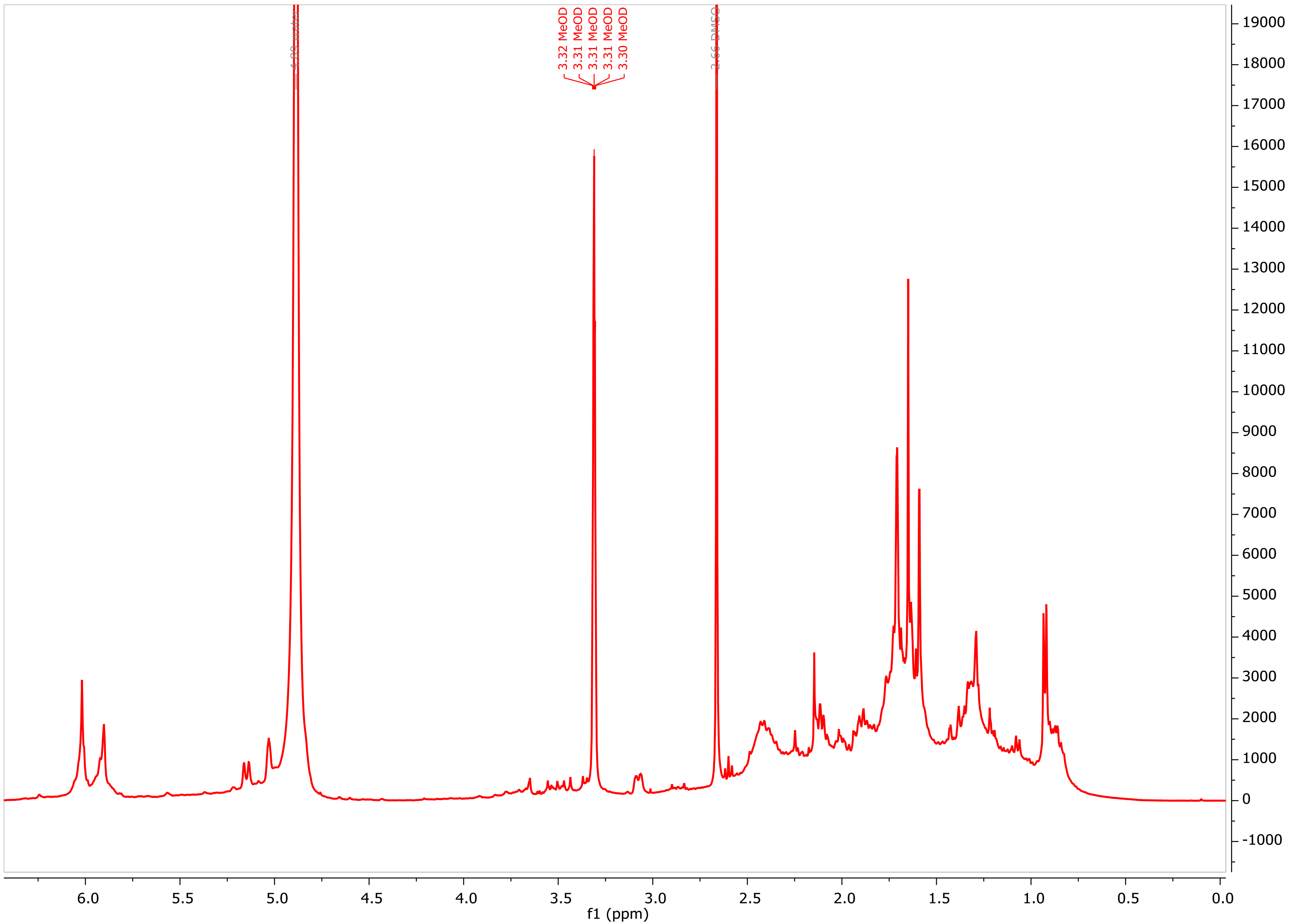

Figure S48. 400 MHz ^{1}H NMR spectrum of ircinianin lactone C (**8**, 100%-fraction) in d_4-MeOH before 2D-NMR experiments.

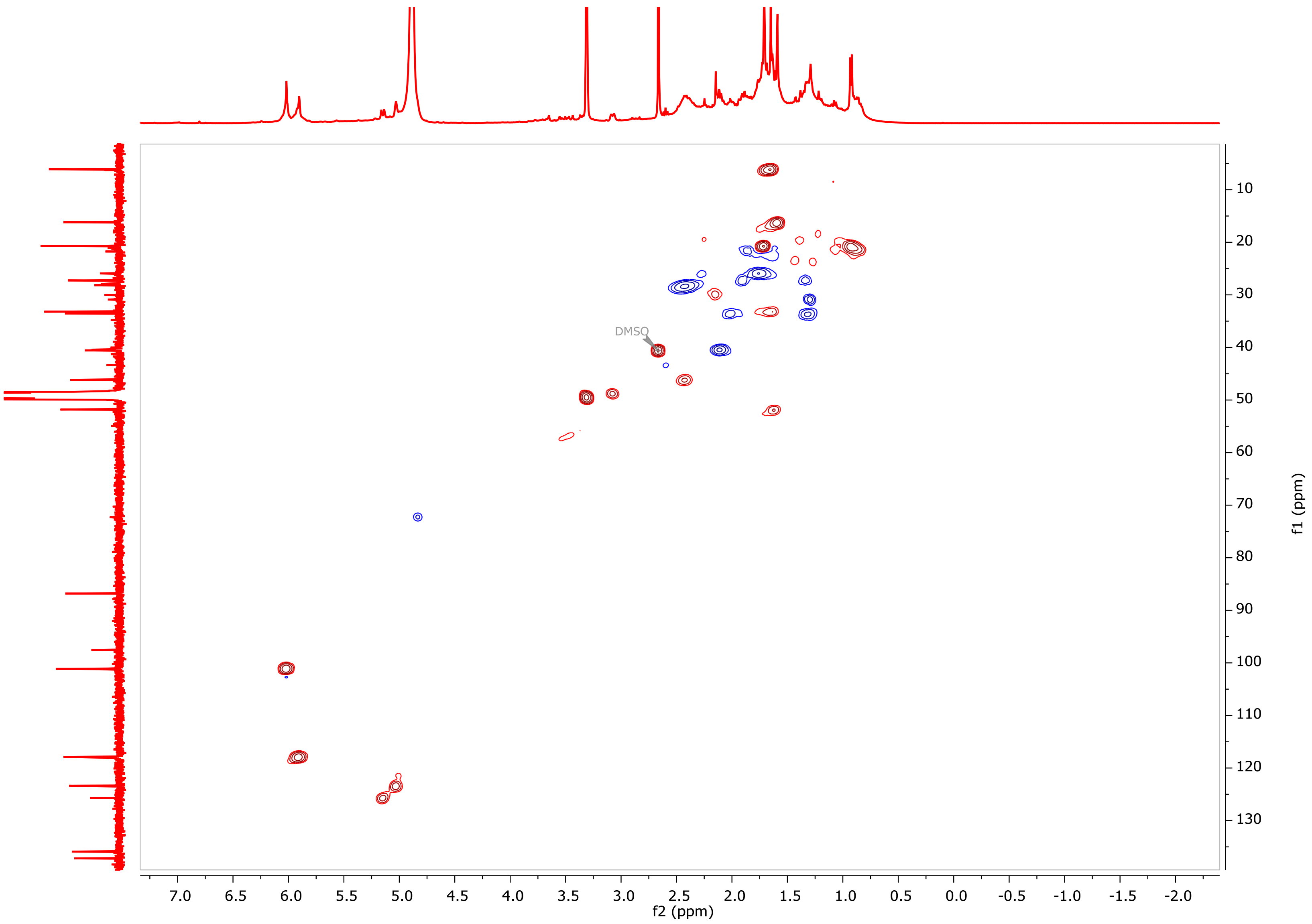

Figure S49. 400 MHz multiplicity edited ^{1}H-^{13}C HSQC NMR spectrum of ircinianin lactone C (**8**, 100%-fraction) in d_4-MeOH.

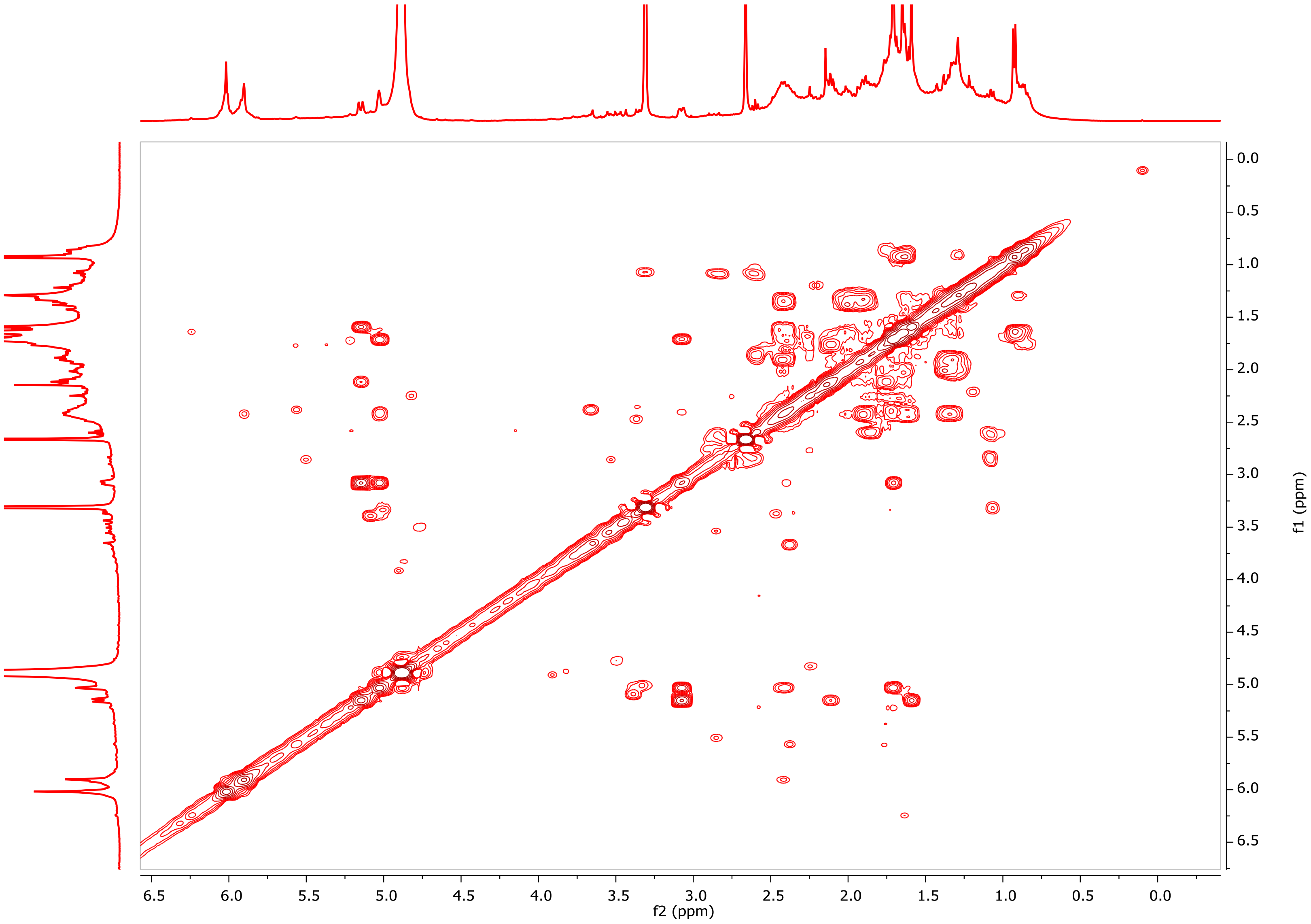

Figure S50. 400 MHz ^{1}H-^{1}H COSY NMR spectrum of ircinianin lactone C (**8**, 100%-fraction) in d_4-MeOH.

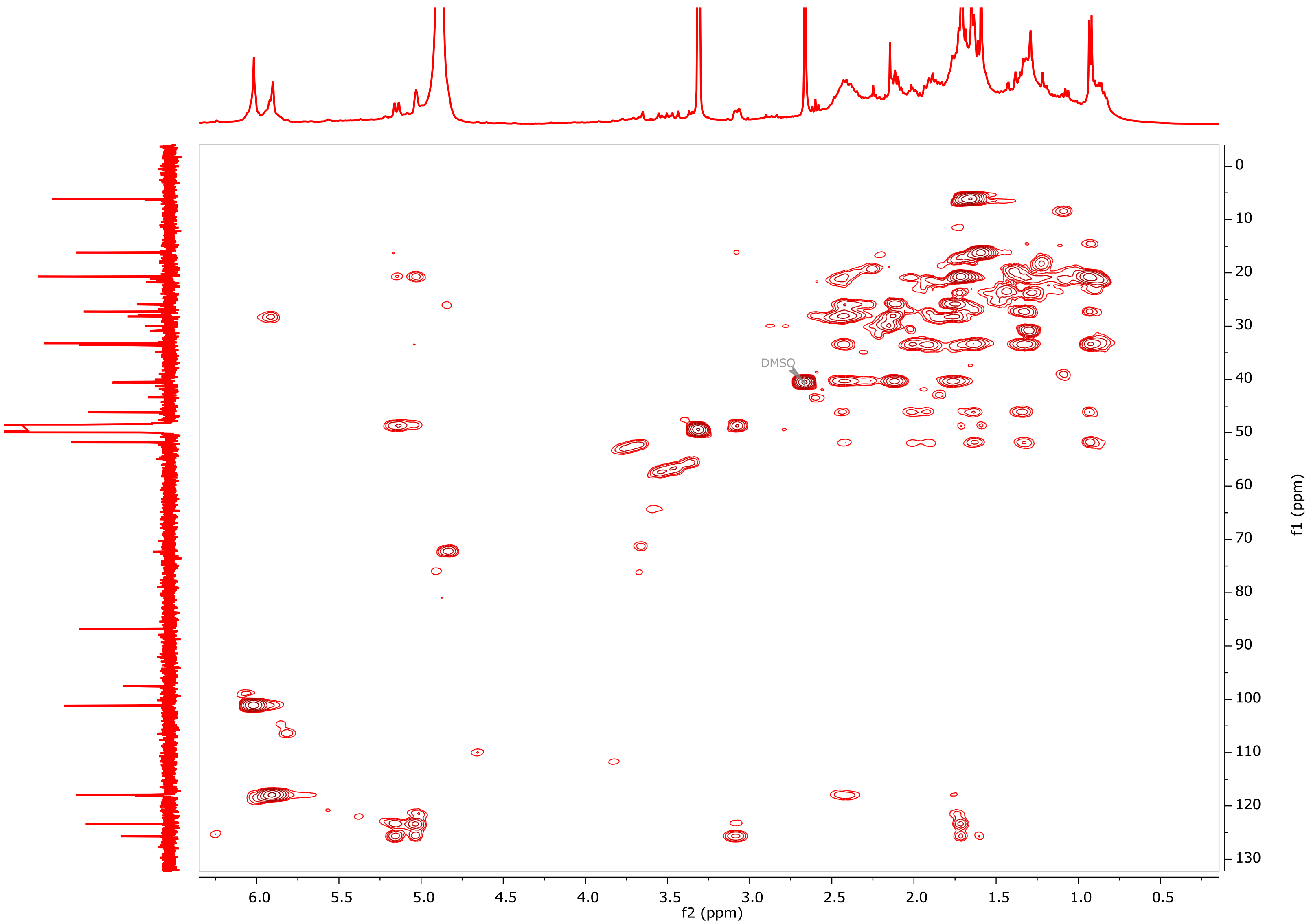

Figure S51. 400 MHz ^{1}H-^{13}C HSQC-TOCSY NMR spectrum of ircinianin lactone C (**8**, 100%-fraction) in d_4-MeOH.

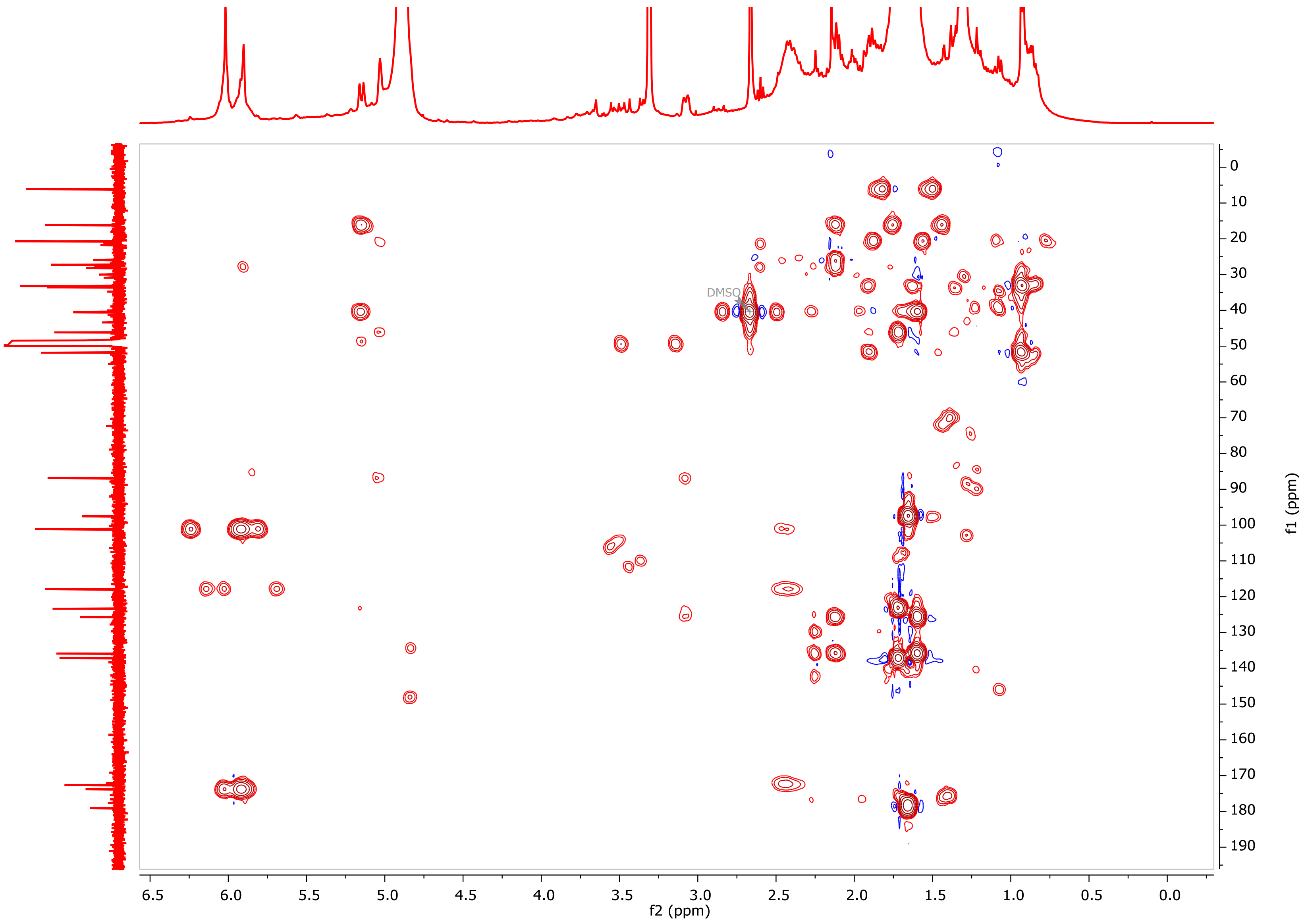

Figure S52. 400 MHz ^{1}H-^{13}C HMBC NMR spectrum of ircinianin lactone C (**8**, 100%-fraction) in d_4-MeOH.

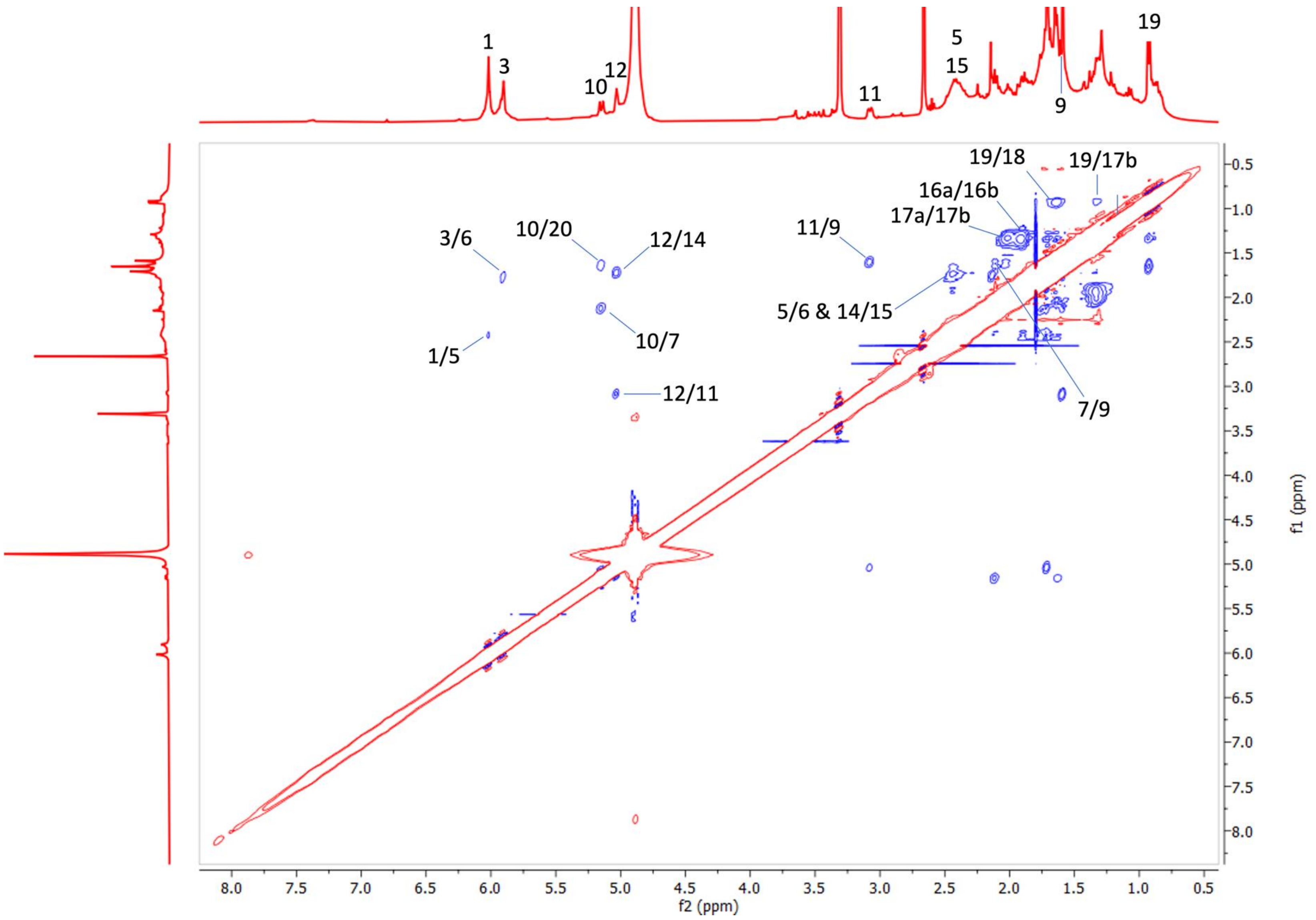

Figure S53. 400 MHz ^{1}H-^{1}H NOESY NMR spectrum of ircinianin lactone C (**8**, 100%-fraction) in d_4-MeOH.

Table S6. Results of the antimicrobial assays for ircinianin (**1**).

**Antibacterial assay**	
**Bacterial strain**	**MIC [µg/mL]**
E. faecium BM4147-1	>32
S. aureus ATCC29213	>32
K. pneumoniae ATCC12657	>32
A. baumannii 09987	>32
P. aeruginosa ATCC27853	>32
E. aerogenes ATCC13048	>32
E. coli ATCC25922	>32
B. subtilis 168	>32
M. smegmatis mc^2 155	>32

Figure S54. Results of the antiviral assay. Caco-2 cells (SARS-CoV-2) or HFF (HCMV) were infected as detailed in the M&M section. Cellular nuclei were stained with DAPI to visualize single cells and to evaluate the total number of cells. In the GFP/YFP channel infection is visualized by expression of mNeonGreen (SARS-CoV-2) or GFP (HCMV) via the viral reporter system, allowing to quantify infection levels. Images were taken with a Cytation3 multiplate reader. Images were colored with ImageJ. The scale bar represents 1mm. Evaluation of potential antiviral activity of ircinianin (10 µM) against (**A**) SARS-CoV2 or (**B**) HCMV.

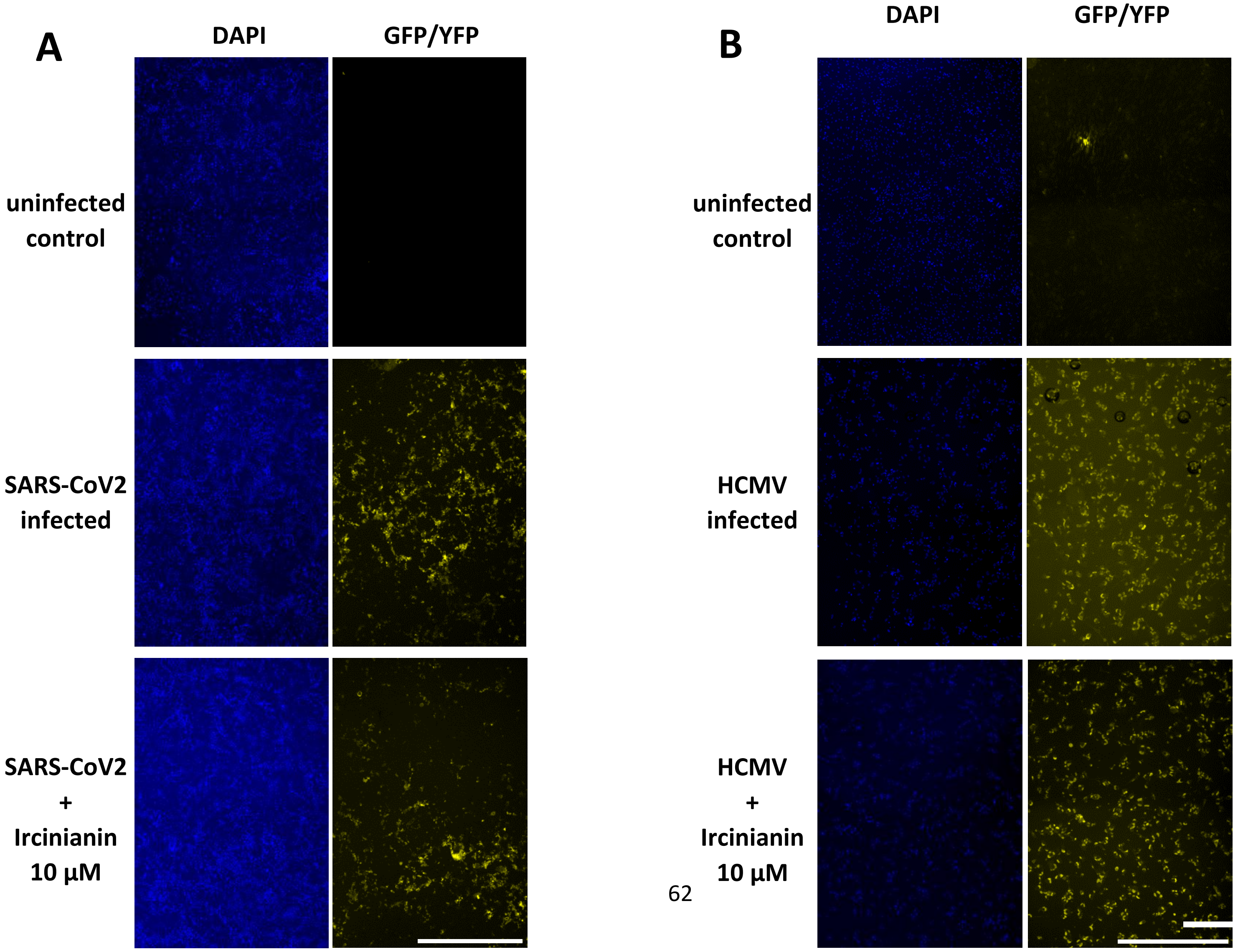

Table S7. Cytotoxocity Assays: TOP: Developmental Therapeutics Program (DTP)-One dose Mean Graph NCI-60 data for Ircinianin (**1**). BOTTOM: Single cell-line assays.

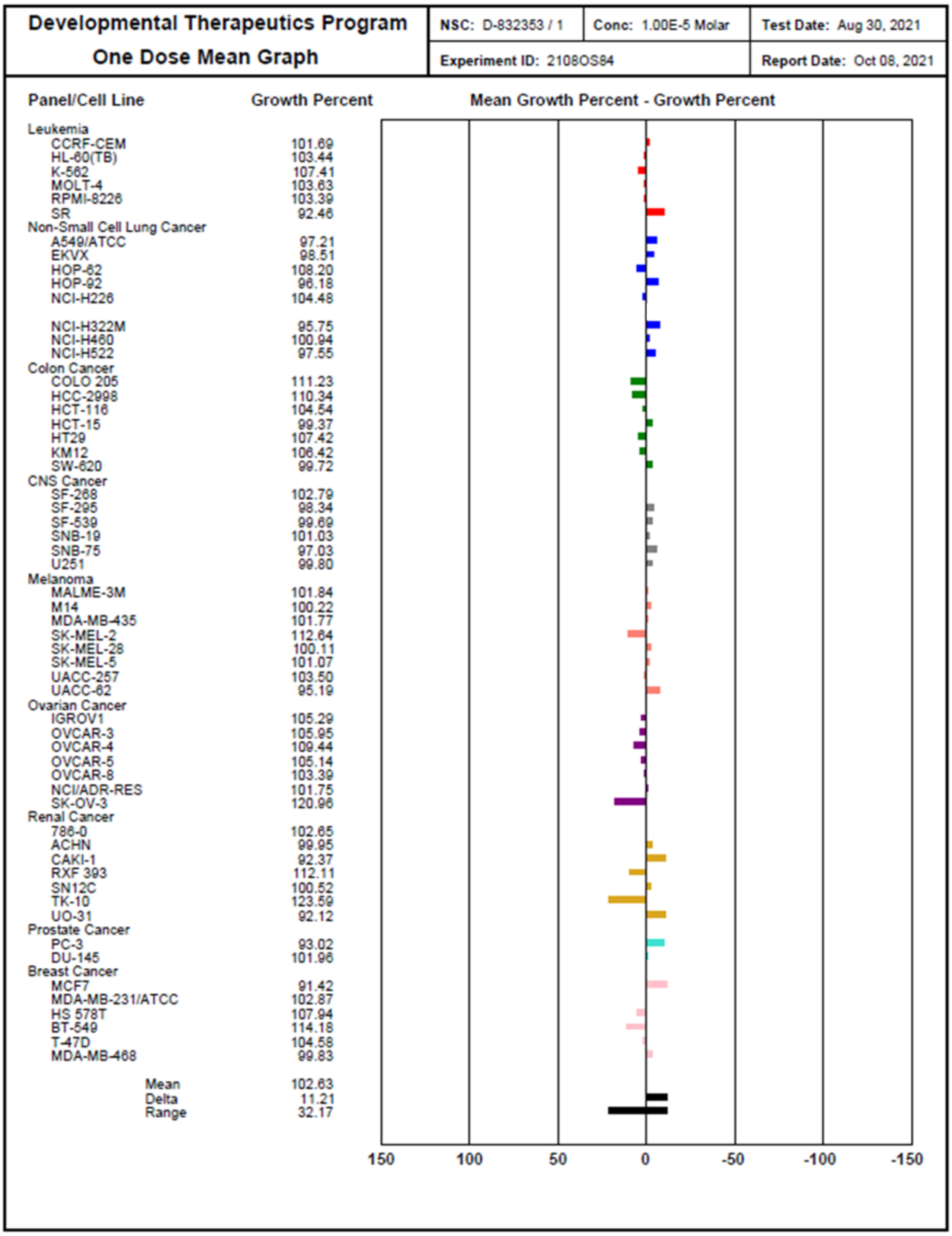

Single cell line assays	
Cell line	**IC_{50} [µg/mL]**
HeLa	>64
L6	59.5
